# Supplementary material for: Lenalidomide derivatives and proteolysis-targeting chimeras for controlling neosubstrate degradation
Source: Nat Commun. 2023 Aug 18;14:4683. doi: 10.1038/s41467-023-40385-9 (PMC10439208; doi:10.1038/s41467-023-40385-9)
Supplement: Supplementary file 1 — Supplementary Information [file 41467_2023_40385_MOESM1_ESM.pdf]

Supplementary Information:

Lenalidomide derivative and proteolysis-targeting chimeras for controlling neosubstrate degradation

Satoshi Yamanaka[1,2], Hirotake Furihata[1,3], Yuta Yanagihara[4], Akihito Taya[5], Takato Nagasaka[5], Mai Usui[5], Koya Nagaoka[1], Yuki Shoya[1], Kohei Nishino[6], Shuhei Yoshida[4], Hidetaka Kosako[6], Masaru Tanokura[3], Takuya Miyakawa[3,7], Yuuki Imai[4], Norio Shibata[5], Tatsuya Sawasaki[1*]

[1]Division of Cell-Free Sciences, Proteo-Science Center, Ehime University, Matsuyama, 790-8577 Japan, [2]Division of Proteo-Interactome, Proteo-Science Center, Ehime University, Matsuyama, 790-8577 Japan, [3]Department of Applied Biological Chemistry, Graduate School of Agricultural and Life Sciences, The University of Tokyo, Tokyo 113-8657, Japan, [4]Division of Integrative Pathophysiology, Proteo-Science Center, Ehime University, Toon, 791-0295 Japan, [5]Department of Life Science and Applied Chemistry, Nagoya Institute of Technology, Nagoya, 466-8555 Japan, [6]Division of Cell Signaling, Fujii Memorial Institute of Medical Sciences, Tokushima University, Tokushima, 770-8503, Japan, [7]Graduate School of Biostudies, Kyoto University, Kyoto, 606-8502, Japan

*Corresponding Author: Tatsuya Sawasaki
Proteo-Science Center, Ehime University, Matsuyama 790-8577, Japan
Tel: 81-89-927-8530
Fax: 81-89-927-9941
E-mail address: sawasaki@ehime-u.ac.jp.

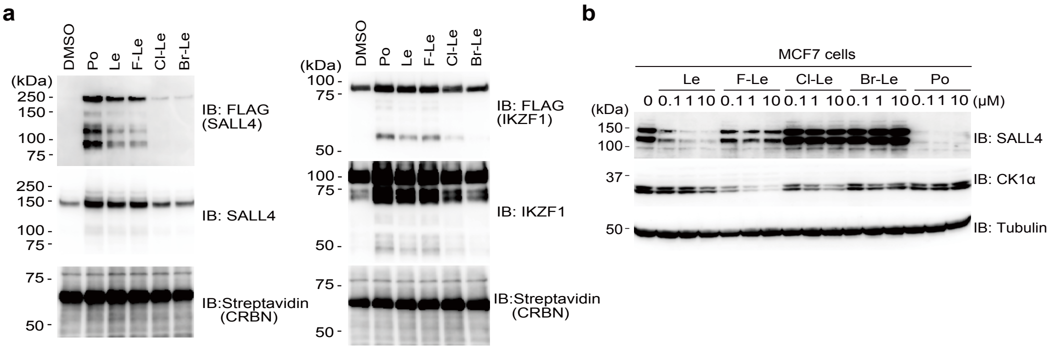

Supplementary Fig. 1. Biochemical and cell-based analyses of 6-position modification with a halogen atom on lenalidomide. a, *In vitro* binding assay using recombinant proteins. Complex formation between biotinylated CRBN and FLAG-GST-SALL4 or IKZF1 in the presence of pomalidomide (Po), lenalidomide (Le), or 6-position-modified Le (6-fluoro, F-Le; 6-chloro, Cl-Le; 6-bromo, Br-Le) was analysed using immunoblotting after a streptavidin pull-down assay. The experiment was repeated twice independently, with similar results. **b,** Immunoblot analysis of dose-dependent neosubstrate degradation in MCF7 cells. MCF7 cells were treated with DMSO, Po, Le, F-Le, Cl-Le, or Br-Le for 24 h, and the protein expression levels of neosubstrates were analysed using immunoblotting. The experiment was independently repeated thrice, with similar results. Source data are provided as a Source data file.

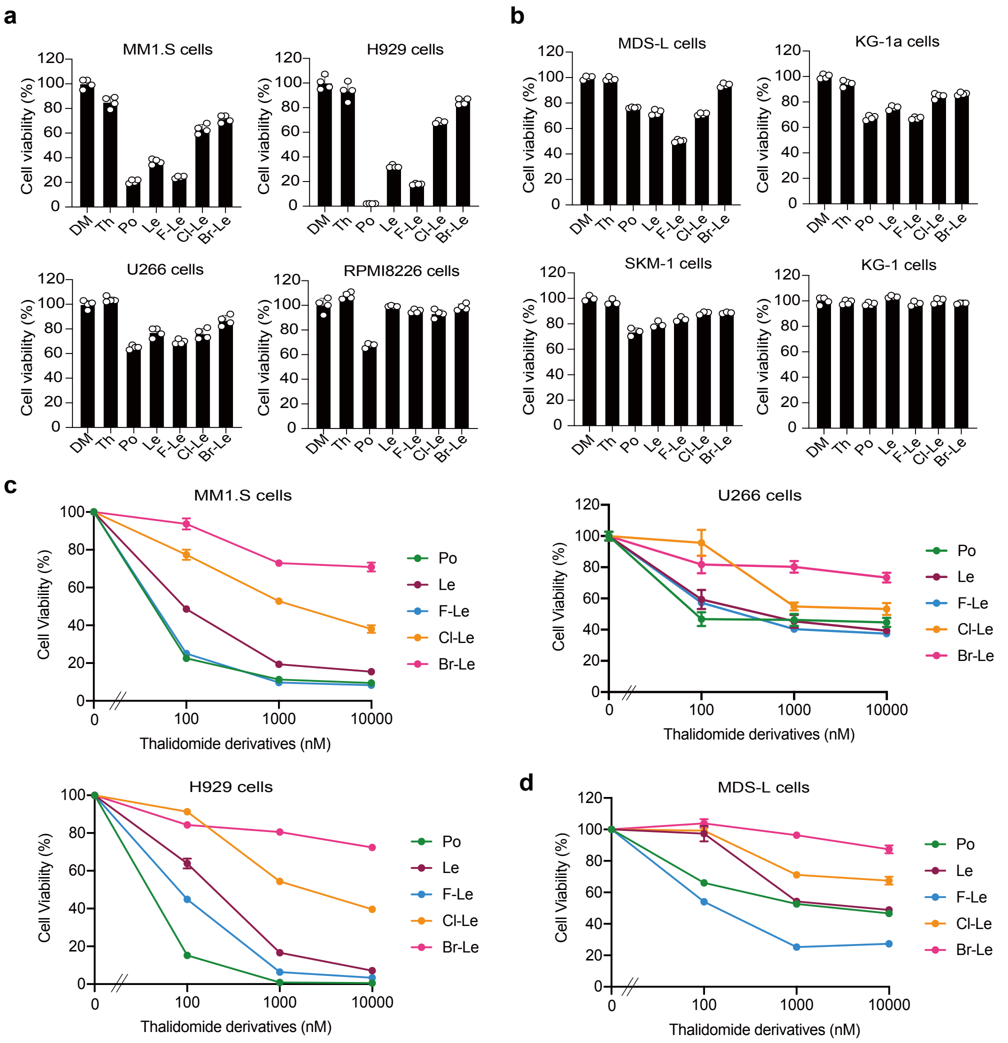

Supplementary Fig. 2. Anti-proliferative effects of 6-position-modified lenalidomide on multiple myeloma and myelodysplastic syndromes. a–b, Anti-proliferative effect of lenalidomide derivatives on (**a**) multiple myeloma (MM) and (**b**) myelodysplastic syndrome (MDS) cell lines. MM1.S, H929, U266, RPMI8226, MDS-L, KG-1a, SKM-1, and KG-1 cells were treated with DMSO, 1 µM Th, 1 µM Po, 1 µM Le, 1 µM F-Le, 1 µM Cl-Le, or 1 µM Br-Le for six days (MM1.S, H929, U266, RPMI8226, KG-1a, SKM-1, and KG-1 cells) or 12 days (MDS-L cells), and cell viability was analysed using Cell-Titer-Glo assay kit. Cell viability was expressed as the luminescence signal relative to the luminescence signal of DMSO, which was considered 100. Error bars denote standard deviation (biological replicates; n = 4). **c,** Anti-proliferative effects of lenalidomide derivatives on MM cell lines. MM1.S, H929 or U266 cells were treated with DMSO, Po,

Le, F-Le, Cl-Le, or Br-Le for nine days, and cell viability was analysed using the CellTiter-Glo assay kit. Cell viability was expressed as the luminescence signal relative to the luminescence signal of DMSO, which was considered 100. Error bars denote standard deviation (biological replicates; n = 4). **d,** Dose-dependent anti-proliferative effect of lenalidomide derivatives on 5q MDS cell lines. MDS-L cells were treated with DMSO, Po, Le, F-Le, Cl-Le, or Br-Le for 20 days, and cell viability was analysed using the CellTiter-Glo assay kit. Cell viability was expressed as the luminescence signal relative to the luminescence signal of DMSO, which was considered 100. Error bars denote standard deviation (biological replicates; n = 4). Source data are provided as a Source data file.

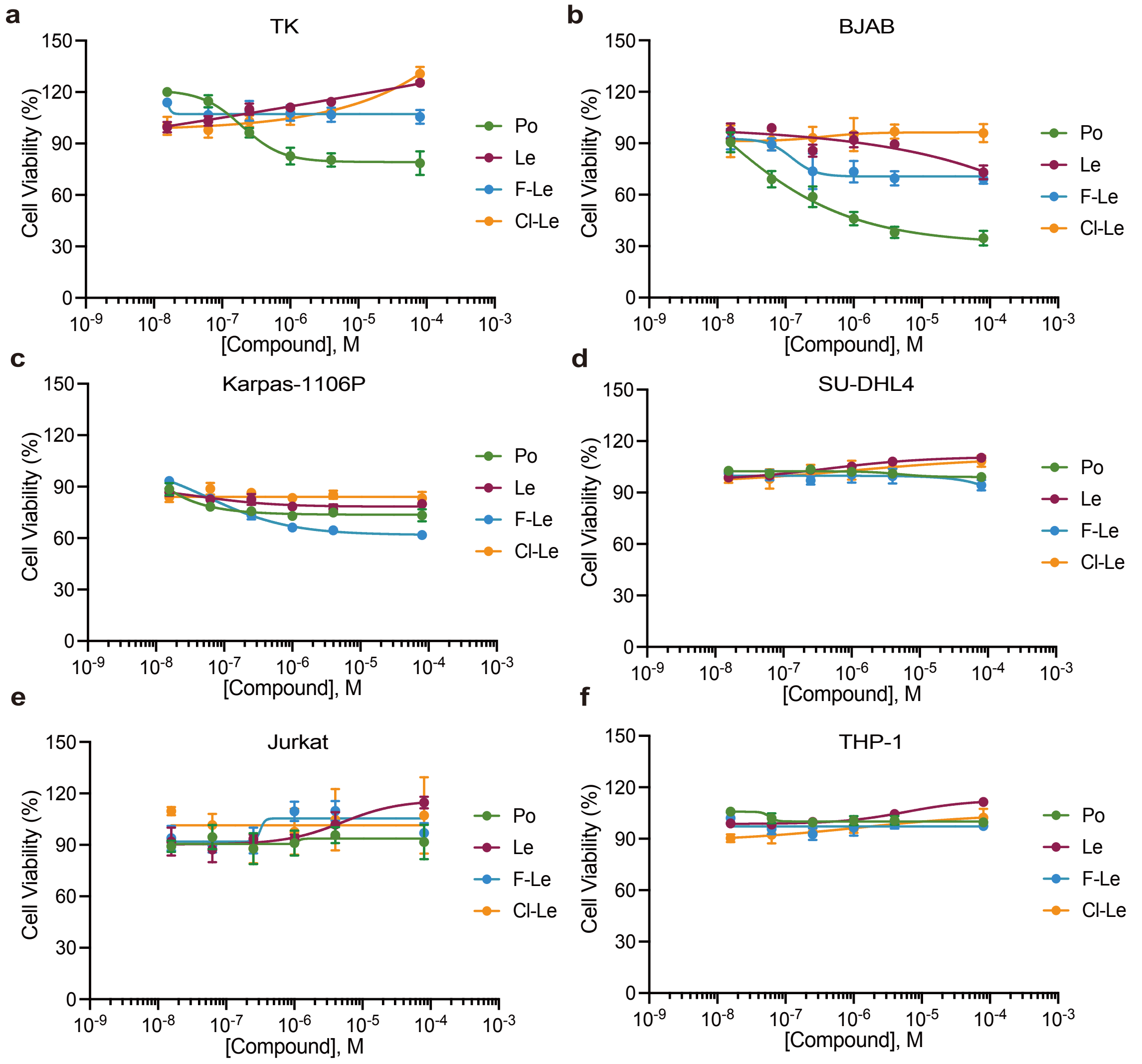

Supplementary Fig. 3. Anti-proliferative effects of 6-position-modified lenalidomides on various types of haematological cancer cell lines. a–f, Anti-proliferative effect of lenalidomide derivatives on (**a**) B-cell non-Hodgkin lymphoma, (**b**) Burkitt lymphoma, (**c**) primary mediastinal DLBCL, (**d**) germinal centre B cell-like DLBCL, (**e**) childhood T acute lymphoblastic leukaemia, and (**f**) childhood acute monocytic leukaemia. TK, BJAB, Karpas-1106P, SU-DHL4, Jurkat, or THP-1 cells were treated with DMSO, Po, Le, F-Le, and Cl-Le for 5 days at the indicated concentrations, and cell viability was analysed using Cell-Titer-Glo assay kit. Cell viability was expressed as the luminescence signal relative to the luminescence signal of DMSO, which was considered 100. Error bars denote standard deviations (biological replicates; n = 3). Source data are provided as a Source data file.

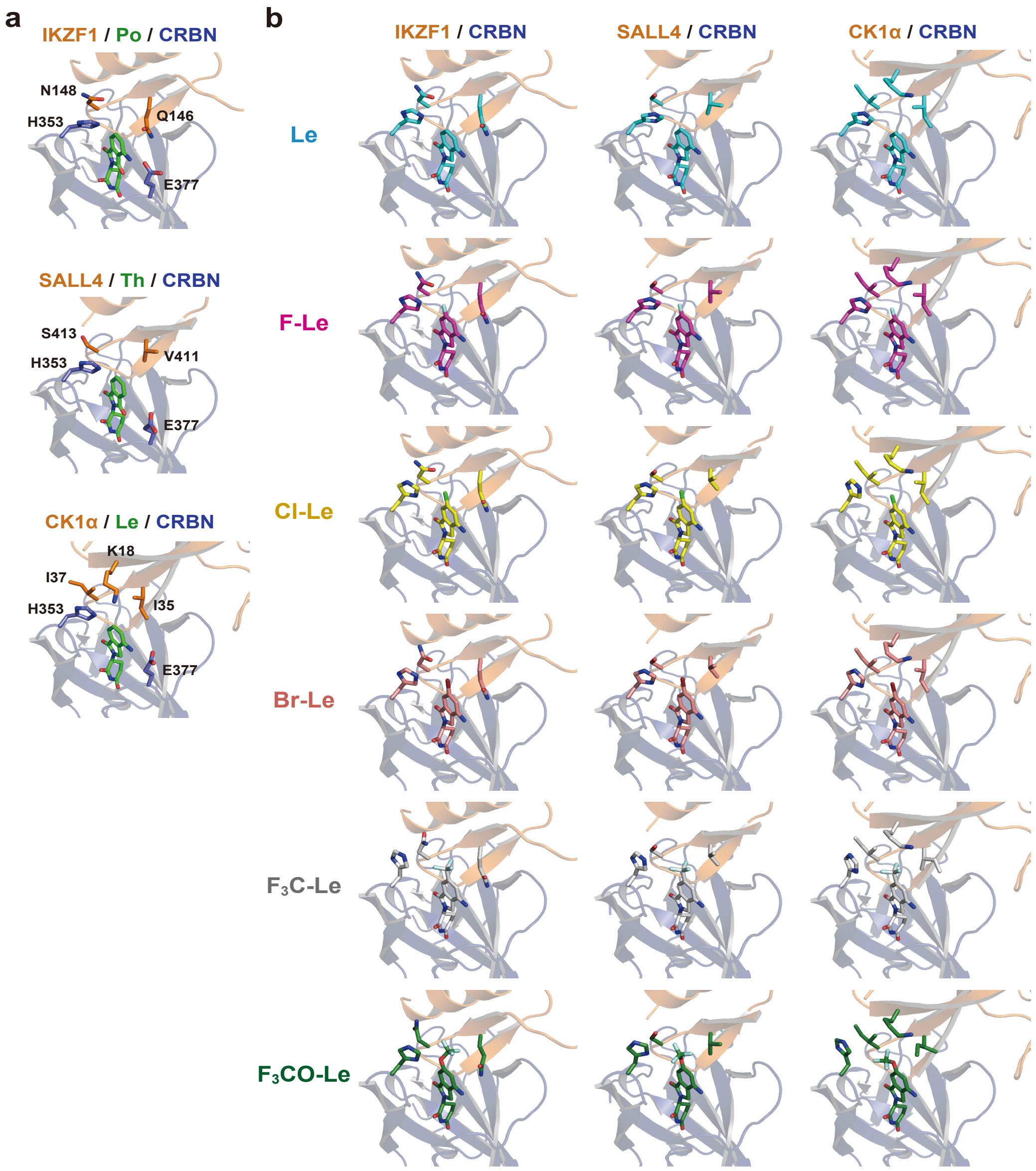

Supplementary Fig. 4. Putative binding modes of lenalidomide derivatives in the IMiD binding site of human CRBN.

a, The binding modes of pomalidomide (Po), thalidomide (Th), and lenalidomide (Le) to the IKZF1-CRBN (PDB ID: 6H0F)[39], SALL4-CRBN (PDB ID: 7BQU)[35], and CK1α-CRBN complexes (PDB ID: 5FQD)[33], respectively, which were determined using X-ray crystallography. **b,** Docking models of lenalidomide (Le) and five 6-position-modified Le (6-fluoro, F-Le; 6-chloro, Cl-Le; 6-bromo, Br-Le; 6-trifluoromethyl, F3C-Le; and 6-trifluoromethoxy, F3CO-Le). Each compound and the residues located around the 6-position of the phthalimide ring are shown in the stick model. These residues were set to

adopt a variable side-chain conformation in the docking simulation with AutoDock Vina, and an orientation that allows for a substituent at position six is presented in each docking model. Source data are provided as a Source data file.

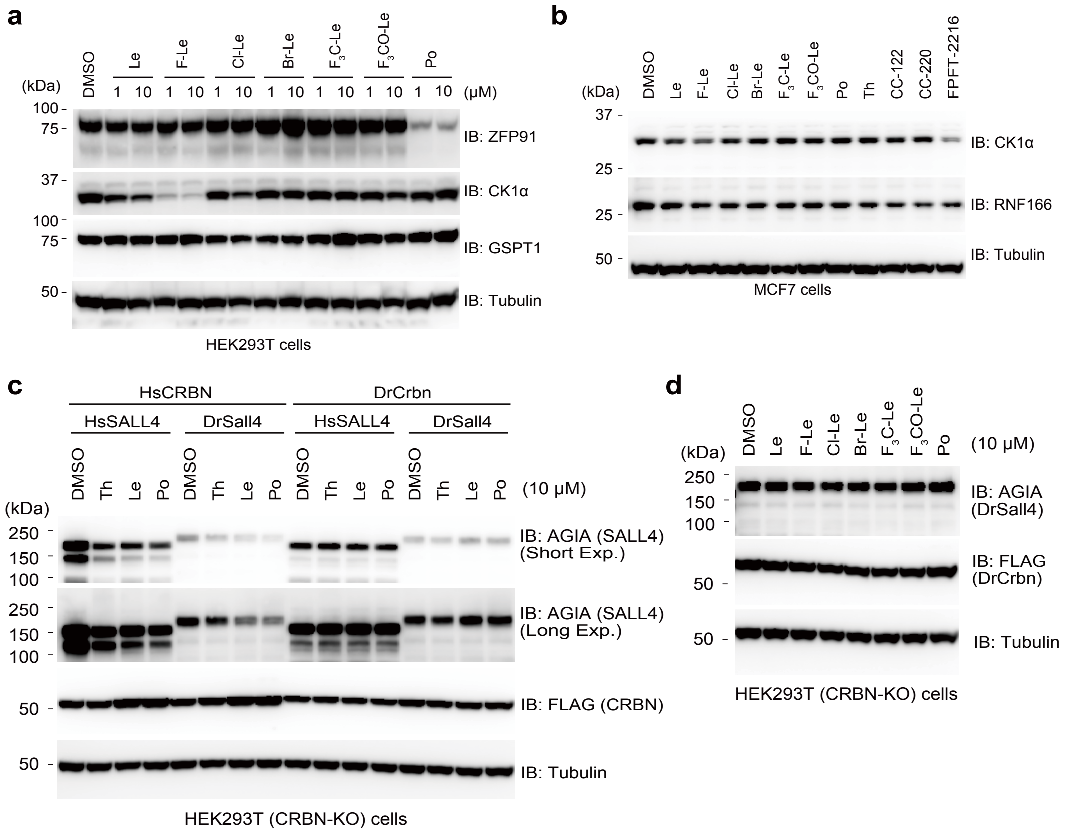

Supplementary Fig. 5. Protein degradation of neosubstrates by 6-position-modified lenalidomides.

a–b, Protein degradation of neosubstrates by 6-position-modified lenalidomides. (**a**) HEK293T or (**b**) MCF7 cells were treated with DMSO, Th, Po, Le, F-Le, Cl-Le, Br-Le, F_3C-Le, F_3CO-Le, CC-122, CC-220 or FPFT-2216 for 24 h, and cell lysates were analysed by immunoblotting. The experiment was repeated twice independently, with similar results. **c–d,** Immunoblot analysis of exogenous neosubstrate degradation by zebrafish protein expression. HEK293T (CRBN-KO) cells were transfected with pCAGGS-AGIA-HsSALL4 or -DrSall4 and pCAGGS-FLAG-HsCRBN or -DrCrbn, and treated with DMSO, (**c**) Th, Le, Po, or (**d**) 6-position-modified lenalidomides for 16 h. Protein expression levels of exogenous neosubstrates were analysed by immunoblotting. The experiment was independently repeated twice, with similar results. Source data are provided as a Source data file.

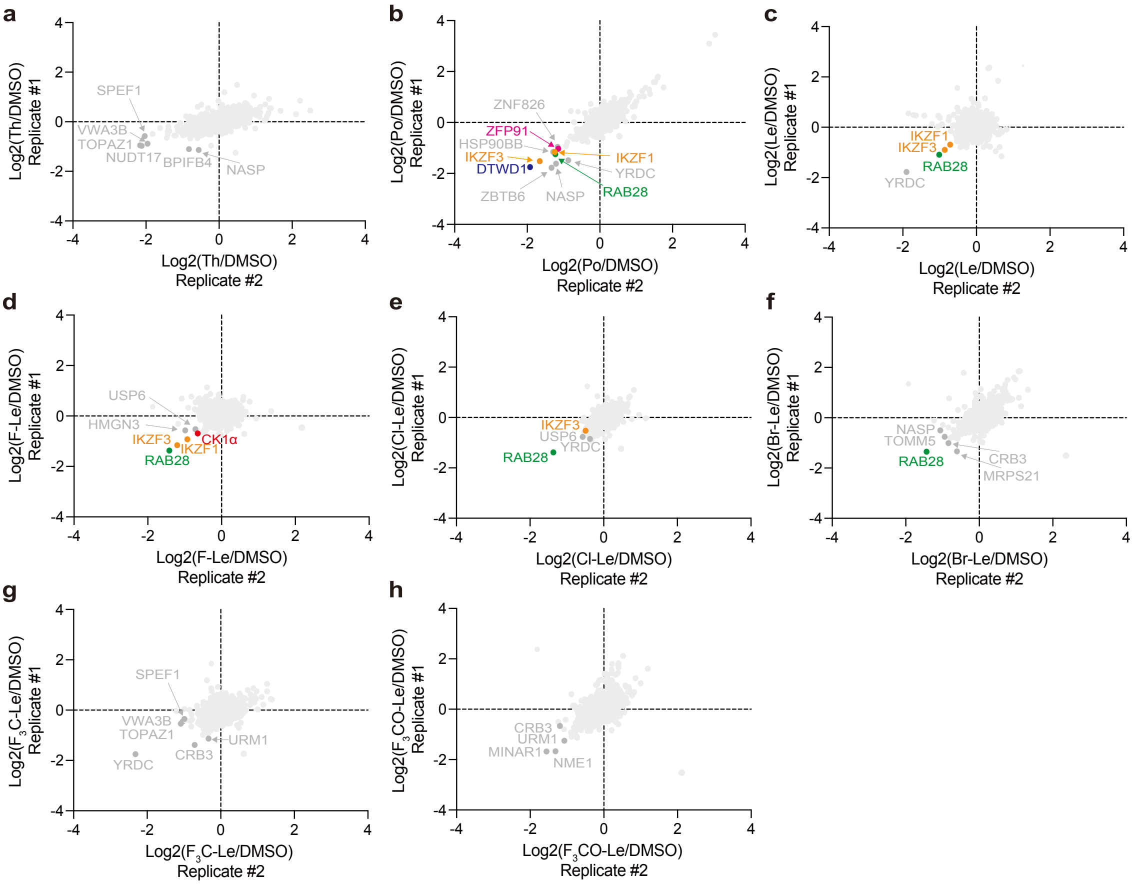

Supplementary Fig. 6. Global analysis of protein degradation by 6-position-modified lenalidomides.

a–h, LC-MS/MS analysis of TMT-labelled peptides using cell lysates treated with lenalidomide derivatives. MM1.S cells were treated with DMSO, (**a**) Th, (**b**) Po, (**c**) Le, (**d**) F-Le, (**e**) Cl-Le, (**f**) Br-Le, (**g**) F_3C-Le, or (**h**) F_3CO-Le cells for 5 h in two biological replicates. Total proteins in the cell lysates were quantified using TMT-based mass spectrometry. Source data are provided as a Supplementary Data 1.

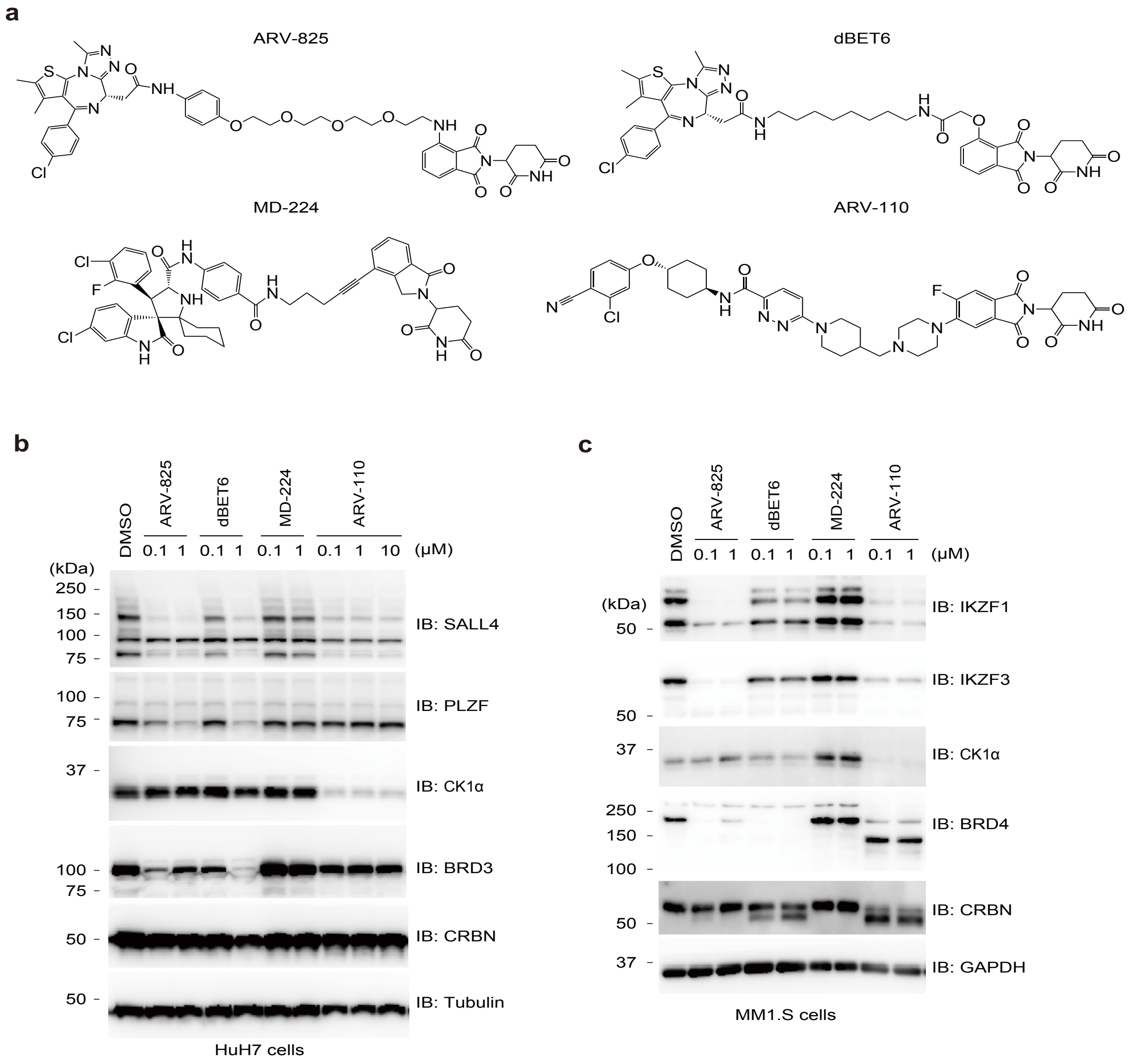

Supplementary Fig. 7. Protein degradation of the neosubstrate by IMiD-based PROTACs.

a, Chemical structures of ARV-825, dBET6, MD-224, and ARV-110. **b–c,** Neosubstrate selectivity of IMiD-based PROTACs using different linkers: (**b**) HuH7 cells or (**c**) MM1.S cells. HuH7 or MM1.S cells were treated with DMSO, ARV-825, dBET6, MD-224, or ARV-110 for 24 h, and the protein expression levels of neosubstrate were analysed by immunoblotting. The experiment was independently repeated thrice, with similar results. Source data are provided as a Source data file.

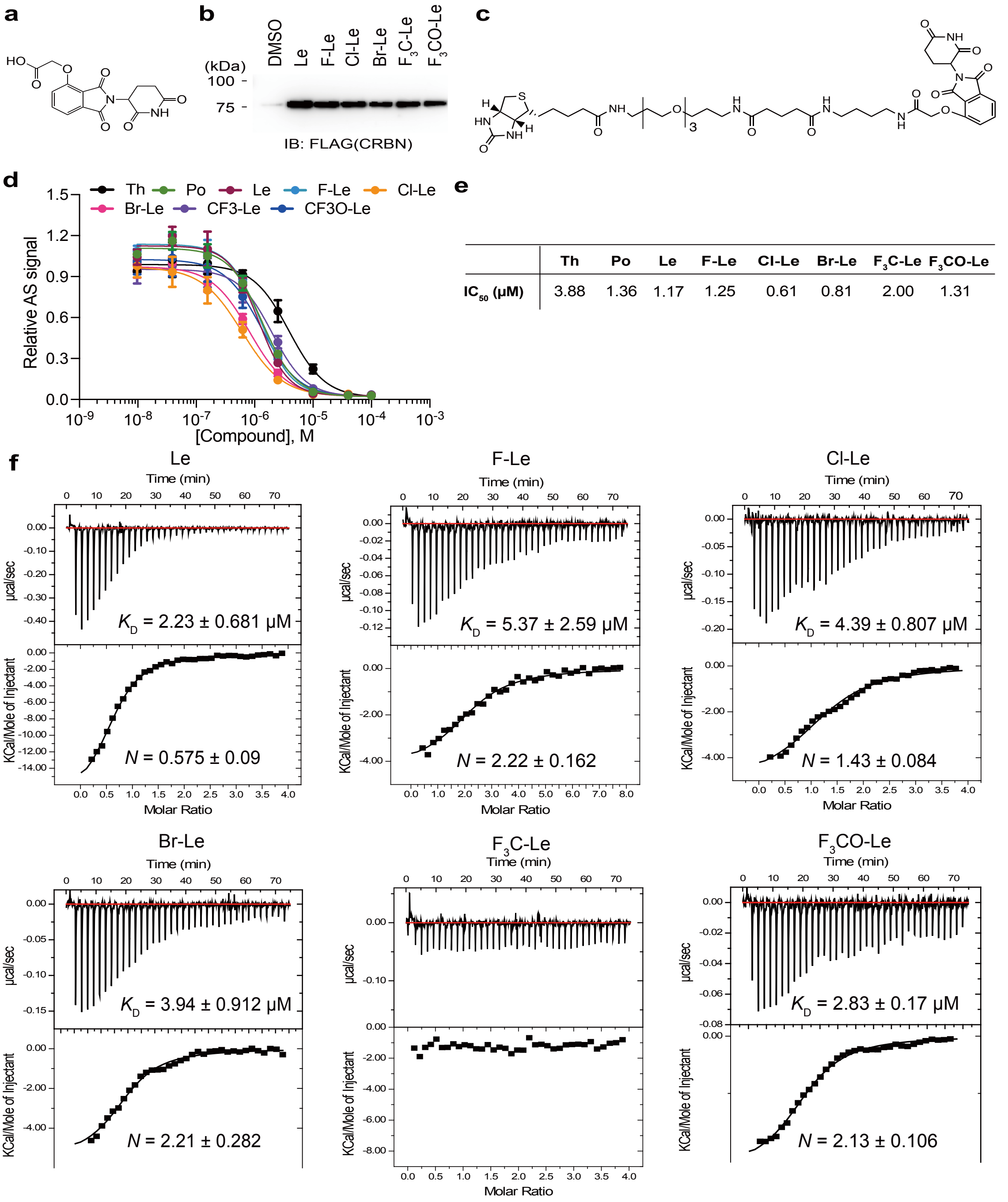

Supplementary Fig. 8. Characterization of the binding ability of 6-position-modified lenalidomides for CRBN.

a, Chemical structures of thalidomide derivatives immobilised on magnetic beads. **b,** Evaluation of the binding ability of 6-position-modified lenalidomides using thalidomide-immobilized beads. FLAG-GST-CRBN was pulled down using thalidomide-immobilized magnetic beads and competitively eluted with DMSO, 200 μM Le, 200 μM F-Le, 200 μM Cl-Le, 200 μM Br-Le, 200 μM F_3C-Le, or 200 μM F_3CO-Le. The experiment was

independently repeated thrice, with similar results. **c,** Chemical structures of biotinylated thalidomide for AlphaScreen-based competitive assay. **d,** Evaluation of the binding ability of 6-position-modified lenalidomides using AlphaScreen-based competitive assay. Interaction between biotinylated thalidomide and FLAG-GST-CRBN-CTD (residues 318–442) were competitively inhibited by Th, Po, Le, F-Le, Cl-Le, Br-Le, F_3C-Le, or F_3CO-Le and was analysed using AlphaScreen (AS) technology. The relative AS signals are expressed as the luminescence signal relative to the luminescence signal of DMSO, which is considered 1. Error bars denote standard deviations (independent experiments, n = 3). **e,** The half-maximal inhibitory concentration (IC_{50}) values were calculated using dose-response curves in Supplementary Fig. 8d. **f,** Isothermal titration calorimetry experiments of the CRBN thalidomide-binding domain (TBD) titrated with Le, F-Le, Cl-Le, Br-Le, F_3C-Le, or F_3CO-Le. The values of the dissociation constant (K_D) and molar binding ratio (*N*) were calculated using the mean values of standard deviation (*n* = 3 independent experiments). Source data are provided as a Source data file.

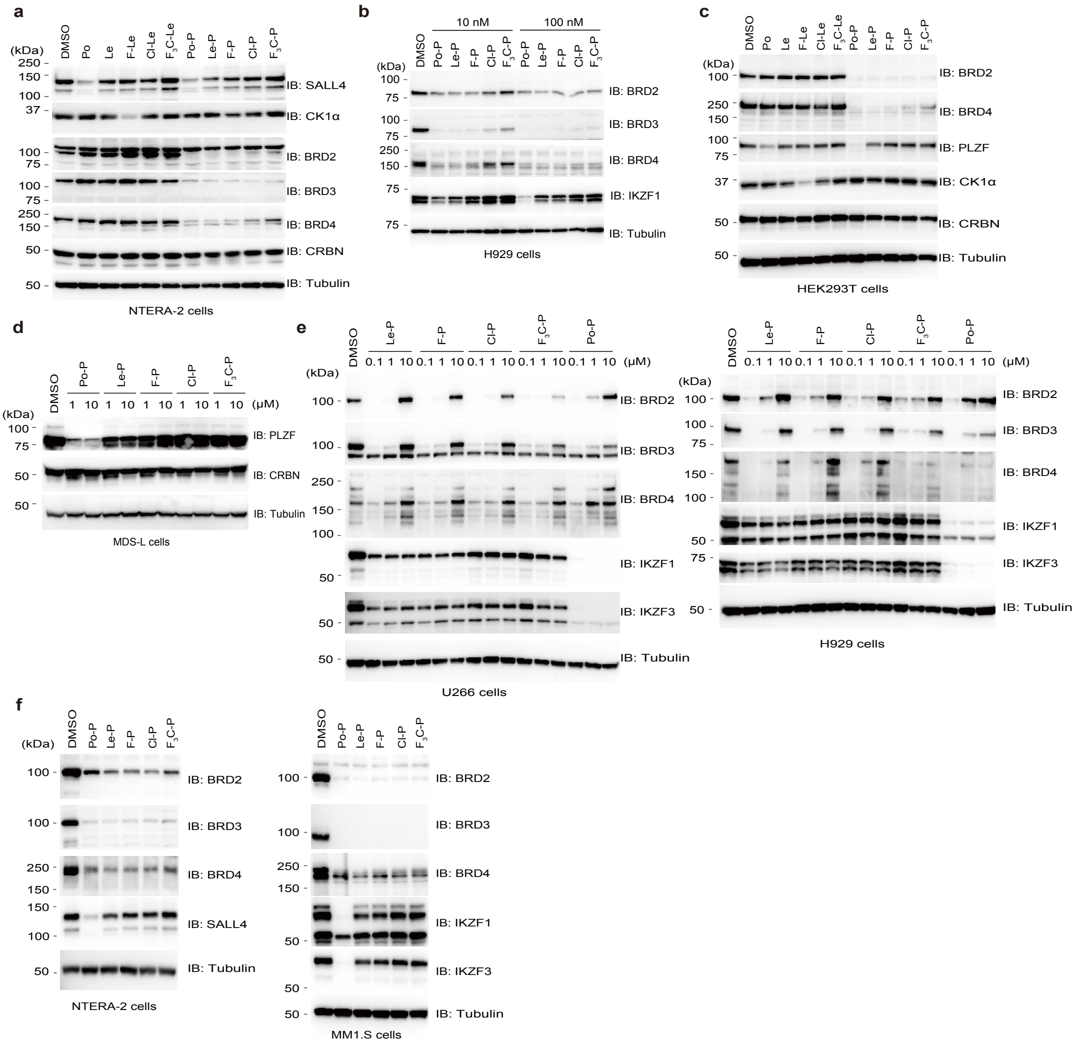

Supplementary Fig. 9. Degradation of BET proteins and neosubstrates by PROTACs based on 6-position-modified lenalidomides.

a, Protein degradation of lenalidomide derivatives and PROTACs based on 6-position-modified lenalidomides. NTERA-2 cells were treated with DMSO, 0.1 μM Po, 0.1 μM Le, 0.1 μM F-Le, 0.1 μM Cl-Le, 0.1 μM F_3C-Le, 0.1 μM ARV-825/Po-P, 0.1 μM lenalidomide-based PROTAC/Le-P, 0.1 μM F-Le-based PROTAC/F-P, 0.1 μM Cl-Le-based PROTAC/Cl-P, or 0.1 μM F_3C-Le-based PROTAC/F_3C-P for 24 h, and protein expression levels of BET proteins, SALL4, and CK1α were analysed using immunoblot. The experiment was independently repeated thrice, with similar results. **b,** BET protein degradation by PROTACs based on 6-position-modified lenalidomides at low concentrations. H929 cells were treated with DMSO, Po-P, Le-P, F-P, Cl-P, or F_3C-P for 24 h, and protein expression levels of BET proteins and IKZF1 were analysed using

immunoblotting. The experiment was independently repeated thrice, with similar results. **c–e,** Neosubstrate selectivity of PROTACs based on 6-position-modified lenalidomides. (**c**) HEK293T, (**d**) MDS-L, or (**e**) U266 and H929 cells were treated with DMSO, Po-P, Le-P, F-P, Cl-P, or F_3C-P for 24 h, and the protein expression levels of BET proteins, PLZF, IKZF1, IKZF3, and CK1α were analysed using immunoblotting. The experiment was independently repeated thrice, with similar results. **f,** Immunoblot analysis of cell lysates for TMT-based mass spectrometry. NTERA-2 and MM1.S cells were treated with DMSO, 0.3 μM Po-P, 0.3 μM Le-P, 0.3 μM F-P, 0.3 μM Cl-P, or 0.3 μM F_3C-P for 16 h, and protein expression levels were analysed using immunoblot. The experiment was independently repeated thrice, with similar results. Source data are provided as a Source data file.

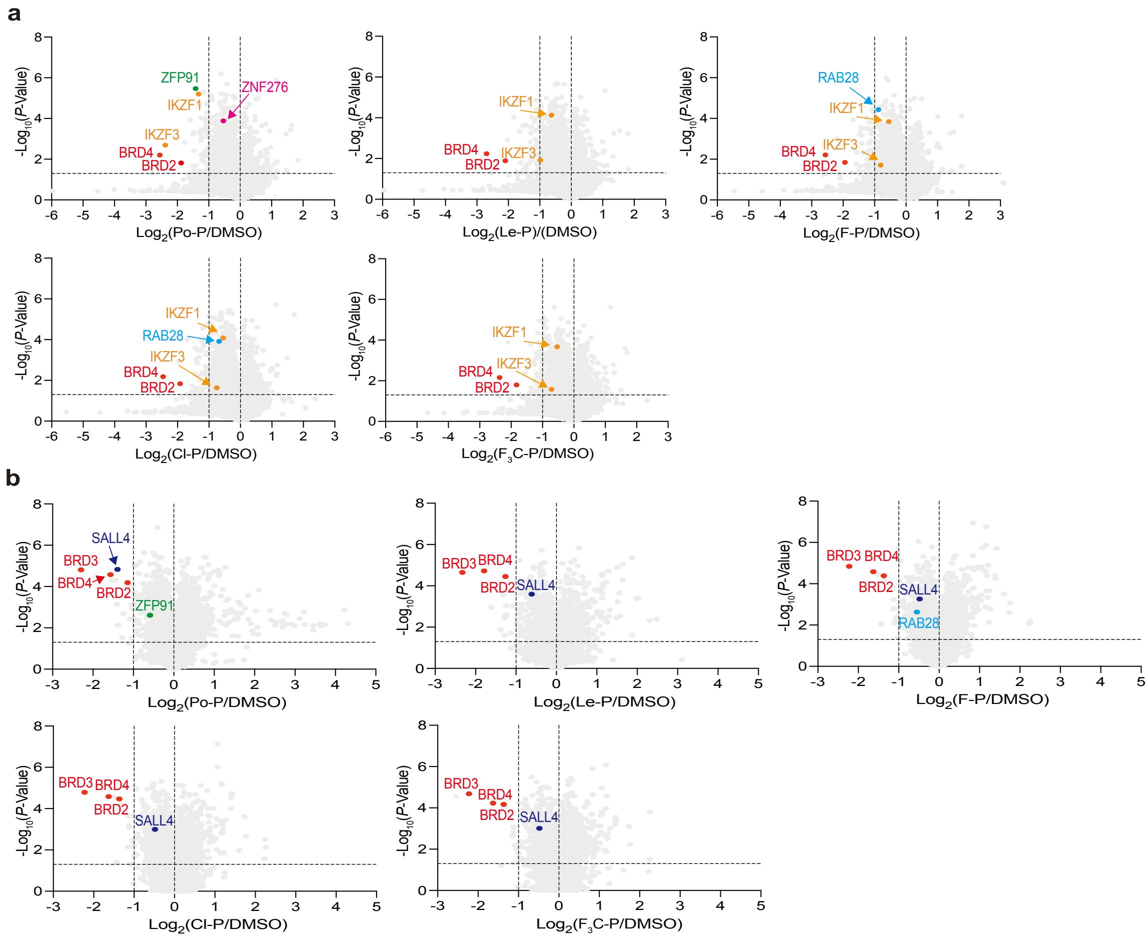

Supplementary Fig. 10. Global analysis of protein degradation by PROTACs using 6-position-modified lenalidomide.

a–b, LC-MS/MS analysis of TMT-labelled peptides using cell lysates treated with PROTACs. (**a**) MM1.S or (**b**) NTERA-2 cells were treated with DMSO, 0.3 µM Po-P, 0.3 µM Le-P, 0.3 µM F-P, 0.3 µM Cl-P, or 0.3 µM F_3C-P for 16 h in three biological replicates. Total proteins in the cell lysates were quantified by TMT-based mass spectrometry. Significant changes in the volcano plots were calculated using Student's two-sided t-test, and the false discovery rate (FDR)-adjusted *P*-values calculated using the Benjamini–Hochberg method are shown in Supplementary Data 2 and 3. Source data are provided as Supplementary Data 2 and 3.

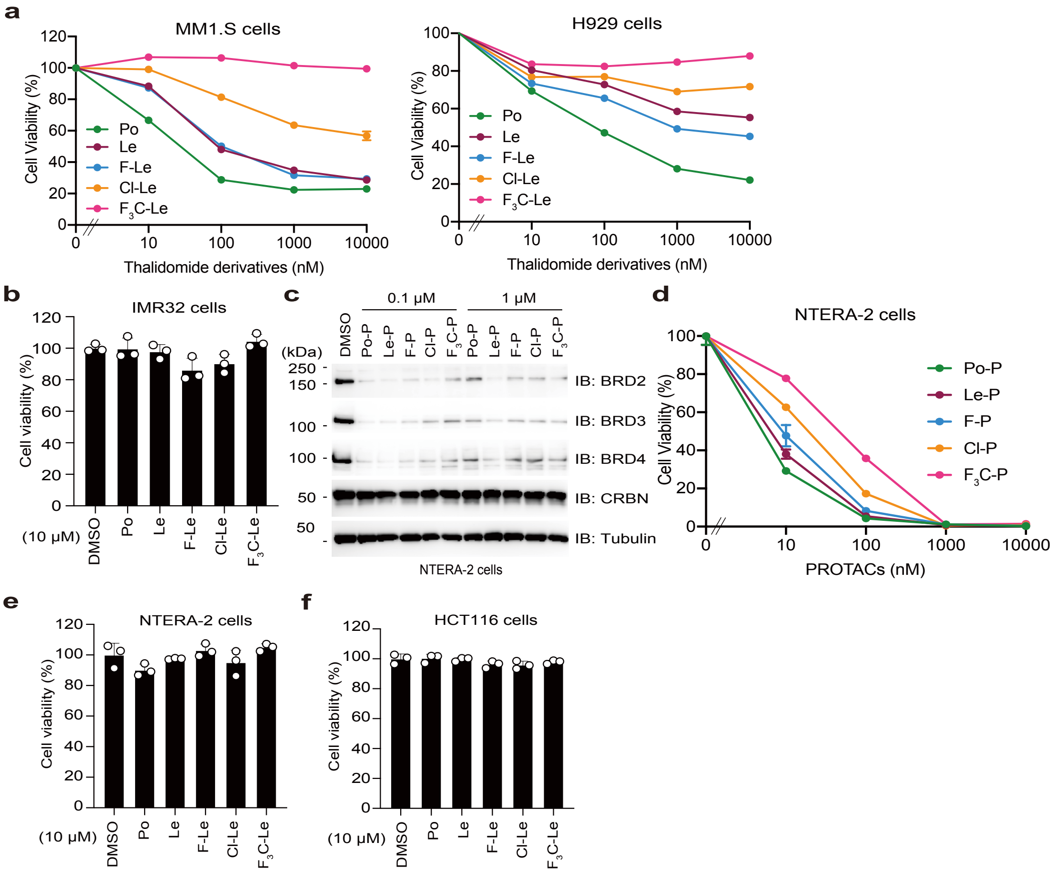

Supplementary Fig. 11. Anti-proliferative effect and degradation of BET proteins in diverse cancer cell lines.

a, Dose-dependent anti-proliferative effect of 6-position-modified lenalidomides on MM cell lines. MM1.S and H929 cells were treated with DMSO, Po, Le, F-P, Cl-P or F_3C-Le for five days, and cell viability was analysed using the CellTiter-Glo assay kit. Cell viability was expressed as the luminescence signal relative to the luminescence signal of DMSO, which was considered 100. Error bars denote standard deviation (biological replicates; n = 3). **b,** Anti-proliferative effect of lenalidomide derivatives on IMR32 cells. IMR32 cells were treated with DMSO, Po, Le, F-P, Cl-P or F_3C-Le for five days, and cell viability was analysed using the CellTiter-Glo assay kit. Cell viability was expressed as the luminescence signal relative to the luminescence signal of DMSO, which was considered 100. Error bars denote standard deviation (biological replicates; n = 3). **c,** Immunoblot analysis of BET proteins in pluripotent human embryonal carcinoma. NTERA-2 cells were treated with DMSO, Po-P, Le-P, F-P, Cl-P or F_3C-P for 24 h, and the expression levels of BET proteins were analysed by immunoblotting. The experiment was independently repeated thrice, with similar results. **d,** Dose-dependent anti-

proliferative effects of PROTACs based on 6-position-modified lenalidomides on pluripotent human embryonal carcinoma. NTERA-2 cells were treated with DMSO, Po-P, Le-P, F-P, Cl-P or F_3C-P for three days, and cell viability was analysed using the Cell Titer-Glo assay kit. Cell viability was expressed as the luminescence signal relative to the luminescence signal of DMSO, which was considered 100. Error bars denote standard deviation (biological replicates; n = 3). **e–f,** Anti-proliferative effects of 6-position-modified lenalidomides on NTERA-2 and HCT116 cells. (**e**) NTERA-2 or (**f**) HCT116 cells were treated with DMSO, Po, Le or 6-position-modified lenalidomides for three days (NTERA-2 cells) or two days (HCT116 cells), and cell viability was analysed using the CellTiter-Glo assay kit. Cell viability was expressed as the luminescence signal relative to the luminescence signal of DMSO, which was considered 100. Error bars denote standard deviation (biological replicates; n = 3). Source data are provided as a Source data file.

Supplementary Table 1. Affinity scores of top three docking models of lenalidomide and its 6-position derivatives with the 3D structures of CRBN complexed with neosubstrates (IKZF1, SALL4, and CK1α)

		Affinity score (kcal mol^{-1})		
**Compounds**	**Rank**	**IKZF1**	**SALL4**	**CK1α**
Lenalidomide (Le)	1	**–11.5** *	**–10.9**	**–10.7**
	2	–9.0	–8.8	–9.0
	3	– #	–8.6	–8.7
F-Le	1	**–10.8**	**–10.0**	**–10.2**
	2	–9.4	–9.2	–9.2
	3	–9.1	–9.1	–8.6
Cl-Le	1	**–11.2**	**–10.2**	**–10.5**
	2	–10.0	–9.3	–8.8
	3	–8.8	–9.2	–8.8
Br-Le	1	**–11.0**	**–10.0**	**–10.2**
	2	–9.8	–9.2	–8.5
	3	–8.6	–8.0	–8.2
F_3C-Le	1	**–10.8**	–9.5	**–10.6**
	2	–10.0	–9.4	–9.1
	3	–9.5	**–9.3**	–9.0
F_3CO-Le	1	**–10.9**	**–10.0**	**–10.6**
	2	–10.0	–9.9	–9.2
	3	–9.6	–9.8	–9.0

* Models with docking affinity scores in bold are shown in Supplementary Fig. 4.

# This value is unavailable because only two docking models were simulated with AutoDock Vina calculation.

Supplementary methods – Organic chemistry

General information

All reactions were performed in oven-dried glassware under N_2 atmosphere unless otherwise mentioned. Solvents were transferred via syringe and were introduced into the reaction vessels. All the reactions were monitored by thin-layer chromatography (TLC) carried out on 0.25 mm Merck silica gel (60-F254). The TLC plates were visualized with UV light. Products were purified by column chromatography on columns packed with silica gel (60N spherical neutral size 64-210 μm) or washed with distilled water or organic solvents. The ^{1}H-NMR (300 MHz), ^{1}H-NMR (500 MHz), ^{19}F-NMR (282 MHz), ^{13}C-NMR (75 MHz), and ^{13}C-NMR (126 MHz) spectra were recorded for solution in $CDCl_3$, Acetone-d_6, Methanol-d_4 or DMSO-d_6 on a Varian Mercury 300 and a Bruker Avance 500. The ^{13}C-NMR (176 MHz) spectra were recorded on a JEOL ECZ700R. Chemical shifts (δ) are expressed in ppm downfield from TMS (δ = 0.00 ppm), $CDCl_3$ (δ = 7.26 ppm), Acetone-d_6 (δ = 2.05 ppm), Methanol-d_4 (δ = 3.31 ppm) or DMSO-d_6 (δ = 2.50 ppm) for ^{1}H-NMR, C_6F_6 [δ = −162.2 ppm ($CDCl_3$ or DMSO-d_6)] for ^{19}F-NMR, and $CDCl_3$ (δ = 77.16 ppm), Acetone-d_6 [δ = 29.84 ppm (CH_3), 206.16 ppm (C=O)] or DMSO-d_6 (δ = 39.52 ppm) for ^{13}C-NMR respectively. Mass spectra were recorded on a SHIMADZU GCMS-QP5050A (EI-MS) and SHIMAZU LCMS-2020 (ESI-MS). High-resolution mass spectrometry (HRMS) was recorded on a Waters Synapt G2HDMS (ESI-TOF-MS) system, GCT Premier (EI-TOF-MS) system. Infrared spectra were recorded on a JASCO FT/IR-4100 spectrometer. Melting points were recorded on a BUCHI M-565. HPLC separations were performed on a JASCO PU-2080 Plus system using NOMURA CHEMICAL Develosil ODS-HG-5, 20×250 mm.

Procedure for the preparation of substrate and physical data

2-(2,6-dioxopiperidin-3-yl)isoindoline-1,3-dione

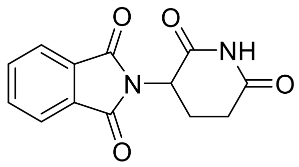

The title substrate was prepared according to the literature[1].

3-(4-amino-1-oxoisoindolin-2-yl)piperidine-2,6-dione

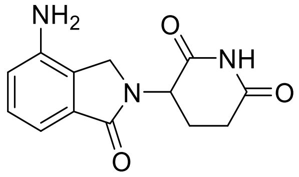

The title substrate was prepared according to the literature[2].

4-amino-2-(2,6-dioxopiperidin-3-yl)isoindoline-1,3-dione

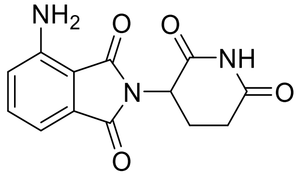

The title substrate was prepared according to the literature[3].

2-(2,6-dioxopiperidin-3-yl)-5-hydroxyisoindoline-1,3-dione

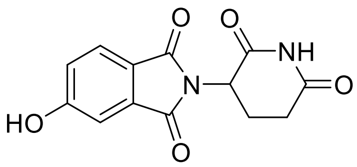

The title substrate was prepared according to the literature[4].

2-(2,6-dioxopiperidin-3-yl)-4-hydroxyisoindoline-1,3-dione

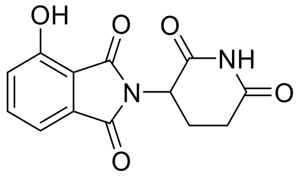

The title substrate was prepared according to the literature[5].

2-(2,6-dioxopiperidin-3-yl)-5-fluoroisoindoline-1,3-dione

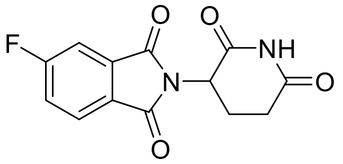

The title substrate was prepared according to the literature[6].

1-Bromo-3,4-difluoro-2-methylbenzene

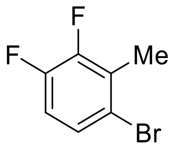

To a solution of 1,2-difluoro-3-methylbenzene (5.0 g, 39 mmol), Fe (218 mg, 3.9 mmol) in $CHCl_3$ (26 mL) was added bromine (2.0 mL, 39 mmol). The resulting mixture was stirred for 12 h at room temperature. The reaction was quenched with water, and $Na_2S_2O_3$ aq. The reaction mixture was filtered through a pad of celite and extracted with CH_2Cl_2. The combined organic layers were washed with brine, dried over Na_2SO_4, filtered and concentrated under reduced pressure to give the title product as a brown oil (7.19 g, 89% yield). **^{1}H-NMR (300 MHz, $CDCl_3$)** δ: 7.30-7.28 (m, 1H), 6.91 (dd, *J* = 17.7, 8.9 Hz, 1H), 2.36 (s, 3H). **^{13}C-NMR (126 MHz, $CDCl_3$)** δ: 150.0 (dd, *J* = 248.4, 14.1 Hz), 149.3 (dd, *J* = 248.9, 13.6 Hz), 128.1 (d, *J* = 15.4 Hz), 127.4 (t, *J* = 5.0 Hz), 119.4, 115.4 (d, *J* = 18.2 Hz), 15.2. **^{19}F-NMR (282 MHz, $CDCl_3$)** δ: -136.1-136.2 (m, 1F), -139.3 (ddd, *J* = 20.6, 9.7, 4.7 Hz, 1F). **ATR-FTIR (KBr)**: *v* = 3079, 2965, 2930, 1852, 1718, 1439, 1381, 1192, 682, 573 cm^{-1}. **MS (ESI)** *m/z*: $[2M+H]^+$ 413.

3,4-Difluoro-2-methylbenzonitrile

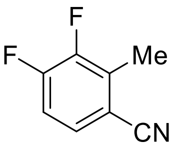

A 300 mL flask was charged with 1-bromo-3,4-difluoro-2-methylbenzene (4.39 g, 21 mmol), CuCN (2.28 mg, 26 mmol), CuI (808 mg, 4.2 mmol) and DMF (23 mL). The mixture was heated to reflux and stirred for 14 h. After cooling to room temperature, the reaction mixture was quenched with water. The mixture was filtered through a pad of celite and washed with Et_2O. The filtrate was washed with water and brine, dried over Na_2SO_4, and concentrated under reduced pressure. The crude product was purified by flash column chromatography (Hexane/EtOAc = 9/1) to give the title product as a white solid (895 mg, 19% yield).

Mp: 33.6-34.1 °C. **^{1}H-NMR (300 MHz, CDCl$_3$)** δ: 7.41 (dd, *J* = 8.1, 4.5 Hz, 1H), 7.12 (dd, *J* = 17.0, 8.2 Hz, 1H), 2.52 (s, 3H). **^{13}C NMR (176 MHz, CDCl$_3$)** δ: 153.4 (dd, *J* = 257.7, 13.4 Hz), 149.3 (dd, *J* = 249.3, 13.4 Hz), 132.3 (d, *J* = 16.7 Hz), 129.1 (dd, *J* = 7.5, 4.2 Hz), 116.5, 115.9 (d, *J* = 18.4 Hz), 110.1 (dd, *J* = 4.1), 13.4. **^{19}F-NMR (282 MHz, CDCl$_3$)** δ: -128.5 (ddd, *J* = 20.8, 9.4, 4.5 Hz, 1F), -137.8 (dd, *J* = 21.1, 6.2 Hz, 1F). **ATR-FTIR (KBr)**: *v* = 3096, 3060, 2309, 2232, 1622, 1498, 1458, 1290, 884, 713 cm^{-1}. **MS (ESI)** *m/z*: [M-H]$^-$ 177.

3,4-Difluorophthalic acid

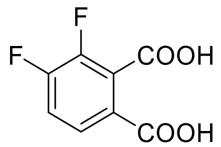

A test tube was charged with 3,4-Difluoro-2-methylbenzonitrile (150 mg, 1.0 mmol), NaOH (118 mg, 2.9 mmol) in water (2 mL). The resulting mixture was heated to reflux and stirred for 1 h. The reaction mixture was cooled to room temperature, added $KMnO_4$ (310 mg, 2.0 mmol), heated to reflux, and stirred for 3 h. The reaction mixture was cooled to room temperature, filtered through a pad of celite and acidified to pH 1-2 with 12 mol/L HCl aq. The resulting precipitate was filtered and dried in a vacuum to give the title product as a white solid (34 mg, 17% yield).

Mp: 144.4-145.2 °C. **^{1}H-NMR (300 MHz, DMSO-*d*$_6$)** δ: 13.78 (brs, 1H), 7.79 (dd, *J* = 7.9, 4.8 Hz, 1H), 7.62 (dd, *J* = 18.1, 8.6 Hz, 1H). **^{13}C-NMR (75 MHz, DMSO-*d*$_6$)** δ: 165.2, 164.4, 149.1 (dd, *J* = 431.2, 251.0 Hz), 149.0 (dd, *J* = 433.2, 251.3 Hz), 127.3 (dd, *J* = 7.2, 3.3 Hz), 127.0, 125.8, 118.0 (d, *J* = 17.7 Hz). **^{19}F-NMR (282 MHz, DMSO-*d*$_6$)** δ: -130.7 (ddd, *J* = 22.5, 10.2, 4.7 Hz, 1F), -141.6 (dd, *J* = 22.8, 7.9 Hz, 1F). **ATR-FTIR (KBr)**: *v* = 3008, 2887, 2667, 1717, 1620, 1597, 1464, 1419, 1293, 1217 cm^{-1}. **HRMS (ESI$^+$)**: *m/z* calcd for C8H3F2O4 [M-H]$^-$: 201.0005 found: 200.9999.

2-(2,6-dioxopiperidin-3-yl)-4,5-difluoroisoindoline-1,3-dione

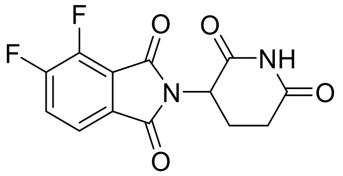

To a solution of 3,4-difluorophthalic acid (455 mg, 2.3 mmol), 2-Aminoglutarimide hydrochloride (445 mg, 2.7 mmol) in AcOH (9.2 mL) was added AcONa (221 mg, 2.7 mmol). The resulting mixture was heated to reflux and stirred for 15 h. The reaction mixture was cooled to room temperature and added water. The resulting precipitate was filtered, washed with water, and dried in a vacuum desiccator to give the title product as a white solid (310 mg, 47% yield).

Mp: 237.7–238.7 °C. **^{1}H-NMR (300 MHz, CDCl$_3$)** δ: 7.99 (s, 1H), 7.71 (dd, *J* = 7.8, 4.0 Hz, 1H), 7.57

(ddd, *J* = 16.2, 8.1, 1.5 Hz, 1H), 5.01-4.95 (m, 1H), 2.97-2.76 (m, 3H), 2.20-2.16 (m, 1H). **^{13}C-NMR (176 MHz, DMSO-*d*$_6$)** δ: 172.7, 169.6, 165.4, 163.0, 153.8 (dd, *J* = 255.1, 10.9 Hz), 145.1 (dd, *J* = 265.2, 15.9 Hz), 128.0, 124.2 (d, *J* = 20.1 Hz), 121.3 (d, *J* = 5.0 Hz), 119.6 (d, *J* = 8.4 Hz), 49.3, 30.9, 21.8. **^{19}F-NMR (282 MHz, CDCl$_3$)** δ: -126.5 (dd, *J* = 21.8, 9.9 Hz, 1F), -136.0 (dd, *J* = 20.8, 5.9 Hz, 1F). **ATR-FTIR (KBr)**: *v* = 3423, 2365, 2339, 1714, 1506, 1395, 1267, 1001, 868, 795 cm^{-1}. **HRMS (ESI$^+$)**: *m/z* calcd for C13H7F2N2O4 [M-H]$^-$: 293.0379 found: 293.0369.

2-(2,6-dioxopiperidin-3-yl)-4,7-difluoroisoindoline-1,3-dione (NE-002)

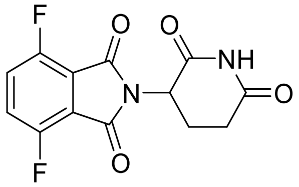

To a solution of 4,7-difluoroisobenzofuran-1,3-dione (221 mg, 1.2 mmol), 2-Aminoglutarimide hydrochloride (217 mg, 1.32 mmol) in AcOH (4.0 mL) was added AcONa (353 mg, 3.6 mmol). The resulting mixture was heated to reflux and stirred for 15 h. The reaction mixture was cooled to room temperature and added water. The resulting precipitate was filtered, washed with water. Then the precipitate was dissolved in THF and filtered. The filtrate was concentrated under reduced pressure and recrystallized with MeOH to give the title product as a white solid (208 mg, 59% yield).

Mp: 254.3-255.1 °C. **^{1}H-NMR (300 MHz, CDCl$_3$)** δ: 7.97 (s, 1H), 7.44 (dd, *J* = 5.4, 5.4 Hz, 2H), 5.03-4.91 (m, 1H), 3.02-2.66 (m, 3H), 2.26-2.09 (m, 1H). **^{13}C-NMR (126 MHz, DMSO-*d*$_6$)** δ: 172.8, 169.6, 163.0, 153.1 (d, *J* = 260.7 Hz), 126.0 (t, *J* = 15.4 Hz), 118.1 (t, *J* = 7.7 Hz), 49.2, 30.9, 21.8. **^{19}F-NMR (282 MHz, CDCl$_3$)** δ: -116.8 (dd, *J* = 5.2, 5.2 Hz, 2F).

ATR-FTIR (KBr): *v* = 3443, 3212, 3086, 2968, 1725, 1494, 1423, 1157, 1037, 691 cm^{-1}. **MS (ESI)** *m/z*: [M-H]$^-$ 293.

5-fluoro-2-methyl-3-nitrobenzoic acid

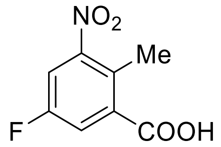

To a solution of 5-Fluoro-2-methylbenzoic acid (5.0 g, 32.4 mmol) in conc. H_2SO_4 (39 mL) was added fuming HNO_3 (6.9 mL) dropwise slowly at 0 °C. The resulting mixture was stirred for 5 h at 0 °C. The reaction mixture was poured into crushed ice, and the resulting precipitate was filtered and washed with water. The precipitate was dried in a vacuum to give the title product as a white solid (3.8 g, 60% yield).

Mp: 161.7-161.9 °C. **^{1}H-NMR (300 MHz, DMSO-*d*$_6$)** δ: 8.06 (dd, *J* = 7.9, 2.6 Hz, 1H), 7.87 (dd, *J* = 8.5, 2.6 Hz, 1H), 2.45 (s, 3H). **^{13}C-NMR (126 MHz, DMSO-*d*$_6$)** δ: 166.7, 159.0 (d, *J* = 247.0 Hz), 151.8 (d, *J*

= 9.1 Hz), 136.3 (d, J = 7.3 Hz), 127.1 (d, J = 4.5 Hz), 120.3 (d, J = 22.7 Hz), 114.0 (d, J = 27.2 Hz), 15.1. **^{19}F-NMR (282 MHz, DMSO-d_6)** δ: -112.85 (t, J = 8.6 Hz, 1F). **ATR-FTIR (KBr)**: v = 3177, 3082, 2669, 1703, 1586, 1530, 1415, 1093, 1032, 693 cm^{-1}. **MS (ESI)** *m/z*: [M-H]$^-$ 198.

5-fluoro-3-nitrophthalic acid

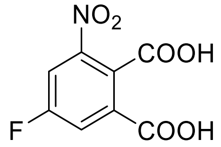

A test tube was charged with 5-fluoro-2-methyl-3-nitrobenzoic acid (500 mg, 2.51 mmol), NaOH (301 mg, 7.53 mmol) in water (5.0 mL). The resulting mixture was heated to reflux and stirred for 1 h. The reaction mixture was cooled to room temperature, added $KMnO_4$ (1.58 g, 10.0 mmol), heated to reflux, and stirred for 4 h. The reaction mixture was cooled to room temperature, filtered through a pad of celite and acidified to pH 1-2 with 12 mol/L HCl aq. The resulting precipitate was filtered and dried in a vacuum to give the title product as a white solid (181 mg, 31% yield).

Mp: 180.7-181.6 °C. **^{1}H-NMR (300 MHz, Acetone-d_6)** δ: 8.22 (dd, J = 8.1, 2.5 Hz, 1H), 8.12 (dd, J = 8.2, 2.6 Hz, 1H), 3.55-2.47 (brs, 2H). **^{13}C-NMR (126 MHz, DMSO-d_6)** δ: 165.4, 164.9, 160.8 (d, J = 251.6 Hz), 147.7 (d, J = 9.1 Hz), 133.8 (d, J = 7.3 Hz), 127.1, 122.1 (d, J = 23.6 Hz), 115.5 (d, J = 27.2 Hz). **^{19}F-NMR (282 MHz, Acetone-d_6)** δ: -108.5 (dd, J = 7.8, 7.8 Hz, 1F). **ATR-FTIR (KBr)**: v = 3073, 2884, 2665, 1839, 1742, 1620, 1547, 1435, 1079, 774 cm^{-1}. **MS (ESI)** *m/z*: [M-H]$^-$ 228.

2-(2,6-dioxopiperidin-3-yl)-6-fluoro-4-nitroisoindoline-1,3-dione

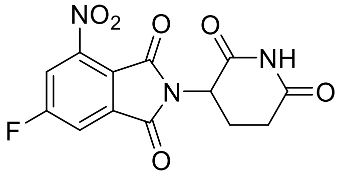

To a solution of 5-fluoro-3-nitrophthalic acid (45.8 mg, 0.20 mmol), 2-Aminoglutarimide hydrochloride (39.5 mg, 0.24 mmol) in AcOH (0.67 mL) was added AcONa (49.2 mg, 0.60 mmol). The resulting mixture was stirred at 90 °C for 6 h. The reaction mixture was cooled to room temperature, and added water and EtOAc. Then the mixture was extracted with EtOAc. The combined organic layers were washed with brine, dried over Na_2SO_4, and concentrated under reduced pressure. The residue was purified by flash column chromatography (Hexane/EtOAc = 1/1) to give the title product as a yellow solid (19.6 mg, 31% yield).

Mp: 226.6-234.9 °C (decomp.). **^{1}H-NMR (300 MHz, DMSO-d_6)** δ: 11.20 (s, 1H), 8.42 (dd, J = 8.4, 1.7 Hz, 1H), 8.27 (dd, J = 6.6, 1.6 Hz, 1H), 5.26-5.16 (m, J = 12.7, 5.2 Hz, 1H), 2.98-2.79 (m, 1H), 2.70-2.52 (m, 2H), 2.14-1.98 (m, 1H).

^{13}C-NMR (126 MHz, DMSO- d_6) δ: 172.8, 169.5, 165.5 (d, J = 259.8 Hz), 164.2, 161.9, 145.5 (d, J = 10.0 Hz), 135.7 (d, J = 10.0 Hz), 118.9, 116.9 (d, J = 29.1 Hz), 115.7 (d, J = 25.4 Hz), 49.6, 30.9, 21.8. **^{19}F-NMR (282 MHz, DMSO- d_6)** δ: -97.59 (dd, J = 7.5, 7.5 Hz, 1F). **ATR-FTIR (KBr)**: v = 3444, 3203, 3097, 2914, 1625, 1555, 1438, 1328, 1029, 873, 657 cm^{-1}. **MS (ESI)** *m/z*: [M-H]$^-$ 320.

4-amino-2-(2,6-dioxopiperidin-3-yl)-6-fluoroisoindoline-1,3-dione (NE-003)

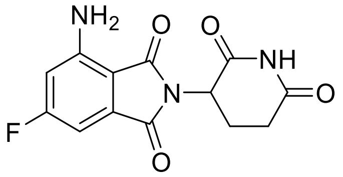

To a solution of 2-(2,6-dioxopiperidin-3-yl)-6-fluoro-4-nitroisoindoline-1,3-dione (64 mg, 0.20 mmol) in THF/DMF = 1/1 (5.0 mL), 10% Pd/C (6 mg, 10 wt %) was added, and the mixture was stirred at room temperature under H_2 (balloon) for 1 h. The mixture was filtered through a pad of celite, and the filtrate was concentrated under reduced pressure. The residue was filtered and washed with Et_2O to give the title product as a yellow solid (43 mg, 74% yield).

Mp: 290.0-300.0 °C (decomp.). **^{1}H-NMR (300 MHz, DMSO-d_6)** δ: 11.10 (s, 1H), 6.84 (dd, J = 6.9, 1.4 Hz, 1H), 6.79-6.68 (m, 3H), 5.08-4.98 (m, 1H), 2.94-2.77 (m, 1H), 2.73-2.51 (m, 2H), 2.06-1.91 (m, 1H). **^{13}C-NMR (126 MHz, DMSO-d_6)** δ: 172.9, 170.1, 167.7, 166.8 (d, J = 251.2 Hz), 166.3, 148.5 (d, J = 13.6 Hz), 134.9 (d, J = 11.8 Hz), 106.2 (d, J = 25.4 Hz), 105.9, 99.6 (d, J = 27.2 Hz), 48.7, 31.0, 22.1. **^{19}F NMR (282 MHz, DMSO-d_6)** δ: -102.53 (dd, J = 11.4, 7.1 Hz, 1F). **ATR-FTIR (KBr)**: v = 3444, 3359, 3224, 3006, 2821, 1646, 1417, 1137, 680, 614 cm^{-1}. **MS (ESI)** *m/z*: [M-H]$^-$ 290.

4-fluoro-7-nitroisobenzofuran-1,3-dione

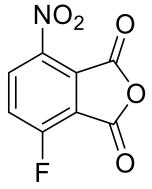

3-fluoro-6-nitrophthalic acid (2.00 g, 8.73 mmol) in acetic anhydride (8.7 mL) was stirred at 100 °C for 1 h. The mixture was cooled to room temperature, and the solvent was removed under reduced pressure to give the title product as a white solid. (1.53 g, 83% yield)

Mp: 147.3-148.1 °C. **^{1}H-NMR (300 MHz, $CDCl_3$)** δ: 8.46 (dd, J = 8.9, 3.6 Hz, 1H), 7.76 (dd, J = 8.8, 7.3 Hz, 1H). **^{13}C-NMR (126 MHz, DMSO-d_6)** δ: 164.6, 163.5, 160.9 (d, J = 259.8 Hz), 143.0, 131.2 (d, J = 3.6 Hz), 128.8 (d, J = 10.9 Hz), 122.5 (d, J = 20.0 Hz), 118.9 (d, J = 24.5 Hz). **^{19}F-NMR (282 MHz, $CDCl_3$)** δ: -100.33 (dd, J = 6.4, 3.3 Hz, 1F).

ATR-FTIR (KBr): v = 3104, 2933, 2873, 2626, 1722, 1617, 1530, 1414, 1174, 773 cm^{-1}. **MS (ESI)** *m/z*:

$[M+H]^+$ 212.

2-(2,6-dioxopiperidin-3-yl)-4-fluoro-7-nitroisoindoline-1,3-dione

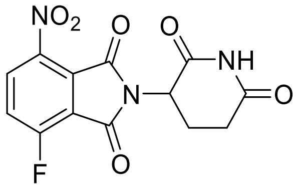

To a solution of 4-fluoro-7-nitroisobenzofuran-1,3-dione (501 mg, 2.37 mmol), 2-Aminoglutarimide hydrochloride (585 mg, 3.55 mmol) in AcOH (9.5 mL) was added AcONa (58.3 mg, 0.71 mmol). The resulting mixture was stirred at 120 °C for 14 h. The reaction mixture was cooled to room temperature, and added water and EtOAc. Then the mixture was extracted with EtOAc. The combined organic layers were washed with brine, dried over Na_2SO_4, and concentrated under reduced pressure. The residue was purified by flash column chromatography to give the title product as a white solid (318 mg, 42% yield).

Mp: 264.5-269.0 °C (decomp.). **^{1}H-NMR (300 MHz, DMSO-d_6)** δ: 11.18 (s, 1H), 8.44 (dd, *J* = 8.9, 3.2 Hz, 1H), 8.04-7.95 (m, 1H), 5.20 (dd, *J* = 12.8, 5.5 Hz, 1H), 2.96-2.79 (m, 1H), 2.68-2.54 (m, 1H), 2.45-2.36 (m, 1H), 2.12-1.97 (m, 1H). **^{13}C-NMR (126 MHz, DMSO-d_6)** δ 172.7, 169.3, 162.1, 161.6, 158.2 (d, *J* = 267.9 Hz), 141.1, 132.5 (d, *J* = 9.1 Hz), 125.3, 124.7 (d, *J* = 21.8 Hz), 118.8 (d, *J* = 14.5 Hz), 49.5, 30.8, 21.6. **^{19}F-NMR (282 MHz, DMSO- d_6)** δ: -108.14 (dd, *J* = 8.0, 2.7 Hz, 1F). **ATR-FTIR (KBr)**: *v* = 3445, 3203, 3091, 2944, 2909, 1611, 1539, 1421, 1063, 752 cm^{-1}. **MS (ESI)** *m/z*: $[M-H]^-$ 320.

4-amino-2-(2,6-dioxopiperidin-3-yl)-7-fluoroisoindoline-1,3-dione (NE-004)

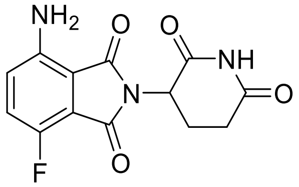

To a solution of 2-(2,6-dioxopiperidin-3-yl)-4-fluoro-7-nitroisoindoline-1,3-dione (100 mg, 0.33 mmol) in THF/DMF = 1/1 (8.2 mL), 10% Pd/C (6 mg, 6 wt %) was added, and the mixture was stirred at rt under H_2 (balloon) for 3 h. The mixture was filtered through a pad of celite, and the solvent was removed under reduced pressure to give the title product as a yellow solid (64 mg, 67% yield).

Mp: 287.3-288.3 °C. **^{1}H-NMR (300 MHz, DMSO-d_6)** δ: 11.12 (s, 1H), 7.37 (dd, *J* = 9.1, 9.1 Hz, 1H), 7.07 (dd, *J* = 9.1, 3.6 Hz, 1H), 6.50 (s, 2H), 5.10-5.01 (m, 1H), 2.95-2.79 (m, 2H), 2.64-2.52 (m, 1H), 2.09-1.94 (m, 1H). **^{13}C-NMR (126 MHz, DMSO-d_6)** δ: 172.8, 170.0, 167.6, 164.1, 148.2 (d, *J* = 251.6 Hz), 143.9, 125.0 (d, *J* = 12.7 Hz), 124.9 (d, *J* = 2.7 Hz), 115.5 (d, *J* = 12.7 Hz), 107.5, 48.6, 31.0, 22.0. **^{19}F-NMR (282 MHz, DMSO- d_6)** δ: -129.27 (dd, *J* = 8.8, 3.0 Hz, 1F).

ATR-FTIR (KBr): *v* = 3482, 3378, 3208, 3094, 2888, 1644, 1599, 1428, 991, 694 cm^{-1}. **MS (ESI)** *m/z*: $[M-H]^-$ 290.

3-(6-fluoro-4-nitro-1-oxoisoindolin-2-yl)piperidine-2,6-dione

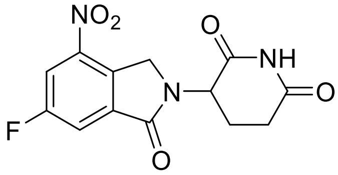

To a solution of methyl 2-(bromomethyl)-5-fluoro-3-nitrobenzoate (438 mg, 1.50 mmol), 2-Aminoglutarimide hydrochloride (247 mg, 1.50 mmol) in DMF (1.5 mL) was added Et_3N (0.25 mL, 1.8 mmol). The resulting mixture was stirred at 50 °C for 12 h. The reaction mixture was cooled to room temperature and added water. The resulting precipitate was filtered and washed with H_2O and MeOH to give the title product as a white solid (150 mg, 33% yield).

Mp: 261.3-272.2 °C (decomp.). **^{1}H-NMR (300 MHz, DMSO-*d_6*)** δ: 11.05 (s, 1H), 8.38 (dd, *J* = 9.1, 1.9 Hz, 1H), 8.11 (dd, *J* = 6.8, 1.7 Hz, 1H), 5.22-5.11 (m, 1H), 4.80 (dd, *J* = 35.2, 18.9 Hz, 2H), 2.99-2.78 (m, 1H), 2.64-2.50 (m, 2H), 2.07-1.90 (m, 1H). **^{13}C-NMR (126 MHz, DMSO-*d_6*)** δ: 172.8, 170.6, 165.1, 161.9 (d, *J* = 249.8 Hz), 144.0 (d, *J* = 8.2 Hz), 136.3 (d, *J* = 8.2 Hz), 133.2, 116.9 (d, *J* = 23.6 Hz), 113.0 (d, *J* = 28.2 Hz), 52.0, 48.3, 31.2, 22.2. **^{19}F-NMR (282 MHz, DMSO-*d_6*)** δ: -109.38 (dd, *J* = 7.7 Hz, 1F). **ATR-FTIR (KBr)**: *ν* = 3436, 3191, 3105, 3040, 2910, 1600, 1546, 1411, 990, 854 cm^{-1}. **MS (ESI)** *m/z*: $[M-H]^-$ 306.

3-(4-amino-6-fluoro-1-oxoisoindolin-2-yl)piperidine-2,6-dione (NE-005)

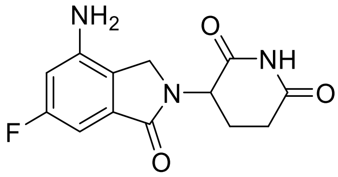

To a solution of 3-(6-fluoro-4-nitro-1-oxoisoindolin-2-yl)piperidine-2,6-dione (100 mg, 0.327 mmol) in 1,4-dioxane (4.1 mL), 10% Pd/C (22.9 mg, 23 wt %) was added, and the mixture was stirred at room temperature under H_2 (balloon) for 3 h. The mixture was filtered through a pad of celite, and the solvent was removed under reduced pressure to give the title product as a white solid (16 mg, 18% yield).

Mp: 263.3-265.8 °C (decomp.). **^{1}H-NMR (300 MHz, DMSO-*d_6*)** δ: 11.03 (s, 1H), 6.69-6.49 (m, 2H), 5.81 (s, 2H), 5.15-5.04 (m, 1H), 4.13 (dd, *J* = 34.3, 17.0 Hz, 2H), 3.00-2.81 (m, 1H), 2.70-2.54 (m, 1H), 2.38-2.19 (m, 1H), 2.11-1.96 (m, 1H). **^{13}C-NMR (126 MHz, DMSO-*d_6*)** δ: 172.93, 171.16, 168.0 (d, *J* = 3.6 Hz), 163.4 (d, *J* = 240.7 Hz), 145.5 (d, *J* = 11.8 Hz), 133.7 (d, *J* = 11.8 Hz), 121.9, 102.6 (d, *J* = 26.3 Hz), 96.2 (d, *J* = 23.6 Hz), 51.7, 45.4, 31.2, 22.7. **^{19}F-NMR (282 MHz, DMSO-*d_6*)** δ: -113.63 (dd, *J* = 11.1, 8.3 Hz, 1F). **ATR-FTIR (KBr)**: *ν* = 3409, 3347, 3237, 3093, 2906, 2877, 1642, 1414, 1032, 882 cm^{-1}. **MS (ESI)** *m/z*: $[M-H]^-$ 276.

6-Fluoro-2-methyl-3-nitrobenzoic acid

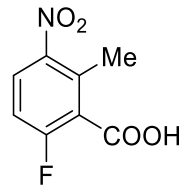

To a solution of 2-fluoro-6-methylbenzoic acid (4.91 g, 32 mmol) in conc. H_2SO_4 (41 mL) was added fuming HNO_3 (1.8 mL) dropwise slowly at 0 °C. The resulting mixture was stirred at 0 °C for 5 h. The reaction mixture was poured into crushed ice, and the resulting precipitate was filtered and washed with water. The precipitate was dried in a vacuum to give the title product as a white solid (3.92 g, 62% yield).
Mp: 136.8-137.6 °C. **^{1}H-NMR (300 MHz, $CDCl_3$)** δ: 11.00 (s, 1H), 8.06 (dd, *J* = 9.1, 5.0 Hz, 1H), 7.18 (t-like, *J* = 8.5 Hz, 1H), 2.66 (s, 3H). **^{13}C-NMR (126 MHz, DMSO-*d_6*)** δ: 165.0, 159.7 (d, *J* = 254.3 Hz), 146.3, 132.2, 127.7 (d, *J* = 10.9 Hz), 125.9 (d, *J* = 20.0 Hz), 115.0 (d, *J* = 24.5 Hz), 16.3. **^{19}F-NMR (282 MHz, $CDCl_3$)** δ: -104.9 (dd, *J* = 7.9, 5.0 Hz, 1F). **ATR-FTIR (KBr)**: *ν* = 3103, 3012, 2881, 2661, 2550, 1709, 1526, 1431, 1144, 700 cm^{-1}. **MS (ESI)** *m/z*: [M-H]$^-$ 198.

Methyl 6-fluoro-2-methyl-3-nitrobenzoate

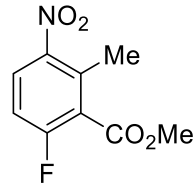

To a solution of 6-fluoro-2-methyl-3-nitrobenzoic acid (300 mg,1.5 mmol) in DMF (3.0 mL) was added CH_3I (0.12 mL, 2.0 mmol) and K_2CO_3 (416 mg, 3.0 mmol). The resulting mixture was stirred for 14 h at room temperature. The reaction mixture was added into water. The mixture was extracted with EtOAc three times, and combined organic layers were washed with brine, dried over Na_2SO_4, and concentrated under reduced pressure to give the title product as a white solid (297 mg, 91% yield).
Mp: 70.9-71.5 °C. **^{1}H-NMR (300 MHz, $CDCl_3$)** δ: 8.01 (dd, *J* = 9.1, 5.3 Hz, 1H), 7.12 (t-like, *J* = 8.5 Hz, 1H), 3.99 (s, 3H), 2.54 (s, 3H). **^{13}C-NMR (126 MHz, $CDCl_3$)** δ: 164.5, 161.2 (d, *J* = 259.3 Hz), 146.4, 134.4 (d, *J* = 4.5 Hz), 128.1 (d, *J* = 10.0 Hz), 124.9 (d, *J* = 19.1 Hz), 114.6 (d, *J* = 23.6 Hz), 53.3, 17.1. **^{19}F-NMR (282 MHz, $CDCl_3$)** δ: -106.3 (dd, *J* = 7.9, 5.0 Hz, 1F). **ATR-FTIR (KBr)**: *ν* = 3104, 2960, 1739, 1620, 1533, 1440, 1355, 1265, 1004, 829 cm^{-1}. **MS (ESI)** *m/z*: [M-H]$^-$ 212.

Methyl 2-(bromomethyl)-6-fluoro-3-nitrobenzoate

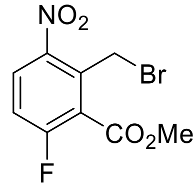

To a solution of methyl methyl 6-fluoro-2-methyl-3-nitrobenzoate (525 mg, 2.4 mmol), *N*-Bromosuccinimide (418 mg, 2.4 mmol) in degassed CCl_4 (12 mL) was added Benzoyl peroxide (76 mg, 0.24 mmol). The resulting mixture was heated to reflux and stirred for 31 h. The reaction mixture was cooled to room temperature, filtered, and concentrated under reduced pressure. The resulting mixture was purified by flash column chromatography (Hexane/EtOAc = 95/5 to 9/1) to give the title product as a pale yellow solid (265 mg, 37% yield).

Mp: 70.3-71.0 °C. **^{1}H-NMR (300 MHz, $CDCl_3$)** δ: 8.09 (dd, *J* = 9.0, 4.9 Hz, 1H), 7.26 (t like, *J* = 8.4 Hz, 2H), 4.87 (s, 2H), 4.04 (s, 3H). **^{13}C-NMR (176 MHz, $CDCl_3$)** δ: 163.6, 161.6 (d, *J* = 261.0 Hz), 145.2, 133.9 (d, *J* = 3.3 Hz), 129.1 (d, *J* = 11.7 Hz), 124.3 (d, *J* = 20.1 Hz), 117.3 (d, *J* = 25.1 Hz), 53.7, 22.8. **^{19}F-NMR (282 MHz, $CDCl_3$)** δ: -103.6 (t like, *J* = 6.0 Hz, 1F). **ATR-FTIR (KBr)**: *ν* = 3086, 2962, 1736, 1581, 1540, 1433, 1361, 1270, 841, 635 cm^{-1}. **MS (ESI)** *m/z*: $[M-H]^-$ 292.

3-(7-Fluoro-4-nitro-1-oxoisoindolin-2-yl)piperidine-2,6-dione

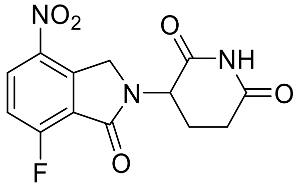

To a solution of methyl 2-(bromomethyl)-6-fluoro-3-nitrobenzoate (100 mg, 0.34 mmol), 2-Aminoglutarimide hydrochloride (62 mg, 0.38 mmol) in DMF (0.3 mL) was added Et_3N (57 µL, 0.4 mmol). The resulting mixture was heated to 70 °C and stirred for 23 h. The reaction mixture was cooled to room temperature and added water. The resulting precipitate was filtered and dried in a vacuum desiccator to give the title product as a yellow solid (13 mg, 12% yield).

Mp: 255.3-256.0 °C. **^{1}H-NMR (300 MHz, DMSO-*d*$_6$)** δ: 11.06 (s, 1H), 8.53 (dd, *J* = 9.0, 3.4 Hz, 1H), 7.63 (t like, *J* = 8.8 Hz, 1H), 5.17-5.11 (m, 1H), 4.85 (dd, *J* = 35.0, 19.7 Hz, 2H), 2.97-2.85 (m, 1H), 2.62-2.54 (m, 2H), 2.02-1.99 (m, 1H). **^{13}C-NMR (176 MHz, DMSO-*d*$_6$)** δ: 172.9, 170.6, 163.1, 161.2 (d, *J* = 267.7 Hz), 141.5 (d, *J* = 3.3 Hz), 139.8, 130.5 (d, *J* = 21.8 Hz), 120.9 (d, *J* = 15.1 Hz), 117.5 (d, *J* = 13.4 Hz), 51.7, 48.6, 31.1, 22.1. **^{19}F-NMR (282 MHz, DMSO-*d*$_6$)** δ: -108.6 (dd, *J* = 8.4, 3.5 Hz, 1F). **ATR-FTIR (KBr)**: *ν* = 3393, 1731, 1702, 1649, 1530, 1451, 1348, 1255, 1233, 748 cm^{-1}. **HRMS (ESI^+)**: *m/z* calcd for C13H9FN3O5 $[M-H]^-$: 306.0532 found: 306.0528.

3-(4-amino-7-fluoro-1-oxoisoindolin-2-yl)piperidine-2,6-dione (NE-006)

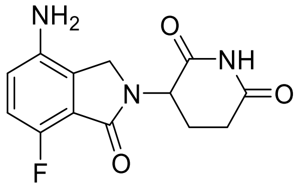

A 10 mL flask was charged with 3-(7-fluoro-4-nitro-1-oxoisoindolin-2-yl)piperidine-2,6-dione (45 mg,

0.15 mmol) , Pd/C (4.5 mg, 10 wt %) in THF (1.8 mL), DMF (1.8 mL). The resulting mixture was stirred under hydrogen for 3 h at room temperature. The reaction mixture was filtered through a pad of celite, and the filtrate was concentrated, filtered, and washed with Et_2O to give the title product as a brown solid (36 mg, 88% yield).

Mp: 234.4-235.1 °C. **^{1}H-NMR (300 MHz, DMSO-*d*$_6$)** δ: 11.03 (s, 1H), 6.98 (t, *J* = 8.9 Hz, 1H), 6.78 (dd, *J* = 8.5, 3.5 Hz, 1H), 5.28 (s, 2H), 5.11-5.04 (m, 1H), 4.15 (dd, *J* = 35.0, 17.4 Hz, 2H), 2.93-2.86 (m, 1H), 2.63-2.57 (m, 1H), 2.31-2.26 (m, 1H), 2.06-2.00 (m, 1H). **^{13}C NMR (126 MHz, DMSO-*d*$_6$)** δ: 172.9, 171.1, 165.9, 149.9 (d, *J* = 244.3 Hz), 140.0, 126.8, 118.4 (d, *J* = 14.5 Hz), 117.8 (d, *J* = 5.4 Hz), 115.5 (d, *J* = 20.9 Hz), 51.4, 45.5, 31.2, 22.6. **^{19}F-NMR (282 MHz, DMSO-*d*$_6$)** δ: -136.1 (dd, *J* = 9.4, 3.5 Hz, 1F). **ATR-FTIR (KBr)**: *v* = 3339, 3103, 2914, 1703, 1682, 1498, 1342, 1254, 1196, 672 cm^{-1}. **HRMS (ESI$^+$)**: *m/z* calcd for C13H11FN3O3 $[M-H]^-$: 276.0790 found: 276.0787.

1-Bromo-4-fluoro-2-methyl-3-nitrobenzene

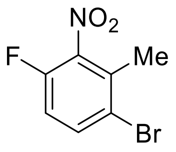

A 50 mL flask was charged with 1-fluoro-3-methyl-2-nitrobenzene (3.03 g, 19.3 mmol) in conc. H_2SO_4 (12.5 mL) and Trifluoroacetic acid (12.5 mL) was added at 0 °C. *N*-Bromosuccinimide (3.42 mg, 29.0 mmol) was added in 4-5 portions for 1 h. Then the mixture was stirred at room temperature for 3 h. After completion of the reaction, ice-cold water was added to the mixture, and the resulting precipitate was filtered. Then the precipitate was dissolved in Et_2O and washed with water and brine. The organic layer was dried over Na_2SO_4, filtered, and concentrated under reduced pressure to give the title product as a white solid (4.17 g, 81% yield).

Mp: 69.2-69.7 °C. **^{1}H-NMR (300 MHz, $CDCl_3$)** δ: 7.68 (t-like, *J* = 6.7 Hz, 1H), 7.03 (t-like, *J* = 8.2 Hz, 1H), 2.42 (s, 4H). **^{13}C-NMR (126 MHz, $CDCl_3$)** δ: 152.9 (d, *J* = 257.0 Hz), 141.1 (d, *J* = 7.3 Hz), 135.3 (d, *J* = 7.3 Hz), 132.6, 120.4 (d, *J* = 3.6 Hz), 115.8 (d, *J* = 20.0 Hz), 18.7. **^{19}F-NMR (282 MHz, $CDCl_3$)** δ: -125.1 (t-like, *J* = 5.9 Hz, 1F). **ATR-FTIR (KBr)**: *v* = 3290, 2888, 1530, 1458, 1367, 1200, 1031, 905, 860, 628 cm^{-1}. **MS (ESI)** *m/z*: $[M-H]^-$ 233.

4-Fluoro-2-methyl-3-nitrobenzonitrile

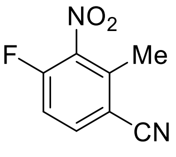

A test tube was charged with 1-bromo-4-fluoro-2-methyl-3-nitrobenzene (130 mg, 0.55 mmol), CuCN (55

mg, 0.61 mmol) and *N*-methyl pyrrolidone (0.3 mL). The mixture was heated to 160 °C and stirred for 12 h. After cooling to room temperature, the reaction mixture was poured into ammonium hydroxide (28%) slowly, diluted with Et_2O, and filtered through a pad of celite. The filtrate was washed with water and brine, dried over Na_2SO_4, and concentrated under reduced pressure. The crude product was purified by flash column chromatography (Hexane/EtOAc = 9/1) to give the title product as a pale yellow solid (70 mg, 70% yield).

Mp: 58.7-59.4 °C. **^{1}H-NMR (300 MHz, $CDCl_3$)** δ: 7.79 (dd, *J* = 8.8, 5.0 Hz, 1H), 7.27 (t-like, J = 8.6 Hz, 1H), 2.61 (s, 3H). **^{13}C-NMR (176 MHz, $CDCl_3$)** δ: 156.0 (d, *J* = 266.0 Hz), 140.8 (d, *J* = 15.1 Hz), 137.7, 136.4 (d, *J* = 8.4 Hz), 116.2 (d, *J* = 21.8 Hz), 115.7, 111.2, 16.6. **^{19}F-NMR (282 MHz, $CDCl_3$)** δ: -113.8 (dd, *J* = 8.9, 5.0 Hz, 1F). **ATR-FTIR (KBr)**: *ν* = 3090, 3063, 2230, 1548, 1486, 1277, 1007, 847, 780, 684 cm^{-1}. **MS (ESI)** *m/z*: $[M-H]^-$ 179.

4-Fluoro-2-methyl-3-nitrobenzoic acid

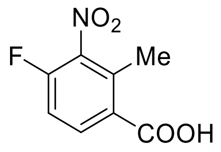

A 50 mL flask was charged with 4-fluoro-2-methyl-3-nitrobenzonitrile (500 mg, 2.8 mmol) in conc. H_2SO_4 (6.0 mL) and water (4.4 mL) was heated to reflux for 1 h. Afterward, $NaNO_2$ (383 mg, 5.6 mmol) was added in 4–5 portions and the mixture was stirred for 2 h. The reaction mixture was cooled to room temperature and basified to pH 14 with 1 mol/L NaOH aq. Then the aqueous layer was extracted with EtOAc. The aqueous layer was cooled to 0 °C and 1 mol/L HCl aq. was added. The resulting precipitate was filtered, washed with water, and dried in a vacuum desiccator to give the title product as a white solid (449 mg, 90% yield).

Mp: 198.8-199.5 °C. **^{1}H-NMR (300 MHz, $CDCl_3$)** δ: 8.23 (dd, *J* = 8.5, 5.6 Hz, 1H), 7.22-7.19 (m, 1H), 2.63 (s, 3H). **^{13}C-NMR (176 MHz, DMSO-d_6)** δ: 166.5, 154.1 (d, *J* = 259.3 Hz), 140.6 (d, *J* = 15.1 Hz), 134.5 (d, *J* = 10.0 Hz), 133.4, 129.1, 114.8 (d, *J* = 18.4 Hz), 15.0. **^{19}F-NMR (282 MHz, $CDCl_3$)** δ: -116.4 (t, *J* = 6.9 Hz, 1F). **ATR-FTIR (KBr)**: *ν* = 3098, 2971, 2647, 1585, 1541, 1371. 1268. 1006, 850, 635 cm^{-1}. **HRMS (ESI$^+$)**: *m/z* calcd for C8H5FNO4 $[M-H]^-$: 198.0208 found:198.0209.

Methyl 4-fluoro-2-methyl-3-nitrobenzoate

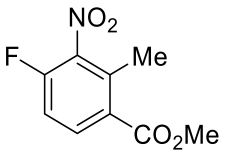

To a solution of 4-fluoro-2-methyl-3-nitrobenzoic acid (982 mg, 4.9 mmol) in MeOH (5.0 mL) was added

$SOCl_2$ (1.1 mL, 15 mmol) dropwise slowly. The resulting mixture was heated to reflux and stirred for 12 h. The reaction mixture was cooled to room temperature and added water. The resulting precipitate was filtered and dried in a vacuum desiccator to give the title product as a white solid (960 mg, 91% yield).

Mp: 74.7-75.5 °C. **^{1}H-NMR (300 MHz, $CDCl_3$)** δ: 8.07 (dd, *J* = 8.8, 5.6 Hz, 1H), 7.17 (t-like, *J* = 8.7 Hz, 1H), 3.93 (s, 3H), 2.57 (s, 3H). **^{13}C-NMR (176 MHz, $CDCl_3$)** δ: 165.7, 156.1, 154.6, 141.8 (d, *J* = 15.1 Hz), 135.0, 134.1 (d, *J* = 10.0 Hz), 127.6, 114.3 (d, *J* = 18.4 Hz), 52.7, 15.6. **^{19}F-NMR (282 MHz, $CDCl_3$)** δ: -118.4 (t-like, *J* = 6.9 Hz, 1F). **ATR-FTIR (KBr)**: *ν* = 3087, 2956, 2362, 1724, 1535, 1435, 1368, 1264, 1128, 779 cm^{-1}. **MS (ESI)** *m/z*: $[M-H]^-$ 212.

Methyl 2-(bromomethyl)-4-fluoro-3-nitrobenzoate

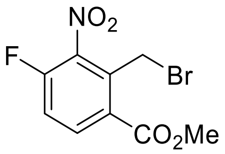

To a solution of methyl 4-fluoro-2-methyl-3-nitrobenzoate (902 mg, 4.6 mmol), *N*-Bromosuccinimide (985 mg, 5.5 mmol) in degassed CCl_4 (23 mL) was added Benzoyl peroxide (112 mg, 0.5 mmol). The resulting mixture was heated to reflux and stirred for 18 h. The reaction mixture was cooled to room temperature, filtered, and concentrated under reduced pressure. The resulting precipitate was purified by flash column chromatography (Hexane/EtOAc = 95/5 to 9/1) to give the title product as a pale yellow solid (659 mg, 49% yield).

Mp: 73.6-74.6 °C. **^{1}H-NMR (300 MHz, $CDCl_3$)** δ: 8.16 (dd, *J* = 8.9, 5.4 Hz, 1H), 7.34-7.26 (m, 1H), 4.95 (s, 2H), 3.99 (s, 3H). **^{13}C-NMR (176 MHz, $CDCl_3$)** δ: 165.7, 155.4 (d, *J* = 262.7 Hz), 141.7 (d, *J* = 15.1 Hz), 135.0, 134.1 (d, *J* = 5.0 Hz), 127.5 (d, *J* = 3.3 Hz), 114.3 (d, *J* = 41.8 Hz), 52.7, 15.6. **^{19}F-NMR (282 MHz, $CDCl_3$)** δ: -116.1 (dd, *J* = 7.9, 5.9 Hz, 1F). **ATR-FTIR (KBr)**: *ν* = 3087, 2957, 1724, 1591, 1537, 1434, 1264, 1217, 1128, 779 cm^{-1}. **MS (ESI)** *m/z*: $[M-H]^-$ 292.

3-(5-Fluoro-4-nitro-1-oxoisoindolin-2-yl)piperidine-2,6-dione

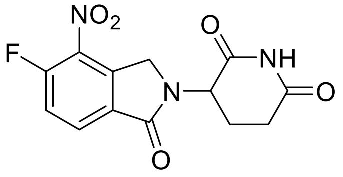

To a solution of methyl 2-(bromomethyl)-4-fluoro-3-nitrobenzoate (659 mg, 2.3 mmol), 2-Aminoglutarimide hydrochloride (446 mg, 2.7 mmol) in DMF (2.3 mL) was added Et_3N (0.4 mL, 3.2 mmol). The resulting mixture was heated to 50 °C and stirred for 2 h. The reaction mixture was cooled to room temperature and added water. The resulting precipitate was filtered and dried in a vacuum desiccator to give the title product as a yellow solid (288 mg, 49% yield).

Mp: 238.8-239.6 °C. **^{1}H-NMR (300 MHz, DMSO-*d*$_6$)** δ: 11.03 (s, 1H), 8.16 (dd, *J* = 8.2, 3.8 Hz, 1H), 7.77 (dd, *J* = 11.4, 8.5 Hz, 1H), 5.18-5.12 (m, 1H), 4.80 (dd, *J* = 33.3, 19.2 Hz, 2H), 2.91-2.84 (m, 1H), 2.62-2.56 (m, 2H), 2.02-2.00 (m, 1H). **^{13}C-NMR (176 MHz, DMSO-*d*$_6$)** δ: 172.8, 170.7, 165.3, 157.3 (d, *J* = 266.0 Hz), 140.1, 133.0 (d, *J* = 8.4 Hz), 130.6 (d, *J* = 11.7 Hz), 129.7, 119.4 (d, *J* = 23.4 Hz), 51.9, 48.0, 31.1, 22.2. **^{19}F-NMR (282 MHz, DMSO-*d*$_6$)** δ: -113.2 (dd, *J* = 11.4, 3.5 Hz, 1F). **ATR-FTIR (KBr)**: *v* = 3095, 2364, 1707, 1671, 1543, 1341, 1237, 1184, 671, 579 cm^{-1}. **HRMS (ESI$^+$)**: *m/z* calcd for C13H9FN3O5 [M-H]$^-$: 306.0532 found: 306.0525.

3-(4-amino-5-fluoro-1-oxoisoindolin-2-yl)piperidine-2,6-dione (NE-008)

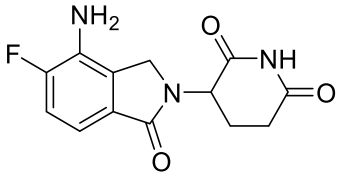

A 10 mL flask was charged with 3-(5-fluoro-4-nitro-1-oxoisoindolin-2-yl)piperidine-2,6-dione (18 mg, 0.057 mmol), Pd/C (2 mg, 10 wt %) in THF (1.2 mL), MeOH (1.2mL). The resulting mixture was stirred under hydrogen for 2 h at room temperature. The reaction mixture was filtered through a pad of celite, and the filtrate was concentrated. The resulting precipitate was filtered and washed with Et_2O to give the title product as a white solid (15 g, 95% yield).

Mp: 219.2-219.9 °C. **^{1}H-NMR (300 MHz, DMSO-*d*$_6$)** δ: 10.99 (s, 1H), 7.12 (dd, *J* = 11.7, 8.2 Hz, 1H), 6.88 (dd, *J* = 7.9, 4.1 Hz, 1H), 5.50 (s, 2H), 5.08-5.02 (m, 1H), 4.16 (dd, *J* = 34.9, 17.3 Hz, 2H), 2.96-2.59 (m, 2H), 2.32- 2.17 (m, 1H), 2.00-1.96 (m, 1H). **^{13}C-NMR (75 MHz, DMSO-*d*$_6$)** δ: 173.0, 171.3, 168.1, 152.1 (d, *J* = 240.5 Hz), 131.8 (d, *J* = 16.0 Hz), 128.2 (d, *J* = 7.2 Hz), 128.0, 115.3 (d, *J* = 21.0 Hz), 110.5 (d, *J* = 7.7 Hz), 51.6, 45.5, 31.3., 22.7. **^{19}F-NMR (282 MHz, DMSO-*d*$_6$)** δ: -131.4 (dd, *J* = 12.4, 4.5 Hz, 1F). **ATR-FTIR (KBr)**: *v* = 3436, 3344, 3222, 1701, 1677, 1505, 1231, 1206, 797, 670 cm^{-1}. **HRMS (ESI$^+$)**: *m/z* calcd for C13H11FN3O3 [M-H]$^-$: 276.0790 found: 276.0790.

methyl 2-methyl-3-nitro-5-(4,4,5,5-tetramethyl-1,3,2-dioxaborolan-2-yl)benzoate

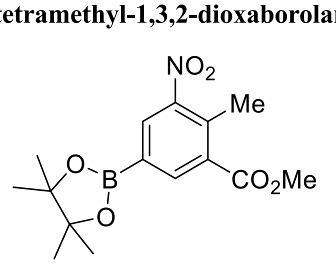

A mixture of methyl 5-bromo-2-methyl-3-nitrobenzoate (500 mg, 1.82 mmol), bis(pinacolato)diboron (462 mg, 1.82 mmol), KOAc (357 mg, 3.64 mmol), and Pd(dppt)Cl_2 (22 mg, 1.5 mol %) in dioxane (4.5 mL) was refluxed at 110 °C for 19 h. The reaction mixture was cooled to room temperature and concentrated. The residue was purified by flash column chromatography (Hexane/CH_2Cl_2 = 1/2) to give the title product

as a white solid (378 mg, 66% yield).

Mp: 114.6-115.3 °C. **^{1}H-NMR (300 MHz, CDCl$_3$)** δ: 8.35 (s, 1H), 8.22 (s, 1H), 3.93 (s, 3H), 2.63 (s, 3H), 1.35 (s, 12H).

^{13}C-NMR (126 MHz, CDCl$_3$) δ: 167.1, 151.9, 139.4, 135.7, 133.0, 132.5, 84.9, 52.7, 25.0, 16.5 (1 carbon was not detected). **ATR-FTIR (KBr)**: *ν* = 2990, 2952, 1729, 1614, 1532, 1439, 1284, 1088, 969, 780 cm^{-1}. **MS (ESI)** *m/z*: [M+H]$^+$ 322.

methyl 5-hydroxy-2-methyl-3-nitrobenzoate

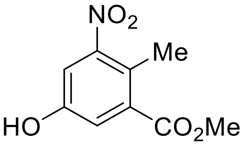

To a solution of methyl 2-methyl-3-nitro-5-(4,4,5,5-tetramethyl-1,3,2-dioxaborolan-2-yl)benzoate (200 mg, 0.62 mmol) in acetone (2.0 mL) was added sat. aq. OXONE® (0.89 mL) at 0 °C. The mixture was stirred at room temperature for 1 h. The reaction was then quenched with sat. aq. $NaHSO_3$. The mixture was concentrated under reduced pressure and extracted with EtOAc. The combined organic layers were washed with brine, dried over Na_2SO_4, concentrated, and purified by flash column chromatography (CH_2Cl_2/MeOH = 30/1) to give the title product as a white solid (123 mg, 94% yield).

Mp: 113.8-114.2 °C. **^{1}H-NMR (300 MHz, CDCl$_3$)** δ: 7.50 (d, *J* = 2.4 Hz, 1H), 7.37 (d, *J* = 2.4 Hz, 1H), 5.43 (s, 1H), 3.93 (s, 3H), 2.52 (s, 3H). **^{13}C-NMR (126 MHz, CDCl$_3$)** δ: 167.1, 153.6, 152.4, 134.3, 124.9, 121.2, 114.2, 52.9, 15.7. **ATR-FTIR (KBr)**: *ν* = 3367, 3311, 1695, 1620, 1574, 1533, 1441, 1097, 997, 882 cm^{-1}. **MS (ESI)** *m/z*: [M-H]$^-$ 210.

methyl 5-((tert-butyldimethylsilyl)oxy)-2-methyl-3-nitrobenzoate

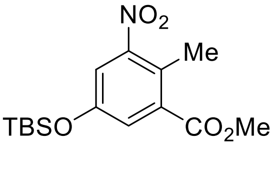

To a solution of methyl 5-hydroxy-2-methyl-3-nitrobenzoate (1.06 g, 5.0 mmol) in CH_3CN (10 mL), *tert*-Butyl[(1-methoxyvinyl)oxy]dimethylsilane (2.6 mL, 12.0 mmol) was added at room temperature. The mixture was stirred at room temperature for 15 h, and the solvent was removed under reduced pressure. The residue was purified by flash column chromatography (Hexane/EtOAc = 9/1) to give the title product as a yellow oil (1.63 g, quant.).

^{1}H-NMR (300 MHz, CDCl$_3$) δ: 7.45 (d, *J* = 2.6 Hz, 1H), 7.32 (d, *J* = 2.6 Hz, 1H), 3.93 (s, 3H), 2.52 (s, 3H), 0.99 (s, 9H), 0.23 (s, 6H). **^{13}C-NMR (126 MHz, CDCl$_3$)** δ: 166.7, 153.5, 152.2, 134.0, 125.5, 125.2, 118.2, 52.6, 25.5, 18.1, 15.5, -4.5. **ATR-FTIR (NaCl)**: ν = 2954, 2860, 1734, 1618, 1535, 1475, 1437, 1362,

1309, 1232, 1093, 1012, 883, 843 cm^{-1}. **HRMS (EI)** *m/z*: $[M]^+$ Calcd. for $C_{15}H_{23}NO_5Si$ 325.1346; found 325.1361.

methyl 2-(bromomethyl)-5-((tert-butyldimethylsilyl)oxy)-3-nitrobenzoate

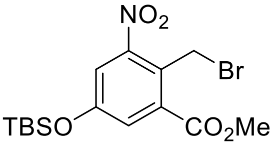

To a solution of methyl 5-((tert-butyldimethylsilyl)oxy)-2-methyl-3-nitrobenzoate (1.03 g, 3.16 mmol), *N*-Bromosuccinimide (676 mg, 3.8 mmol) in degassed CCl_4 (30 mL) was added 2,2'-Azobis(isobutyronitrile) (52.5 mg, 0.32 mmol). The resulting mixture was heated to reflux and stirred for 15 h. The reaction mixture was cooled to room temperature, filtered, and concentrated under reduced pressure. The residue was purified by flash column chromatography (Hexane/Acetone = 96/4) to give the title product as a pale yellow oil (1.12 g, 88% yield).

^{1}H-NMR (300 MHz, $CDCl_3$) δ: 7.53 (d, *J*= 2.6 Hz, 1H), 7.38 (d, *J* = 2.6 Hz, 1H), 5.09 (s, 2H), 3.99 (s, 3H), 1.00 (s, 9H), 0.27 (s, 6H). **^{13}C-NMR (126 MHz, $CDCl_3$)** δ: 165.8, 155.8, 151.3, 133.4, 126.4, 124.8, 119.1, 53.1, 25.4, 23.3, 18.1, -4.5. **ATR-FTIR (NaCl)**: ν = 2954, 2860, 1730, 1612, 1539, 1475, 1437, 1360, 1313, 1240, 1093, 1014, 843, 787 cm^{-1}. **HRMS (EI)** *m/z*: $[M-Br]^+$ Calcd. for $C_{15}H_{22}NO_5Si$ 324.1267; found 324.1262.

3-(6-((tert-butyldimethylsilyl)oxy)-4-nitro-1-oxoisoindolin-2-yl)piperidine-2,6-dione

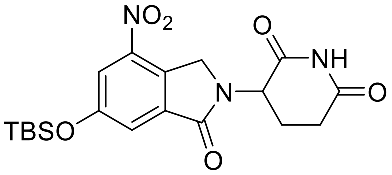

To a solution of methyl 2-(bromomethyl)-5-((tert-butyldimethylsilyl)oxy)-3-nitrobenzoate (1.55 g, 3.80 mmol), 2-Aminoglutarimide hydrochloride (625 mg, 3.80 mmol) in DMF (3.8 mL) was added Et_3N (0.64 mL, 4.6 mmol). The resulting mixture was stirred at 50 °C for 15 h. The mixture was cooled to room temperature and added water. The resulting precipitate was filtered and washed with Et_2O and EtOH to give the title product as a white solid (520 mg, 33% yield).

Mp: 212.8-219.6 °C. **^{1}H-NMR (300 MHz, $CDCl_3$)** δ: 7.96 (brs, 1H), 7.87 (d, *J* = 2.1 Hz, 1H), 7.65 (d, *J* = 2.4 Hz, 1H), 5.22 (dd, *J* = 13.5, 5.3 Hz, 1H), 4.93-4.70 (m, 2H), 3.05-2.92 (m, 1H), 2.92-2.76 (m, 1H), 2.48 (qd, *J* = 13.1, 5.0 Hz, 1H), 2.34-2.19 (m, 1H), 1.02 (s, 9H), 0.28 (s, 6H). **^{13}C-NMR (126 MHz, $CDCl_3$)** δ: 170.7, 169.0, 166.8, 157.3, 143.8, 135.6, 129.1, 121.5, 119.2, 67.1, 52.1, 48.0, 31.5, 25.5, 23.2, 18.2, -4.5. **ATR-FTIR (KBr)**: ν = 3095, 2935, 2860, 1716, 1682, 1579, 1533, 1452, 1352, 1319, 1290, 1254, 1225, 1194, 1122, 980, 935, 893, 845, 812, 787 cm^{-1}. **HRMS (ESI)** *m/z*: $[M+Na]^+$ Calcd. For $C_{19}H_{25}N_3NaO_6Si$

442.1410; found 442.1408.

3-(6-hydroxy-4-nitro-1-oxoisoindolin-2-yl)piperidine-2,6-dione

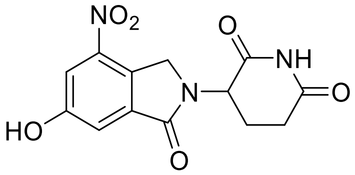

To a solution of 3-(6-((tert-butyldimethylsilyl)oxy)-4-nitro-1-oxoisoindolin-2-yl)piperidine-2,6-dione (520 mg, 1.24 mmol) in 1,4-dioxane (20 mL) was added conc. H_2SO_4 (0.5 mL) slowly at room temperature. The mixture was stirred at room temperature for 15 h, and the solvent was removed under reduced pressure. The residue was recrystallized with EtOH to give the title product as a off-white solid (330 mg, 87% yield). **Mp**: 249.6-279.7 °C (decomp.). **^{1}H-NMR (300 MHz, DMSO-*d6*)** δ: 11.02 (s, 1H), 10.87 (s, 1H), 7.78 (d, *J* = 2.4 Hz, 1H), 7.47 (d, *J* = 2.1 Hz, 1H), 5.14 (dd, *J* = 13.1, 4.8 Hz, 1H), 4.83-4.57 (m, 2H), 2.99-2.80 (m, 1H), 2.68-2.53 (m,2H), 2.10- 1.91 (m, 1H). **^{13}C-NMR (126 MHz, DMSO-*d6*)** δ: 172.9, 170.8, 166.0, 158.7, 143.7, 135.7, 127.4, 115.8, 113.7, 51.9, 48.0, 31.2, 22.3. **ATR-FTIR (KBr)**: ν = 3195, 1678, 1533, 1469, 1352, 1292, 1224, 1201, 1122, 964, 887, 796, 756 cm^{-1}. **HRMS (ESI)** *m/z*: $[M+Na]^+$ Calcd. for $C_{13}H_{11}N_3NaO_6$ 328.0546; found 328.0548.

3-(4-amino-6-hydroxy-1-oxoisoindolin-2-yl)piperidine-2,6-dione (NE-009)

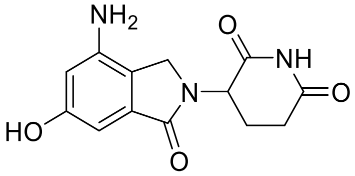

To a solution of 3-(6-hydroxy-4-nitro-1-oxoisoindolin-2-yl)piperidine-2,6-dione (200 mg, 0.66 mmol) in THF (5.0 mL), 10% Pd/C (70 mg, 35 wt %) was added, and the mixture was stirred at room temperature under H_2 (balloon) for 5 h. The mixture was filtered through a pad of celite, and the solvent was removed under reduced pressure. The residue was recrystallized with EtOH to give the title product as an off-white solid (39.9 mg, 22% yield).

Mp: 178.2-197.7 °C (decomp.). **^{1}H-NMR (300 MHz, Acetone-*d6*)** δ: 9.76 (brs, 1H), 8.34 (s, 1H), 6.55 (d, *J* = 2.1 Hz, 1H), 6.42 (d, *J* = 2.1 Hz, 1H), 5.15 (dd, *J* = 13.2, 5.0 Hz, 1H), 4.89 (brs, 1H), 4.21 (s, 2H), 3.06-2.91 (m, 1H), 2.81-2.69 (m, 1H), 2.57-2.37 (m, 1H), 2.26-2.12 (m, 1H). **^{13}C-NMR (126 MHz, Acetone-*d6*)** δ: 172.9, 171.6, 170.0, 159.7, 145.2, 134.8, 119.2, 105.3, 99.2, 52.8, 45.7, 32.3, 24.2. **ATR-FTIR (KBr)**: ν = 3359, 3219, 1682, 1500, 1454, 1419, 1369, 1315, 1211, 1146, 1005, 835, 769, 744 cm^{-1}. **HRMS (ESI)** *m/z*: $[M+Na]^+$ Calcd. for $C_{13}H_{13}N_3NaO_4$ 298.0804; Found 298.0801.

6-Bromo-2-fluoro-3-methoxy benzoic acid

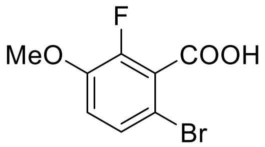

To a solution of 2-fluoro-3-methoxy benzoic acid (4.93 g, 29.0 mmol) in AcOH (24.0 mL) and water (24.0 mL) was added bromine (3.0 mL, 58 mmol) dropwise slowly. The resulting mixture was heated to 60 °C and stirred for 1 h. The reaction mixture was cooled to room temperature, filtered, and washed with water. The precipitate was dried in a vacuum desiccator to give the title product as a white solid (6.03 g, 83% yield).

Mp: 165.9-166.4 °C. **^{1}H-NMR (300 MHz, DMSO-*d$_6$*)** δ: 7.45 (dd, *J* = 9.1, 1.8 Hz, 1H), 7.21 (t, *J* = 9.0 Hz, 1H), 3.86 (s, 3H), 3.35 (s, 1H). **^{13}C-NMR (126 MHz, DMSO-*d$_6$*)** δ: 164.5, 147.9 (d, *J* = 249.8 Hz), 146.8 (d, *J* = 10.0 Hz), 128.5 (d, *J* = 3.6 Hz), 126.1 (d, *J* = 18.2 Hz), 115.8, 107.5 (d, *J* = 3.6 Hz), 56.5. **^{19}F-NMR (282 MHz, DMSO-*d$_6$*)** δ: -134.77 (d, *J* = 8.6 Hz, 1F). **ATR-FTIR (KBr)**: *ν* = 3100, 3011, 2970, 2038, 1961, 1702, 1179, 1405, 720, 570 cm^{-1}. **MS (ESI)** *m/z*: [M-H]$^-$ 247.

Methyl 6-bromo-2-fluoro-3-methoxybenzoate

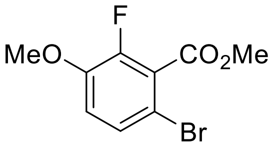

A 100 mL round bottom flask equipped with a deen-stark tube and condenser was charged with 6-bromo-2-fluoro-3-methoxy benzoic acid (5.73 g, 23.0 mmol), MeOH (23 mL), and conc. H_2SO_4 (1.0 mL). The resulting mixture was heated to reflux and stirred for 24 h. The reaction mixture was cooled to room temperature, concentrated, filtered, and washed with water. The resulting precipitate was dried in a vacuum desiccator to give the title product as a white solid (3.23 g, 55% yield).

Mp: 53.0-53.6 °C. **^{1}H-NMR (300 MHz, CDCl$_3$)** δ: 7.29 (dd, *J* = 8.9, 1.8 Hz, 1H), 6.89 (t, *J* = 8.7 Hz, 1H), 3.97 (s, 3H), 3.89 (s, 3H). **^{13}C-NMR (126 MHz, CDCl$_3$)** δ: 164.3, 149.8 (d, *J* = 255.2 Hz), 147.4 (d, *J* = 10.9 Hz), 128.3 (d, *J* = 4.5 Hz), 125.1 (d, *J* = 17.3 Hz), 115.6, 109.5, 56.8, 53.3. **^{19}F-NMR (282 MHz, CDCl$_3$)** δ: -133.18 (d, *J* = 8.6 Hz, 1F). **ATR-FTIR (KBr)**: *ν* = 3016, 2953, 2841, 1739, 1576, 1441, 1188, 1162, 805, 727 cm^{-1}. **MS (ESI)** *m/z*: [M+Na]$^+$ 285.

Methyl 6-cyano-2-fluoro-3-methoxybenzoate

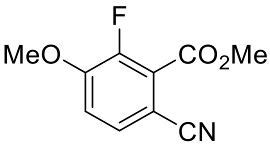

A 20 mL round bottom flask was charged with methyl 6-bromo-2-fluoro-3-methoxybenzoate (526.0 mg, 2.0 mmol), CuCN (358.0 mg, 4.0 mmol) and NMP (4.0 mL). The mixture was heated to 150 °C and stirred for 1 h. After cooling to room temperature, water and ammonium hydroxide (28%) was added to the reaction mixture and extracted with EtOAc. The combined organic layers were washed with brine, dried over Na_2SO_4, filtered, and concentrated under reduced pressure. The residue was purified by flash column chromatography (hexane/EtOAc = 7/3 to 1/1) to give the title product as a pale yellow solid (384.7 mg, 92% yield).

Mp: 127.1-127.9 °C. **^{1}H-NMR (300 MHz, $CDCl_3$)** δ: 7.52 (d, *J* = 8.5 Hz, 1H), 7.11 (t, *J* = 8.1 Hz, 1H), 4.00 (s, 3H), 3.97 (s, 3H). **^{13}C-NMR (126 MHz, $CDCl_3$)** δ: 162.6, 152.3 (d, *J* = 10.9 Hz), 150.8 (d, *J* = 260.7 Hz), 131.0 (d, *J* = 4.5 Hz), 123.5 (d, *J* = 13.6 Hz), 116.7 (d, *J* = 2.7 Hz), 115.3 (d, *J* = 2.7 Hz), 103.9, 56.8, 53.4. **^{19}F-NMR (282 MHz, $CDCl_3$)** δ: -130.26 (d, *J* = 6.9 Hz, 1F). **ATR-FTIR (KBr)**: *v* = 3101, 3040, 3019, 2993, 2227, 1725, 1445, 1208, 1154, 698 cm^{-1}. **MS (ESI)** *m/z*: $[M+Na]^+$ 232.

3-Fluoro-4-hydroxyphthalic acid

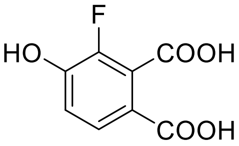

To a 0 °C solution of methyl 6-cyano-2-fluoro-3-methoxybenzoate (895.0 mg, 4.28 mmol) in CH_2Cl_2 was added BBr_3 (1.0 M in CH_2Cl_2, 21.0 mL, 21.4 mmol) dropwise slowly. The resulting mixture was stirred for 60 h at room temperature. The reaction mixture was poured into crushed ice and extracted with EtOAc/MeOH = 9/1 three times. The combined organic layers were washed with brine, dried over Na_2SO_4, filtered, and concentrated under reduced pressure. The resulting precipitate was filtered and washed with Et_2O to give the crude, which was used next reaction without further purification.

A 10 mL round bottom flask was charged with the crude (500 mg), AcOH (1.4 mL), water (1.4 mL), and conc. HCl (1.4 mL). The mixture was heated to reflux and stirred for 12 h. The reaction mixture was cooled to room temperature and filtered to give the first crystal (110 mg). The filtrate was concentrated, filtered, and washed with Et_2O to give the second crystal, the title product, as a pale yellow solid (425 mg, 77% yield).

Mp: 192.3-193.0 °C. **^{1}H-NMR (300 MHz, DMSO-*d*$_6$)** δ: 10.98 (s, 1H), 7.61 (d, *J* = 8.5 Hz, 1H), 7.03 (t, *J* = 8.5 Hz, 1H). **^{13}C-NMR (126 MHz, DMSO-*d*$_6$)** δ: 165.8, 165.8 (d, *J* = 1.8 Hz), 149.2 (d, *J* = 12.7 Hz), 147.0 (d, *J* = 242.5 Hz), 127.2, 126.6 (d, *J* = 17.3 Hz), 118.7, 117.2. **^{19}F-NMR (282 MHz, DMSO-*d*$_6$)** δ: -

140.56 (d, J = 6.9 Hz, 1F). **ATR-FTIR (KBr)**: ν = 3206, 2657, 2557, 2371, 2030, 1916, 1679, 1419, 990, 738 cm^{-1}. **MS (ESI)** *m/z*: [M-H]$^-$ 199.

2-(2,6-Dioxopiperidin-3-yl)-4-fluoro-5-hydroxyisoindoline-1,3-dione

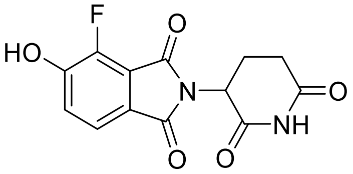

A test tube was charged with 3-aminopiperidine-2,6-dione hydrochloride (120 mg, 0.60 mmol) and Et_3N (0.21 mL, 1.5 mmol). The mixture was stirred for 3 h at room temperature, then 3-fluoro-4-hydroxyphthalic acid (100 mg, 0.50 mmol) and AcOH (1.5 mL) were added. The resulting mixture was heated to reflux and stirred for 4 h. The reaction mixture was cooled to room temperature, and crushed ice was added. The resulting precipitate was filtered and purified by flash column chromatography (EtOAc) to give the title product as a white solid (50.4 mg, 34% yield).

Mp: 286.1–293.1 °C (decomp.). **^{1}H-NMR (300 MHz, DMSO-*d*$_6$)** δ: 11.85-11.27 (brs, 1H), 11.10 (s, 1H), 7.58 (d, J = 7.9 Hz, 1H), 7.34 (t, J = 7.8 Hz, 1H), 5.09 (dd, J = 12.9, 5.3 Hz, 1H), 2.94-2.82 (m, 1H), 2.62-2.56 (m, 1H), 2.50-2.44 (m, 1H), 2.05-1.98 (m, 1H). **^{13}C-NMR (126 MHz, DMSO-*d*$_6$)** δ: 172.8, 169.9, 166.1, 164.1, 151.9 (d, J = 10.9 Hz), 145.9 (d, J = 261.6 Hz), 123.0, 121.6, 121.1, 118.7 (d, J = 9.1 Hz), 49.0, 31.0, 22.0. **^{19}F-NMR (282 MHz, DMSO-*d*$_6$)** δ: -137.35 (d, J = 6.9 Hz, 1F). **ATR-FTIR (KBr)**: ν = 3343, 3194, 3104, 2916, 1775, 1629, 1505, 998, 928, 840 cm^{-1}. **MS (ESI)** *m/z*: [M-H]$^-$ 291.

5-chloro-2-methyl-3-nitrobenzoic acid

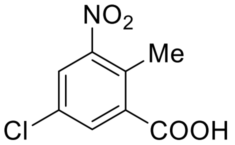

To a solution of 2-methyl-3-nitrobenzoic acid (5.05 g, 27.6 mmol) in conc. H_2SO_4 (39.4 mL) was added 1,3- dichloro-5,5-dimethyl hydantoin (5.43 g, 27.6 mmol) at 0 °C. The resulting mixture was heated to 100 °C and stirred for 12 h. The reaction mixture was cooled to room temperature, poured into crushed ice, and the resulting precipitate was filtered and washed with water. The precipitate was dried in a vacuum to give the title product as a brown solid (5.46 g, 91% yield).

Mp: 168.3-169.3 °C. **^{1}H-NMR (300 MHz, DMSO-*d*$_6$)** δ: 10.24 (s, 1H), 8.15 (d, J = 2.1 Hz, 1H), 7.98 (d, J = 2.3 Hz, 1H), 2.44 (s, 3H). **^{13}C-NMR (126 MHz, DMSO-*d*$_6$)** δ: 166.6, 152.0, 136.1, 132.6, 131.1, 129.8, 125.9, 15.2. **ATR-FTIR (KBr)**: ν = 3084, 3002, 2881, 2643, 1854, 1699, 1536, 1431, 783, 756 cm^{-1}. **MS (ESI)** *m/z*: [M-H]$^-$ 214.

5-Chloro-3-nitrophthalic acid

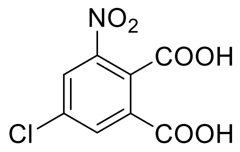

To a solution of 5-Chloro-2-methyl-3-nitrobenzoic acid (1.41 g, 6.5 mmol), NaOH (710 mg, 17.8 mmol) in water (13 mL) was added $KMnO_4$ (8.25 g, 52.2 mmol). The resulting mixture was heated to reflux and stirred for 5 h. The reaction mixture was cooled to room temperature, added $KMnO_4$ (8.25 g, 52.2 mmol), heated to reflux, and stirred for 6 h. The reaction mixture was cooled to room temperature, filtered through a pad of celite, and acidified to pH 1-2 with 12 mol/L HCl aq. The resulting precipitate was filtered and dried in a vacuum to give the title product as a white solid (1.3 g, 81% yield).

Mp: 176.8-177.7 °C. **^{1}H-NMR (300 MHz, DMSO-*d*$_6$)** δ: 13.04 (brs, 2H), 8.42 (d, *J* = 2.1 Hz, 1H), 8.21 (d, *J* = 2.1 Hz, 1H). **^{13}C-NMR (75 MHz, DMSO-*d*$_6$)** δ: 165.4, 164.9, 147.3, 134.6, 134.5, 133.2, 129.3, 127.6. **ATR-FTIR (KBr)**: *v* = 3262, 3083, 1714, 1538, 1467, 1354, 1273, 1161, 880, 692 cm^{-1}. **HRMS (ESI$^+$)**: *m/z* calcd for C8H3ClNO6 $[M-H]^-$: 243.9654 found: 243.9652.

6-Chloro-2-(2,6-dioxopiperidin-3-yl)-4-nitroisoindoline-1,3-dione

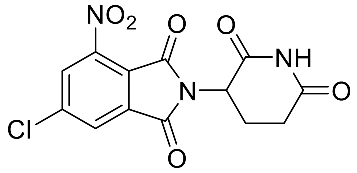

To a solution of 5-Chloro-3-nitrophthalic acid (98.2 mg, 0.40 mmol), 2-Aminoglutarimide hydrochloride (79.0 mg, 0.48 mmol) in AcOH (2.0 mL) was added AcONa (39.4 mg, 0.48 mmol). The resulting mixture was heated to reflux and stirred for 12 h. The reaction mixture was cooled to room temperature and added water. The resulting precipitate was filtered, washed with water, and dried in a vacuum desiccator to give the title product as a gray solid (74.2 mg, 52% yield).

Mp: 246.9-247.8 °C. **^{1}H-NMR (300 MHz, DMSO-*d*$_6$)** δ: 11.20 (s, 1H), 8.56 (d, *J* = 1.8 Hz, 1H), 8.41 (d, *J* = 1.5 Hz, 1H), 5.21 (dd, *J* = 12.8, 5.4 Hz, 1H), 2.95-2.83 (m, 1H), 2.64-2.54 (m, 1H), 2.46-2.41 (m, 1H), 2.09-2.04 (m, 1H). **^{13}C-NMR (126 MHz, DMSO-*d*$_6$)** δ: 172.7, 169.3, 164.2, 161.9, 144.7, 140.8, 134.6, 128.7, 127.5, 121.2, 49.6, 30.9, 21.7. **ATR-FTIR (KBr)**: *v* = 3483, 3381, 1702, 1636, 1408, 1369, 1262, 1196, 752, 607 cm^{-1}. **HRMS (ESI$^+$)**: *m/z* calcd for C13H7ClN3O6 [M-H]-: 336.0029 found: 336.0019.

4-amino-6-chloro-2-(2,6-dioxopiperidin-3-yl)isoindoline-1,3-dione (NE-011)

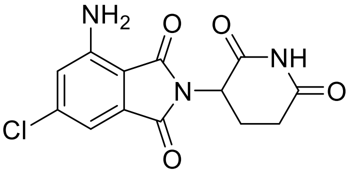

A 30 mL flask was charged with 6-chloro-2-(2,6-dioxopiperidin-3-yl)-4-nitroisoindoline-1,3-dione (100 mg, 0.30 mmol), Fe (50 mg, 0.89 mmol), NH_4Cl (95 mg, 1.8 mmol) in water (2 mL), Acetone (5 mL). The resulting mixture was heated to reflux and stirred for 10 h. The reaction mixture was filtered through a pad of celite and concentrated under reduced pressure. The resulting aqueous layer was extracted with EtOAc. The combined organic layers were washed with brine, dried over Na_2SO_4, and concentrated under reduced pressure to give the title product a yellow solid (66.5 mg, 73% yield).

Mp: 271.7-272.7 °C. **^{1}H-NMR (300 MHz, DMSO-*d*$_6$)** δ: 11.12 (s, 1H), 7.05 (s, 1H), 7.01 (s, 1H), 6.74 (s, 2H), 5.06 (dd, J = 12.9, 5.3 Hz, 1H), 2.92-2.81 (m, 1H), 2.72-2.55 (m, 2H), 2.08-1.99 (m, 1H). **^{13}C-NMR (176 MHz, DMSO-*d*$_6$)** δ: 172.8, 170.0, 167.8, 166.3, 147.4, 140.0, 134.0, 120.0, 110.8, 107.8, 48.7, 31.0, 22.1. **ATR-FTIR (KBr)**: *v* = 3457, 3356, 3219, 3097, 1703, 1406, 1261, 1126, 970, 610 cm^{-1}. **HRMS (ESI$^+$)**: *m/z* calcd for C13H9ClN3O4 $[M-H]^-$: 306.0287 found: 306.0278.

methyl 5-chloro-2-methyl-3-nitrobenzoate

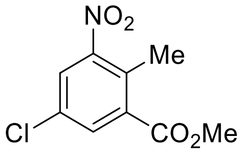

To a solution of 5-chloro-2-methyl-3-nitrobenzoic acid (1 g, 4.6 mmol) in MeOH (46 mL) was added $SOCl_2$ (1.0 mL, 13.9 mmol) dropwise slowly. The resulting mixture was heated to reflux and stirred for 14 h. The reaction mixture was cooled to room temperature and added water. The resulting precipitate was filtered and dried in a vacuum desiccator to give the title as a white solid (836 mg, 79% yield).

Mp: 45.2-46.1 °C. **^{1}H-NMR (300 MHz, $CDCl_3$)** δ: 7.98 (d, *J* = 2.3 Hz, 1H), 7.84 (d, *J* = 2.1 Hz, 1H), 3.96 (s, 3H), 2.58 (s, 3H). **^{13}C-NMR (75 MHz, $CDCl_3$)** δ: 165.7, 152.2, 134.5, 133.7, 131.9 (d, *J* = 34.8 Hz), 126.8, 53.0, 15.9. **ATR-FTIR (KBr)**: *v* = 3076, 1734, 1535, 1437, 1365, 1298, 1265, 1038, 795, 720 cm^{-1}. **MS (ESI)** *m/z*: $[M+Na]^+$ 252.

methyl 2-(bromomethyl)-5-chloro-3-nitrobenzoate

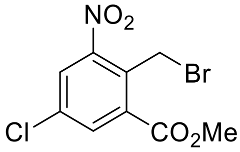

To a solution of methyl 5-Chloro-2-methyl-3-nitrobenzoate (602 mg, 2.6 mmol), *N*-Bromosuccinimide

(560 mg, 3.2 mmol) in degassed CCl_4 (3.7 mL) was added 75% Benzoyl peroxide (84.7 mg, 0.26 mmol). The resulting mixture was heated to reflux and stirred for 14 h. The reaction mixture was cooled to room temperature, filtered, and concentrated under reduced pressure. The resulting precipitate was purified by flash column chromatography (Hexane/EtOAc = 95/5 to 9/1) to give the title product as a yellow solid (433 mg, 54% yield).

Mp: 39.9-40.5 °C. **^{1}H-NMR (300 MHz, $CDCl_3$)** δ: 8.10 (d, *J* = 2.3 Hz, 1H), 7.96 (d, *J* = 2.3 Hz, 1H), 5.11 (s, 2H), 4.01 (s, 3H). **^{13}C-NMR (75 MHz, $CDCl_3$)** δ: 164.9, 151.0, 135.2, 135.0, 133.6, 131.4, 128.0, 53.5, 22.2. **ATR-FTIR (KBr)**: *v* = 3087, 2950, 1733, 1537, 1433, 1347, 1201, 1156, 773, 605 cm^{-1}. **MS (ESI)** *m/z*: $[M-H]^-$ 306.

3-(6-chloro-4-nitro-1-oxoisoindolin-2-yl)piperidine-2,6-dione

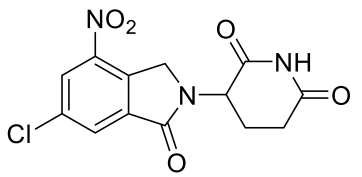

To a solution of methyl 2-(bromomethyl)-5-chloro-3-nitrobenzoate (224 mg, 0.73 mmol), 2-Aminoglutarimide hydrochloride (143 mg, 0/87 mmol) in DMF (0.7 mL) was added Et_3N (0.1mL, 0.87 mmol). The resulting mixture was heated to 60 °C and stirred for 4 h. The reaction mixture was cooled to room temperature and added water. The resulting precipitate was filtered and dried in a vacuum desiccator to give the title product as a gray solid (146 mg, 62% yield).

Mp: 253.3-254.1 °C. **^{1}H-NMR (300 MHz, DMSO-*d*$_6$)** δ: 11.08 (s, 1H), 8.52 (d, *J* = 1.8 Hz, 1H), 8.28 (d, *J* = 1.6 Hz, 1H), 5.20 (dd, *J* = 13.3, 5.1 Hz, 1H), 4.85 (dd, *J* = 34.6, 19.3 Hz, 2H), 2.99-2.87 (m, 1H), 2.74-2.56 (m, 2H), 2.05-1.99 (m, 1H). **^{13}C-NMR (176 MHz, DMSO-*d*$_6$)** δ: 172.8, 170.6, 164.8, 143.9, 136.2, 136.1, 134.3, 129.3 (d, *J* = 26.8 Hz), 126.8 (d, *J* = 31.8 Hz), 52.0, 48.4, 31.2, 22.2. **ATR-FTIR (KBr)**: *v* = 3087, 1691, 1537, 1450, 1353, 1322, 1192, 889, 731, 531 cm^{-1}. **HRMS (ESI$^+$)**: *m/z* calcd for C13H9ClN3O5 $[M-H]^-$: 322.0236 found: 322.0234.

3-(4-amino-6-chloro-1-oxoisoindolin-2-yl)piperidine-2,6-dione

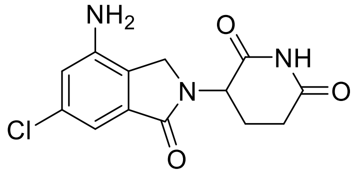

A 100 mL round bottom flask was charged with 3-(6-chloro-4-nitro-1-oxoisoindolin-2-yl)piperidine-2,6-dione (850 mg, 2.63 mmol), Fe (1.12 g, 20.0 mmol), NH_4Cl (1.12 mg, 21.0 mmol) in water (6.6 mL), EtOH (33 mL). The resulting mixture was heated to reflux and stirred for 3 h. The reaction mixture was filtered through a pad of celite and concentrated under reduced pressure. The resulting mixture was extracted with

EtOAc, and combined organic layers were washed with brine, dried over Na_2SO_4, and concentrated under reduced pressure to give the title product, a pale yellow solid (314 mg, 41% yield).

Mp: 242.7-243.7 °C. **^{1}H-NMR (300 MHz, DMSO-*d*$_6$)** δ: 11.04 (s, 1H), 6.85 (s, 1H), 6.80 (s, 1H), 5.82 (s, 2H), 5.10 (dd, *J* = 13.1, 4.9 Hz, 1H), 4.14 (dd, *J* = 35.7, 17.2 Hz, 2H), 2.95-2.85 (m, 1H), 2.73-2.63 (m, 1H), 2.31-2.27 (m, 1H), 2.06-2.01 (m, 1H). **^{13}C-NMR (126 MHz, DMSO-*d*$_6$)** δ: 172.9, 171.1, 167.6, 145.1, 133.9, 133.4, 124.6, 115.2, 109.5, 51.7, 45.5, 31.2, 22.7. **ATR-FTIR (KBr)**: *ν* = 3455, 3347, 3036, 2847, 1685, 1671, 1487, 1356, 1029, 957 cm^{-1}. **HRMS (ESI$^+$)**: *m/z* calcd for C13H11ClN3O3 $[M-H]^-$: 292.0494 found: 292.0487.

5-Bromo-3-nitrophthalic acid

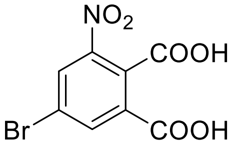

To a solution of 5-bromo-2-methyl-3-nitrobenzoic acid (7.09 g, 26.9 mmol), NaOH (4.31 g, 108 mmol) in water (104 mL) was added $KMnO_4$ (34.0 g, 215 mmol). The resulting mixture was heated to reflux and stirred for 1 h. The reaction mixture was cooled to room temperature, added $KMnO_4$ (8.50 g, 215 mmol), heated to reflux, and stirred for 1 h. The reaction mixture was cooled to room temperature, filtered through a pad of celite, and acidified to pH 1-2 with 12 mol/L HCl aq. The resulting precipitate was filtered and dried in a vacuum to give the title product as a white solid (2.33 g, 29% yield).

Mp: 169.8-170.7 °C. **^{1}H-NMR (300 MHz, DMSO-*d*$_6$)** δ: 8.53 (s, 1H), 8.33 (s, 1H). **^{13}C-NMR (75 MHz, DMSO-*d*$_6$)** δ: 165.4, 164.7, 147.4, 137.2, 135.5, 133.2, 130.1, 122.5. **ATR-FTIR (KBr)**: *ν* = 3406, 3075, 1735, 1546, 1348, 1259, 670, 596, 522, 510 cm^{-1}. **HRMS (ESI$^+$)**: *m/z* calcd for C8H3BrNO6 $[M-H]^-$: 287.9149 found: 287.9145.

6-Bromo-2-(2,6-dioxopiperidin-3-yl)-4-nitroisoindoline-1,3-dione

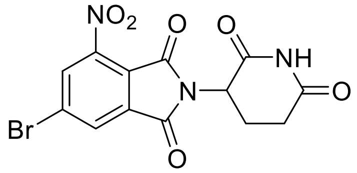

To a solution of 5-bromo-3-nitrophthalic acid (1.07 g, 3.68 mmol), 2-Aminoglutarimide hydrochloride (908 mg, 5.5 mmol) in AcOH (14.7 mL) was added AcONa (302 mg, 3.7 mmol). The resulting mixture was heated to reflux and stirred for 2 h. The reaction mixture was cooled to room temperature and added water. The resulting precipitate was filtered, washed with water, and dried in a vacuum desiccator to give the title product as a white solid (1.00 g, 71% yield).

Mp: 282.6-283.2 °C. **^{1}H-NMR (300 MHz, DMSO-*d*$_6$)** δ: 11.19 (s, 1H), 8.66 (d, *J* = 0.9 Hz, 1H), 8.51 (d,

J = 1.2 Hz, 1H), 5.20 (dd, J = 12.8, 5.4 Hz, 1H), 2.89-2.82 (m, 1H), 2.73-2.57 (m, 2H), 2.10-2.02 (m, 1H). **^{13}C-NMR (176 MHz, DMSO-*d*$_6$)** δ: 172.7, 169.3, 164.1, 162.1, 144.6, 134.4, 131.4, 130.2, 129.2, 121.5, 49.5, 30.8, 21.7. **ATR-FTIR (KBr)**: *v* = 3734, 3649, 2363, 1724, 1542, 1421, 1377, 1273, 1193, 646 cm^{-1}. **HRMS (ESI$^+$)**: *m/z* calcd for C13H7BrN3O6 [M-H]$^-$: 379.9524 found: 379.9523.

4-amino-6-bromo-2-(2,6-dioxopiperidin-3-yl)isoindoline-1,3-dione (NE-012)

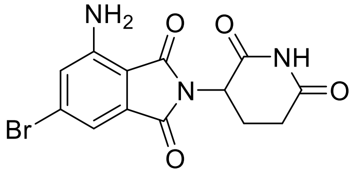

A 30 mL flask was charged with 6-bromo-2-(2,6-dioxopiperidin-3-yl)-4-nitroisoindoline-1,3-dione (304 mg, 0.8 mmol), Fe (341 mg, 6.1 mmol) in EtOH (10 mL), water (2.0 mL). The resulting mixture was heated to 50 °C and stirred for 2 h at room temperature. The reaction mixture was filtered through a pad of celite, and washed with EtOH, and THF. The filtrate was concentrated, diluted with water, and extracted with EtOAc. The combined organic layers were washed with brine, dried over Na_2SO_4, and concentrated under reduced pressure to give the title product as a white solid (77 mg, 27% yield).

Mp: 276.6-277.5 °C. **^{1}H-NMR (300 MHz, DMSO-*d*$_6$)** δ: 11.11 (s, 1H), 7.23 (d, J = 0.9 Hz, 1H), 7.11 (d, J = 0.9 Hz, 1H), 6.72 (s, 1H), 5.05 (dd, J = 13.2, 5.3 Hz, 1H), 2.94-2.82 (m, 1H), 2.73-2.61 (m, 2H), 2.06-1.99 (m, 1H). **^{13}C-NMR (176 MHz, DMSO-*d*$_6$)** δ: 172.8, 170.0, 167.9, 166.3, 147.5, 133.8, 128.7, 123.1, 113.4, 108.0, 48.7, 31.0, 22.1. **ATR-FTIR (KBr)**: *v* = 3480, 3377, 2364, 1702, 1635, 1593, 1405, 1262, 1192, 1103 cm^{-1}. **HRMS (ESI$^+$)**: *m/z* calcd for C13H9BrN3O4 [M-H]-: 349.9782 found: 349.9775.

methyl 5-bromo-2-(bromomethyl)-3-nitrobenzoate

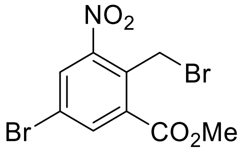

To a solution of methyl 5-bromo-2-methyl-3-nitrobenzoate (4.1 g, 15 mmol), *N*-Bromosuccinimide (3.2 g, 18 mmol) in degassed CCl_4 (21 mL) was added Benzoyl peroxide (363 mg, 1.5 mmol). The resulting mixture was heated to reflux and stirred for 3 h. The reaction mixture was cooled to room temperature, filtered, and concentrated under reduced pressure. The residue was purified by flash column chromatography (Hexane/EtOAc = 95/5 to 9/1) to give the title product as a white solid (4.02 g, 76% yield).

Mp: 60.0-60.9 °C. ^{1}**H-NMR (300 MHz, CDCl$_3$)** δ: 8.24 (d, J = 1.8 Hz, 1H), 8.09 (d, J = 2.1 Hz, 1H), 5.10 (s, 2H), 4.01 (s, 3H). **^{13}C-NMR (176 MHz, CDCl$_3$)** δ: 164.8, 150.9, 137.8 (d, J = 23.4 Hz), 133.7, 131.8, 130.9, 122.6, 53.5, 22.3. **ATR-FTIR (KBr)**: *v* = 3087, 2948, 1733, 1534, 1441, 1345, 1266, 1202, 1145, 792 cm^{-1}. **MS (ESI)** *m/z*: [M-H]$^-$ 350.

3-(6-bromo-4-nitro-1-oxoisoindolin-2-yl)piperidine-2,6-dione

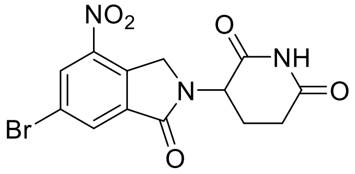

To a solution of methyl 5-bromo-2-(bromomethyl)-3-nitrobenzoate (564 mg, 2.8 mmol), 2-Aminoglutarimide hydrochloride (561 mg, 3.4 mmol) in DMF (3.9 mL) was added K_2CO_3 (979 mg, 7.1 mmol). The resulting mixture was heated to 50 °C and stirred for 2 h. The reaction mixture was cooled to room temperature and added water. The resulting precipitate was filtered and dried in a vacuum desiccator to give the title product as a gray solid (564 mg, 54% yield).

Mp: 226.6-227.6 °C. **^{1}H-NMR (300 MHz, DMSO-*d*$_6$)** δ: 11.06 (s, 1H), 8.59 (d, *J* = 1.5 Hz, 1H), 8.37 (d, *J* = 1.5 Hz, 1H), 5.21-5.15 (m, 1H), 4.81 (dd, *J* = 34.3, 19.3 Hz, 2H), 2.97-2.87 (m, 1H), 2.72-2.62 (m, 2H), 2.03-1.99 (m, 1H). **^{13}C-NMR (176 MHz, DMSO-*d*$_6$)** δ: 172.8, 170.6, 164.7, 144.0, 136.4 (d, *J* = 20.1 Hz), 132.0, 129.4, 121.8, 52.0, 48.4, 31.1, 22.2. **ATR-FTIR (KBr)**: *v* = 3208, 3108, 3074, 1711, 1532, 1442, 1354, 1209, 1182, 720 cm^{-1}. **HRMS (ESI$^+$)**: *m/z* calcd for C13H9BrN3O5 [M-H]$^-$: 365.9731 found: 365.9723.

3-(4-amino-6-bromo-1-oxoisoindolin-2-yl)piperidine-2,6-dione

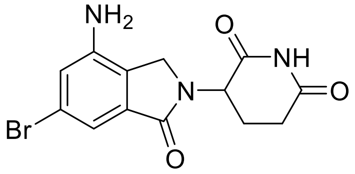

A 30 mL flask was charged with 3-(7-fluoro-4-nitro-1-oxoisoindolin-2-yl)piperidine-2,6-dione (198 mg, 0.54 mmol), Fe (231 mg, 4.1 mmol) in EtOH (0.8 mL), water (1.4 mL). The resulting mixture was heated to 60 °C and stirred for 6 h. The reaction mixture was filtered through a pad of celite, and washed with EtOH, and THF. The filtrate was concentrated, diluted with water, and extracted with EtOAc. The combined organic layers were washed with brine, dried over Na_2SO_4, and concentrated under reduced pressure to give the title product as a white solid (36.6 mg, 20% yield).

Mp: 237.9-238.7 °C. **^{1}H-NMR (300 MHz, DMSO-*d*$_6$)** δ: 11.03 (s, 1H), 6.97-6.95 (m, 2H), 5.80 (s, 2H), 5.12- 5.06 (m, 1H), 4.12 (dd, *J* = 35.5, 17.6 Hz, 2H), 2.95-2.87 (m, 1H), 2.73-2.63 (m, 2H), 2.05-1.99 (m, 1H). **^{13}C-NMR (176 MHz, DMSO-*d*$_6$)** δ: 172.9, 171.1, 167.5, 145.4, 134.2, 124.9, 121.7, 118.0, 112.4, 51.7, 45.6, 39.5, 31.2, 22.7. **ATR-FTIR (KBr)**: *v* = 3425, 2186, 3083, 1681, 1605, 1481, 1462, 1335, 1199, 1026 cm^{-1}. **HRMS (ESI$^+$)**: *m/z* calcd for C13H11BrN3O3 [M-H]$^-$: 335.9989 found: 335.9974.

2-methyl-3-nitro-5-(trifluoromethyl)benzoic acid

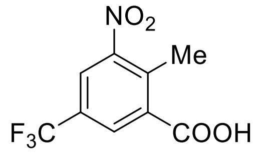

To a solution of 2-methyl-5-(trifluoromethyl)benzoic acid (1.0 g, 4.9 mmol) in conc. H_2SO_4 (10 mL) was added fuming HNO_3 (1 mL) dropwise slowly at 0 °C. The resulting mixture was stirred for 2 h at room temperature. The reaction mixture was poured into crushed ice, and the resulting precipitate was filtered and washed with water. The precipitate was dried in a vacuum to give the title product as a white solid (1.2 g, 96% yield).

Mp: 134.9-135.5 °C. **^{1}H-NMR (300 MHz, DMSO-*d$_6$*)** δ: 8.43 (s, 1H), 8.24 (s, 1H), 2.57 (s, 3H). **^{13}C-NMR (126 MHz, DMSO-*d$_6$*)** δ: 166.5, 151.8, 135.8, 135.8, 129.2 (d, J = 3.6 Hz), 127.6 (q, J = 33.9 Hz), 123.2 (d, J = 3.6 Hz), 122.7 (q, J = 272.8 Hz), 15.9. **^{19}F-NMR (282 MHz, DMSO-*d$_6$*)** δ: -61.08 (s, 3F). **ATR-FTIR (KBr)**: v = 3092, 2992, 2879, 2642, 2499, 1703, 1539, 1442, 1028, 747 cm^{-1}. **MS (ESI)** *m/z*: [M-H]$^-$ 248.

methyl 2-methyl-3-nitro-5-(trifluoromethyl)benzoate

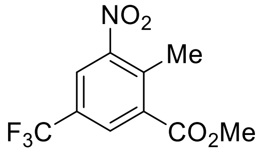

To a solution of 2-methyl-3-nitro-5-(trifluoromethyl)benzoic acid (1.2 g, 4.9 mmol) in MeOH (25 mL) was added $SOCl_2$ (1.1 mL, 15 mmol) dropwise slowly. The resulting mixture was heated to reflux and stirred for 12 h. The reaction mixture was cooled to room temperature and added $NaHCO_3$ aq. Then the solvent was removed under reduced pressure and extracted with CH_2Cl_2. The combined organic layers were washed with brine, dried over Na_2SO_4, and concentrated. The resulting mixture was purified by flash column chromatography (Hexane/EtOAc = 4/1) to give the title product as a colorless oil (1.08 g, 84% yield).

^{1}H-NMR (300 MHz, $CDCl_3$) δ: 8.27 (s, 1H), 8.11 (s, 1H), 3.98 (s, 3H), 2.70 (s, 3H). **^{13}C-NMR (126 MHz, $CDCl_3$)** δ: 165.6, 152.1, 137.4, 134.4, 130.4 (d, J = 3.6 Hz), 129.5 (q, J = 34.8 Hz), 123.8 (d, J = 3.6 Hz), 122.60 (q, J = 272.8 Hz), 53.2, 16.6. **^{19}F-NMR (282 MHz, $CDCl_3$)** δ: -63.46 (s, 3F). **ATR-FTIR (KBr)**: v = 3092, 3008, 2959, 2905, 1735, 1542, 1439, 1294, 970, 873 cm^{-1}. **MS (ESI)** *m/z*: [M+Na]$^+$ 286.

methyl 2-(bromomethyl)-3-nitro-5-(trifluoromethyl)benzoate

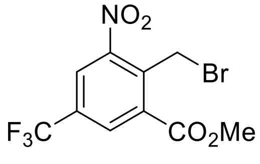

To a solution of methyl 2-methyl-3-nitro-5-(trifluoromethyl)benzoate (646 mg, 3.2 mmol), *N*-Bromosuccinimide (1.3 g, 6.3 mmol) in degassed CCl_4 (4.5 mL) was added 75% Benzoyl peroxide (102 mg, 0.32 mmol). The resulting mixture was heated to reflux and stirred for 18 h. The reaction mixture was cooled to room temperature, filtered, and concentrated under reduced pressure. The resulting mixture was purified by flash column chromatography (Hexane/EtOAc = 95/5 to 9/1) to give the title product as a yellow solid (734 mg, 68% yield).

Mp: 40.3-41.2 °C. **^{1}H-NMR (300 MHz, $CDCl_3$)** δ: 8.36 (s, 1H), 8.21 (s, 1H), 5.18 (s, 2H), 4.04 (s, 3H). **^{13}C-NMR (126 MHz, $CDCl_3$)** δ: 164.7, 150.8, 136.8, 133.6, 131.8 (q, J = 35.1 Hz), 131.6 (d, J = 2.7 Hz), 125.0 (d, J = 3.6 Hz), 122.2 (q, J = 273.4 Hz), 53.7, 21.7. **^{19}F-NMR (282 MHz, $CDCl_3$)** δ: -63.74 (s, 3F). **ATR-FTIR (KBr)**: ν = 3096, 3016, 2961, 2349, 1733, 1542, 1425, 1181, 982, 768, 586 cm^{-1}. **MS (ESI)** *m/z*: [M+H]+ 342.

3-(4-nitro-1-oxo-6-(trifluoromethyl)isoindolin-2-yl)piperidine-2,6-dione

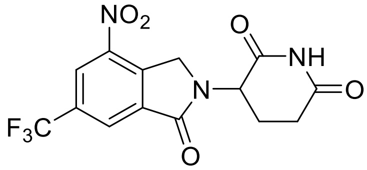

To a solution of methyl 2-(bromomethyl)-3-nitro-5-(trifluoromethyl)benzoate (616 mg, 1.8 mmol), 2-Aminoglutarimide hydrochloride (346 mg, 2.1 mmol) in DMF (1.8 mL) was added Et_3N (0.5 mL, 3.5 mmol). The resulting mixture was heated to 50 °C and stirred for 14 h. The reaction mixture was cooled to room temperature and added water. The resulting precipitate was filtered, washed with MeOH, and THF, and dried in a vacuum desiccator to give the title product as a purple solid (197 mg, 31% yield).

Mp: 264.3-268.3 °C (decomp.). **^{1}H-NMR (300 MHz, DMSO-*d$_6$*)** δ: 11.08 (s, 1H), 8.71 (s, 1H), 8.52 (s, 1H), 5.23 (dd, J = 12.9, 5.0 Hz, 1H), 4.96 (dd, J = 33.7, 19.9 Hz, 2H), 2.98-2.86 (m, 1H), 2.63-2.54 (m, 2H), 2.03 (m, J = 5.1 Hz, 1H).

^{13}C-NMR (126 MHz, DMSO-*d$_6$*) δ: 172.8, 170.5, 164.8, 144.0, 141.4, 136.1, 130.7 (q, J = 33.9 Hz), 125.9 (d, J = 3.6 Hz), 124.0 (d, J = 17.3 Hz), 122.8 (q, J = 273.1 Hz), 52.0, 48.8, 31.2, 22.1. **^{19}F-NMR (282 MHz, DMSO-*d$_6$*)** δ: -60.46 (s, 3F). **ATR-FTIR (KBr)**: v = 3382, 3199, 3101, 2960, 2905, 1725, 1541, 1409, 980, 787 cm^{-1}. **MS (ESI)** *m/z*: $[M-H]^-$ 356.

3-(4-amino-1-oxo-6-(trifluoromethyl)isoindolin-2-yl)piperidine-2,6-dione

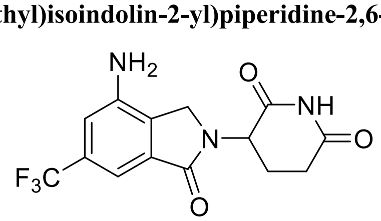

A 10 mL flask was charged with 3-(4-nitro-1-oxo-6-(trifluoromethyl)isoindolin-2-yl)piperidine-2,6-dione

(104 mg, 0.29 mmol), Pd/C (20 mg, 20 wt %) in THF (10 mL), MeOH (10 mL). The mixture was stirred at room temperature under H_2 (balloon) for 1 h. The reaction mixture was filtered through a pad of celite, and the filtrate was concentrated, and filtered to give the title product as a white solid (77 mg, 81% yield).

Mp: 287.5-292.9 °C (decomp.). **^{1}H-NMR (300 MHz, DMSO-*d$_6$*)** δ: 11.22-10.84 (1H), 7.11 (s, 1H), 7.09 (s, 1H), 6.00 (s, 2H), 5.14 (dd, *J* = 13.2, 5.3 Hz, 1H), 4.24 (dd, *J* = 36.3, 17.7 Hz, 2H), 2.96-2.86 (m, 1H), 2.73-2.60 (m, 1H), 2.39-2.25 (m, 1H), 2.08-2.01 (m, 1H). **^{13}C-NMR (126 MHz, DMSO-*d$_6$*)** δ: 172.9, 171.1, 167.7, 144.6, 133.2, 130.1 (q, *J* = 31.2 Hz), 129.2, 124.3 (q, *J* = 272.5 Hz), 111.9 (d, *J* = 2.7 Hz), 106.1 (d, *J* = 3.6 Hz), 51.7, 45.9, 31.2, 22.7. **^{19}F-NMR (282 MHz, DMSO-*d$_6$*)** δ: -60.64 (s, 3F). **ATR-FTIR (KBr)**: *v* = 3469, 3374, 3183, 3094, 2896, 2832, 1631, 1424, 1031, 893 cm^{-1}. **MS (ESI)** *m/z*: [M+Na]$^+$ 350.

methyl 2-methyl-3-nitro-5-(trifluoromethoxy)benzoate

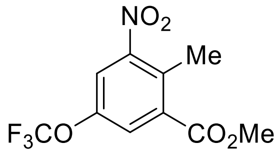

To a solution of 2-methyl-5-(trifluoromethoxy)benzoic acid (500 mg, 2.3 mmol) in conc. H_2SO_4 (4.5 mL) was added fuming HNO_3 (0.45 mL) dropwise slowly at 0 °C. The resulting mixture was stirred at room temperature for 4 h. The reaction mixture was poured into crushed ice and the resulting precipitate was filtered and washed with water. The precipitate was dried in a vacuum to give the crude (517 mg), which was used next reaction without further purification.

To a solution of the crude (517 mg) in MeOH (3.9 mL) was added $SOCl_2$ (0.42 mL, 5.9 mmol) dropwise slowly. The resulting mixture was heated to reflux and stirred for 12 h. The reaction mixture was cooled to room temperature and concentrated under reduced pressure. The resulting mixture was purified by flash column chromatography (Hexane/EtOAc = 20/1 to 15/1) to give the title product as a pale yellow oil (267 mg, 42% yield, 2 steps).

^{1}H-NMR (300 MHz, CDCl$_3$) δ: 7.89 (s, 1H), 7.75 (s, 1H), 3.97 (s, 3H), 2.64 (s, 3H). **^{13}C-NMR (126 MHz, CDCl$_3$)** δ: 165.5, 152.3, 146.5 (d, *J* = 1.8 Hz), 134.9, 132.1, 126.3, 120.3 (q, *J* = 260.0 Hz), 119.6, 53.1, 16.0. **^{19}F-NMR (282 MHz, CDCl$_3$)** δ: -58.7 (s, 3F). **ATR-FTIR (KBr)**: *v* = 3092, 2959, 1736, 1541, 1467, 1439, 1360, 1036, 1008, 887 cm^{-1}.

MS (ESI) *m/z*: [M-H]$^-$ 278.

methyl 2-(bromomethyl)-3-nitro-5-(trifluoromethoxy)benzoate

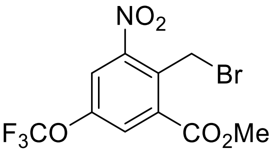

To a solution of methyl 2-methyl-3-nitro-5-(trifluoromethoxy)benzoate (722 mg, 2.5 mmol), *N*-Bromosuccinimide (893 g, 5.0 mmol) in degassed CCl_4 (3.6 mL) was added 75% Benzoyl peroxide (81 mg, 0.25 mmol). The resulting mixture was heated to reflux and stirred for 14 h. The reaction mixture was cooled to room temperature, filtered, and concentrated under reduced pressure. The resulting mixture was purified by flash column chromatography (Hexane/EtOAc = 95/5 to 9/1) to give the title product as a pale yellow oil (734 mg, 68% yield).

^{1}H-NMR (300 MHz, $CDCl_3$) δ: 7.97 (s, 1H), 7.84 (s, 1H), 5.14 (s, 2H), 4.03 (s, 3H). **^{13}C-NMR (126 MHz, $CDCl_3$)** δ: 164.7, 151.2, 148.4, 134.2, 131.5, 127.0, 120.4, 120.2 (q, *J* = 261.0 Hz), 53.7, 22.0. **^{19}F-NMR (282 MHz, $CDCl_3$)** δ: -58.5 (s, 3F). **ATR-FTIR (KBr)**: *ν* = 3092, 3012, 2958, 1734, 1544, 1437, 1010, 976, 874, 615 cm^{-1}. **MS (ESI)** *m/z*: $[M+Na]^+$ 380.

3-(4-nitro-1-oxo-6-(trifluoromethoxy)isoindolin-2-yl)piperidine-2,6-dione

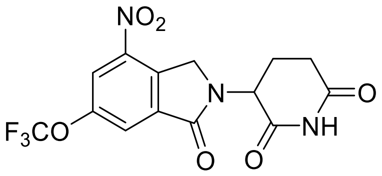

To a solution of methyl 2-(bromomethyl)-3-nitro-5-(trifluoromethoxy)benzoate (104 mg, 0.28 mmol), 2-Aminoglutarimide hydrochloride (55 mg, 0.34 mmol) in DMF (0.28 mL) was added Et_3N (0.08 mL, 0.6 mmol). The resulting mixture was heated to 50 °C and stirred for 14 h. The reaction mixture was cooled to room temperature and added water. The resulting precipitate was filtered, washed with MeOH, and THF, and dried in a vacuum desiccator to give the title product as a purple solid (78 mg, 46% yield).

Mp: 244.5-249.2 °C (decomp.). **^{1}H-NMR (500 MHz, DMSO-*d_6*)** δ: 11.06 (s, 1H), 8.46 (d, *J* = 1.5 Hz, 1H), 8.20 (d, *J* = 0.9 Hz, 1H), 5.19 (dd, *J* = 13.1, 5.2 Hz, 1H), 4.88 (dd, *J* = 47.8, 19.4 Hz, 2H), 2.95-2.87 (m, 1H), 2.73-2.53 (m, 2H), 2.05-1.99 (m, 1H). **^{13}C-NMR (126 MHz, DMSO-*d_6*)** δ: 172.8, 170.5, 164.8, 148.3, 144.1, 136.4, 122.3, 120.5, 119.9 (q, *J* = 258.5 Hz), 52.1, 48.5, 31.1, 22.1. **^{19}F-NMR (282 MHz, DMSO-*d_6*)** δ: -57.0 (s, 3F). **ATR-FTIR (KBr)**: *ν* = 3437, 3201, 3106, 2900, 1725, 1544, 1409, 982, 907, 879 cm^{-1}. **MS (ESI)** *m/z*: $[M-H]^-$ 372.

3-(4-amino-1-oxo-6-(trifluoromethoxy)isoindolin-2-yl)piperidine-2,6-dione

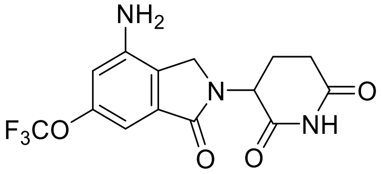

A 10 mL flask was charged with 3-(4-nitro-1-oxo-6-(trifluoromethoxy)isoindolin-2-yl)piperidine-2,6-dione (50 mg, 0.13 mmol), Pd/C (10 mg, 20 wt %) in THF (5 mL), MeOH (5 mL). The resulting mixture was stirred at room temperature under H_2 (balloon) for 1 h. The reaction mixture was filtered through a pad of celite, and the filtrate was concentrated, and filtered to give the title product as a white solid (16 mg, 34% yield).

Mp: 241.7-246.9 °C (decomp.). **^{1}H-NMR (500 MHz, DMSO-*d*$_6$)** δ: 11.02 (s, 1H), 6.75 (d, *J* = 0.9 Hz, 1H), 6.72 (d, *J* = 0.9 Hz, 1H), 5.92 (s, 2H), 5.11 (dd, *J* = 13.1, 5.2 Hz, 1H), 4.18 (dd, *J* = 53.7, 17.1 Hz, 2H), 2.95-2.88 (m, 1H), 2.63-2.59 (m, 1H), 2.36-2.25 (m, 1H), 2.06-2.02 (m, 1H). **^{13}C-NMR (126 MHz, DMSO-*d*$_6$)** δ: 172.9, 171.1, 167.7, 149.5, 145.4, 133.7, 124.6, 120.1 (q, *J* = 255.8 Hz), 108.0, 101.7, 51.8, 45.6, 31.2, 22.7. **^{19}F-NMR (282 MHz, DMSO-*d*$_6$)** δ: -56.4 (s, 3F). **ATR-FTIR (KBr)**: *v* = 3442, 3368, 3239, 3197, 3101, 2896, 1644, 1424, 849, 738 cm^{-1}. **MS (ESI)** *m/z*: [M-H]$^-$ 342.

Synthesis of PROTAC

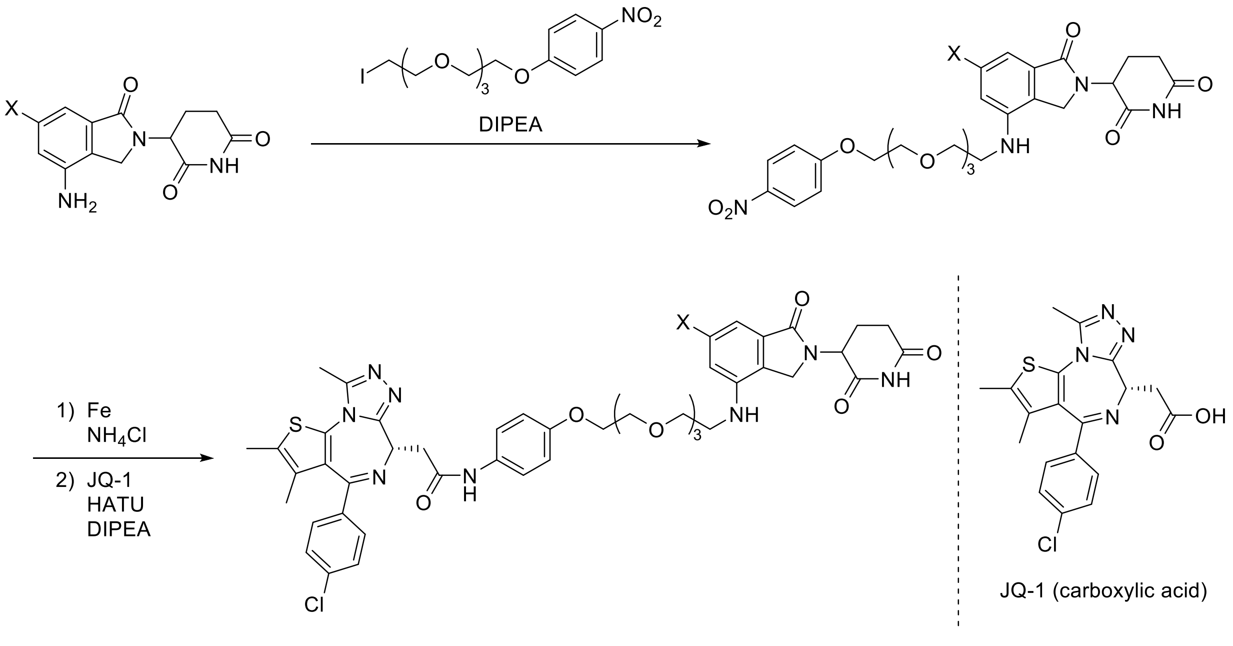

3-(4-((2-(2-(2-(2-(4-nitrophenoxy)ethoxy)ethoxy)ethoxy)ethyl)amino)-1-oxoisoindolin-2-

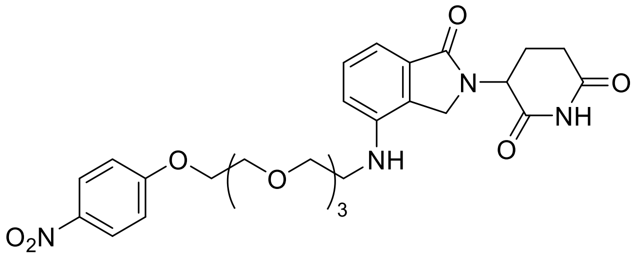

yl)piperidine-2,6-dione

To a solution of 3-(4-amino-1-oxoisoindolin-2-yl)piperidine-2,6-dione (44.6 mg, 0.17 mmol), 1-(2-(2-(2-(2-iodoethoxy)ethoxy)ethoxy)ethoxy)-4-nitrobenzene (87.7 mg, 0.21 mmol) in DMF (2.0 mL) was added *N*,*N*-Diisopropylethylamine (87.8 µL, 0.52 mmol) at room temperature. The resulting mixture was stirred at 100 °C for 12 h. Then the mixture was cooled to room temperature and the solvent was removed under reduced pressure. The residue was purified by flash column chromatography (CH_2Cl_2/MeOH = 100/0 to 20/1) to give the title product as a yellow solid (39.2 mg, 41% yield).

^{1}H-NMR (300MHz, $CDCl_3$) δ: 8.51 (s, 1H), 8.18 (t like, *J* = 9.1 Hz, 2H), 7.34 (t like, *J* = 7.8 Hz, 1H), 7.00-6.92 (m, 2H), 6.78 (d, *J* = 7.9 Hz, 1H), 5.21 (dd, *J* = 13.0, 5.1 Hz, 1H), 4.26-4.12 (m, 4H), 3.91-3.84 (m, 2H), 3.76-3.56 (m, 12H), 3.45-3.39 (m, 2H), 2.94-2.73 (m, 1H), 2.35-2.12 (m, 2H). **MS (ESI)** *m/z*: $[M+Na]^+$ 579.

2-((*S*)-4-(4-chlorophenyl)-2,3,9-trimethyl-6H-thieno[3,2-f][1,2,4]triazolo[4,3-a][1,4]diazepin-6-yl)-*N*-(4-(2-(2-(2-(2-((2-(2,6-dioxopiperidin-3-yl)-1-oxoisoindolin-4-yl)amino)ethoxy)ethoxy)ethoxy)ethoxy)phenyl)acetamide (NE-015)

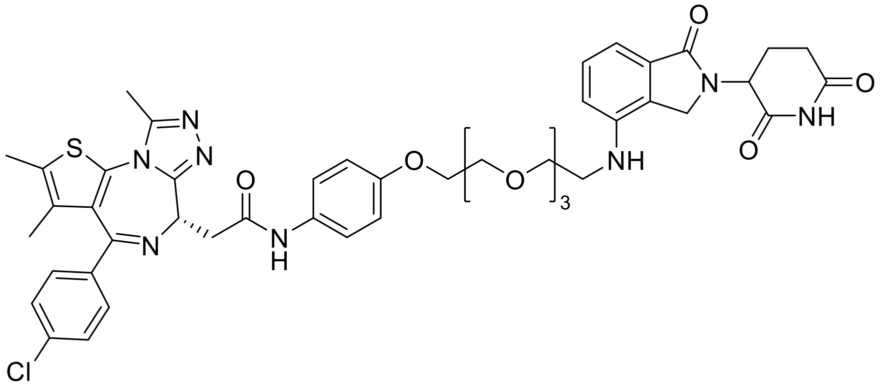

To a solution of 3-(4-((2-(2-(2-(2-(4-nitrophenoxy)ethoxy)ethoxy)ethoxy)ethyl)amino)-1-oxoisoindolin-2-yl)piperidine-2,6-dione (51.0 mg, 0.092 mmol), iron powder (51.3 mg, 0.92 mmol) in EtOH (1.5 mL) was added NH_4Cl aq (0.4 mL, [NH_4Cl] = 2.5 M) at room temperature. The mixture was refluxed at 80 °C for 1 h and cooled to room temperature. The mixture was filtered through a pad of celite, and the filtrate was extracted with EtOAc. The combined organic layers were washed with brine and dried over Na_2SO_4 and filtered, concentrated under reduced pressure to give the crude (47.8 mg), which was used next reaction without further purification.

The crude (23.5 mg) and (*S*)-2-(4-(4-chlorophenyl)-2,3,9-trimethyl-6H-thieno[3,2-f][1,2,4]triazolo[4,3-a][1,4]diazepin-6-yl)acetic acid (18.0 mg, 0.045 mmol) in DMF (0.50 mL) was added *N*,*N*-Diisopropylethylamine (23 μL, 0.13 mmol) and HATU (34.2 mg, 0.090 mmol) at room temperature. The resulting mixture was stirred at room temperature for 1 h. When the reaction was complete, H_2O was added to the mixture, and the mixture was extracted with EtOAc. The combined organic layers were washed with brine and dried over Na_2SO_4 and, filtered, concentrated under reduced pressure. The crude mixture was purified by HPLC separation using NOMURA CHEMICAL Develosil ODS-HG-5, 20×250 mm (Conditions: CH_3CN/H_2O = 50/50, flow rate = 5.0 mL/min, λ= 220 nm, t_R = 28.6 min) and freeze-drying to give the title product as a white solid (21.7 mg, 56% yield, 2 steps).

^{1}H-NMR (300MHz, CD_3OD) δ: 7.50-7.40 (m, 5H), 7.38-7.28 (m, 2H), 7.10-7.06 (m, 1H), 6.90-6.86 (m, 3H), 5.12 (d, *J* = 8.2 Hz, 1H), 4.73-4.67 (m, 1H), 4.28-4.26 (m, 2H), 4.08-4.04 (m, 2H), 3.82-3.75 (m, 2H), 3.72-3.60 (m, 12H), 3.40-3.36 (m, 2H), 2.80-2.65 (m, 5H), 2.45-2.40 (m, 4H), 2.15-2.10 (m, 1H), 1.69 (s, 3H). **HRMS (ESI$^+$)**: *m/z* calcd. for $C_{46}H_{50}N_8O_8SCl$ $[M+H]^+$: 909.3160 found 909.3141.

3-(6-fluoro-4-((2-(2-(2-(2-(4-nitrophenoxy)ethoxy)ethoxy)ethoxy)ethyl)amino)-1-oxoisoindolin-2-yl)piperidine-2,6-dione

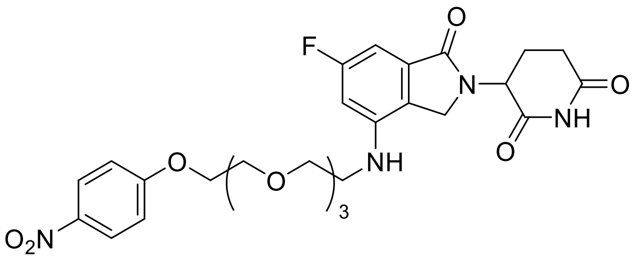

To a solution of 3-(4-amino-6-fluoro-1-oxoisoindolin-2-yl)piperidine-2,6-dione (50.0 mg, 0.17 mmol), 1-(2-(2-(2-(2-iodoethoxy)ethoxy)ethoxy)ethoxy)-4-nitrobenzene (92.0 mg, 0.22 mmol) in DMF (2.0 mL) was added *N*,*N*-Diisopropylethylamine (92 μL, 0.54 mmol) at room temperature. The resulting mixture was stirred at 100 °C for 12 h. The mixture was cooled to room temperature, and the solvent was removed under reduced pressure. The residue was purified by flash column chromatography (EtOAc/MeOH = 100/0 to 10/1) to give the title product as a yellow solid (36.9 mg, 36% yield).

^{1}H-NMR (300MHz, $CDCl_3$) δ: 8.24-8.13 (m, 3H), 7.03-6.92 (m, 3H), 6.50 (dd, *J* = 11.4, 2.1 Hz, 1H), 5.23 (dd, *J* = 13.0, 5.4 Hz, 1H), 4.33-4.12 (m, 4H), 3.94-3.88 (m, 2H), 3.77-3.63 (m, 10H), 3.39-3.35 (m, 2H), 2.99-2.84 (m, 2H), 2.54-2.54 (m, 1H), 2.37-2.25 (m, 2H), 1.79-1.79 (m, 1H). **^{19}F-NMR (282MHz, $CDCl_3$)** δ: -111.1 (t like, *J* = 9.4 Hz, 1F). **MS (ESI)** *m/z*: $[M+Na]^+$ 597.

2-((*S*)-4-(4-chlorophenyl)-2,3,9-trimethyl-6H-thieno[3,2-f][1,2,4]triazolo[4,3-a][1,4]diazepin-6-yl)-*N*-(4-(2-(2-(2-(2-((2-(2,6-dioxopiperidin-3-yl)-6-fluoro-1-oxoisoindolin-4-yl)amino)ethoxy)ethoxy)ethoxy)ethoxy)phenyl)acetamide (NE-018)

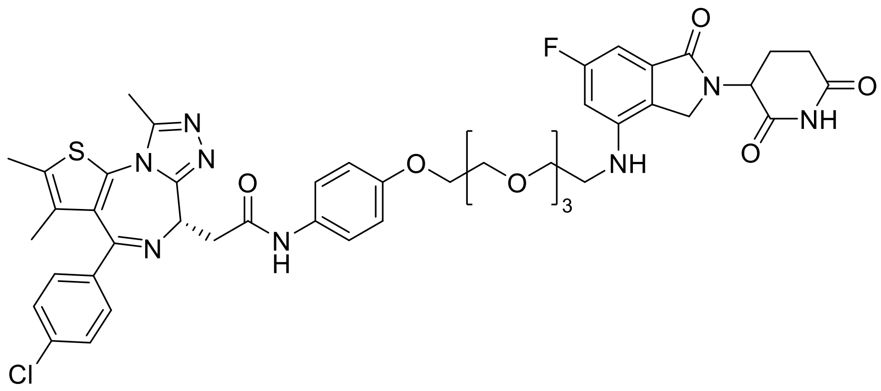

To a solution of 3-(6-fluoro-4-((2-(2-(2-(2-(4-nitrophenoxy)ethoxy)ethoxy)ethoxy)ethyl)amino)-1-oxoisoindolin-2-yl)piperidine-2,6-dione (36.9 mg, 0.064 mmol), iron powder (35.9 mg, 0.64 mmol) in EtOH (1.0 mL) was added NH_4Cl aq (0.5 mL, [NH_4Cl] = 1.3 M) at room temperature. The mixture was refluxed at 80 °C for 2 h and cooled to room temperature. The mixture was filtered through a pad of celite, and the filtrate was extracted with EtOAc. The combined organic layers were washed with brine and dried over Na_2SO_4 and filtered, concentrated under reduced pressure to give the crude (25.8 mg), which was used next reaction without further purification.

the crude (17.4 mg) and (*S*)-2-(4-(4-chlorophenyl)-2,3,9-trimethyl-6H-thieno[3,2-f][1,2,4]triazolo[4,3-a][1,4]diazepin-6-yl)acetic acid (12.6 mg, 0.031 mmol) in DMF (0.5 mL) was added *N*,*N*-Diisopropylethylamine (16 µL, 0.094 mmol) and HATU (17.9 mg, 0.047 mmol) at room temperature. The resulting mixture was stirred at room temperature for 15 h. When the reaction was complete, H_2O was added to the mixture, and the mixture was extracted with EtOAc. The combined organic layers were washed with brine and, dried over Na_2SO_4 and filtered, concentrated under reduced pressure. The crude mixture was purified by HPLC separation using NOMURA CHEMICAL Develosil ODS-HG-5, 20×250 mm (Conditions: CH_3CN/H_2O = 10/90 to 40/60 with 0.1% TFA, flow rate = 5.0 mL/min, λ= 220 nm) and freeze-drying to give the title product as a white solid (9.5 mg, 24% yield, 2 steps).

^{1}H-NMR (300 MHz, CD_3OD) δ: 7.50-7.35 (m, 6H), 6.87 (d, *J* = 9.0 Hz, 2H), 6.70 (dd, *J* = 7.6, 2.1 Hz, 1H), 6.58 (dd, *J* = 12.2, 2.1 Hz, 1H), 5.10 (dd, *J* = 13.2, 5.1 Hz, 1H), 4.73-4.67 (m, 1H), 4.25-4.23 (m, 2H), 4.08-4.04 (m, 2H), 3.82-3.75 (m, 2H), 3.73-3.59 (m, 12H), 3.38-3.32 (m, 2H), 2.75-2.70 (m, 5H), 2.49-2.35 (m, 4H), 2.16-2.09 (m, 1H), 1.69 (s, 3H). **^{19}F-NMR (282 MHz, CD_3OD)** δ: -107.8 (dd, *J* = 11.9, 7.9 Hz, 1F). **HRMS (ESI$^+$)**: *m/z* calcd. for $C_{46}H_{49}N_8O_8FSCl$ $[M+H]^+$: 927.3067 found 927.3024.

3-(6-chloro-4-((2-(2-(2-(2-(4-nitrophenoxy)ethoxy)ethoxy)ethoxy)ethyl)amino)-1-oxoisoindolin-2-yl)piperidine-2,6-dione

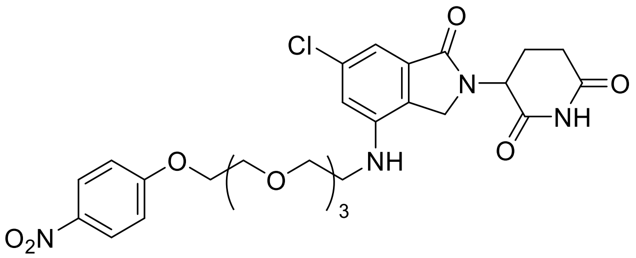

To a solution of 3-(4-amino-6-chloro-1-oxoisoindolin-2-yl)piperidine-2,6-dione (58.7 mg, 0.20 mmol), 1-(2-(2-(2-(2-iodoethoxy)ethoxy)ethoxy)ethoxy)-4-nitrobenzene (340.2 mg, 0.80 mmol) in DMF (1.0 mL) was added *N*,*N*-Diisopropylethylamine (0.10 mL, 0.60 mmol) at room temperature. The resulting mixture was stirred at 110 °C for 24 h. The mixture was cooled to room temperature, and the solvent was removed under reduced pressure. The residue was purified by flash column chromatography (EtOAc/MeOH = 20/1) to give the title product as a pale yellow solid (58.5 mg, 49% yield).

^{1}H-NMR (300MHz, $CDCl_3$) δ: 8.55 (s, 1H), 8.15 (d, *J* = 12.1 Hz, 2H), 7.16 (s, 1H), 6.92 (d, *J* = 12.2 Hz, 2H), 6.69 (s, 1H), 5.15 (dd, *J* = 12.9, 5.3 Hz, 1H), 4.33 (s, 1H), 4.26-4.07 (m, 4H), 3.84 (t, *J* = 4.6 Hz, 2H), 3.74-3.54 (m, 10H), 3.33 (d like, *J* = 4.1 Hz, 2H), 2.87-2.71 (m, 2H), 2.30-2.10 (m, 2H). **MS (ESI)** *m/z*: $[M+Na]^+$ 613.

***N*-(4-(2-(2-(2-(2-((6-chloro-2-(2,6-dioxopiperidin-3-yl)-1-oxoisoindolin-4-yl)amino)ethoxy)ethoxy)ethoxy)ethoxy)phenyl)-2-((*S*)-4-(4-chlorophenyl)-2,3,9-trimethyl-6H-thieno[3,2-f][1,2,4]triazolo[4,3-a][1,4]diazepin-6-yl)acetamide** (NE-019)

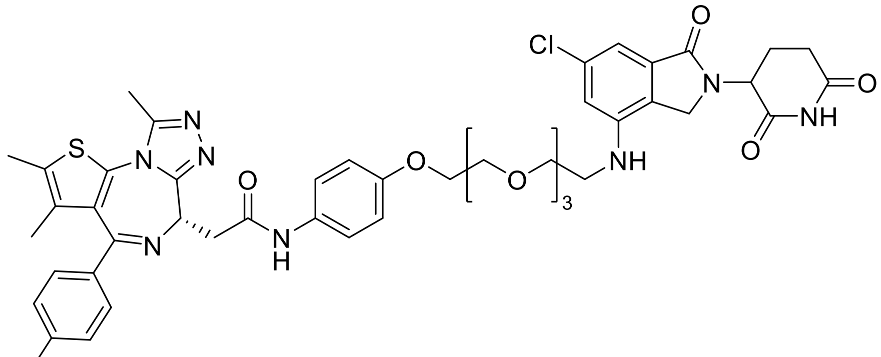

To a solution of 3-(6-chloro-4-((2-(2-(2-(2-(4-nitrophenoxy)ethoxy)ethoxy)ethoxy)ethyl)amino)-1-oxoisoindolin-2-yl)piperidine-2,6-dione (58.5 mg, 0.099 mmol), iron powder (55.2 mg, 0.99 mmol) in EtOH (1.7 mL) was added NH_4Cl aq (0.4 mL, [NH_4Cl] = 2.5 M) at room temperature. The mixture was refluxed at 80 °C for 1 h and cooled to room temperature. The mixture was filtered through a pad of celite, and the filtrate was extracted with EtOAc. The combined organic layers were washed with brine and dried over Na_2SO_4 and filtered, concentrated under reduced pressure to give the crude (46.8 mg), which was used next reaction without further purification.

The crude (46.8 mg) and (*S*)-2-(4-(4-chlorophenyl)-2,3,9-trimethyl-6H-thieno[3,2-f][1,2,4]triazolo[4,3-a][1,4]diazepin-6-yl)acetic acid (33.3 mg, 0.083 mmol) in DMF (0.8 mL) was added *N*,*N*-Diisopropylethylamine (43 μL, 0.25 mmol) and HATU (63.1 mg, 0.17 mmol). The resulting mixture was stirred at room temperature for 1 h. When the reaction was complete, H_2O was added to the mixture, and the mixture was extracted with EtOAc. The combined organic layers were washed with brine and, dried over Na_2SO_4 and filtered, concentrated under reduced pressure. The crude mixture was purified by HPLC separation using NOMURA CHEMICAL Develosil ODS-HG-5, 20×250 mm (Conditions: CH_3CN/H_2O = 10/90 to 60/40 with 0.1% TFA, flow rate = 5.0 mL/min, λ= 220 nm, t_R = 69.6 min) and freeze-drying to give the title product as a yellow solid (64.0 mg, 68% yield, 2 steps).
^{1}H-NMR (300 MHz, CD_3OD) δ: 7.59-7.30 (m, 6H), 7.02-6.93 (m, 1H), 6.85-6.78 (m, 3H), 5.06 (dd, *J* = 13.2, 5.0 Hz, 1H), 4.84-4.75 (m, 1H), 4.28-4.15 (m, 2H), 4.09-4.02 (m, 2H), 3.85-3.75 (m, 2H), 3.68-3.48 (m, 12H), 3.35-3.19 (m, 2H), 2.89-2.67 (m, 5H), 2.48-2.22 (m, 4H), 2.11-2.01 (m, 1H), 1.68 (s, 3H). **MS (ESI)** *m/z*: $[M+H]^+$ 943.

3-(4-((2-(2-(2-(2-(4-nitrophenoxy)ethoxy)ethoxy)ethoxy)ethyl)amino)-1-oxo-6-(trifluoromethyl)isoindolin-2-yl)piperidine-2,6-dione

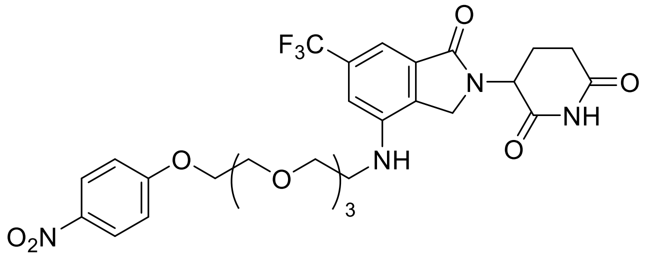

To a solution of 3-(4-amino-1-oxo-6-(trifluoromethyl)isoindolin-2-yl)piperidine-2,6-dione (65.5 mg, 0.20 mmol), 1-(2-(2-(2-(2-iodoethoxy)ethoxy)ethoxy)ethoxy)-4-nitrobenzene (255.1 mg, 0.60 mmol) in NMP (1.0 mL) was added *N*,*N*-Diisopropylethylamine (0.10 mL, 0.60 mmol) at room temperature. The resulting mixture was stirred at 110 °C for 12 h. The mixture was cooled to room temperature, and the solvent was removed under heating and reduced pressure. The residue was purified by flash column chromatography (EtOAc/MeOH = 100/0 to 5/1) to give the title product as a white solid (46.3 mg, 41% yield).
^{1}H-NMR (300MHz, $CDCl_3$) δ: 8.71 (s, 1H), 8.15-8.10 (m, 2H), 7.44 (s, 1H), 6.98-6.90 (m, 3H), 5.18 (dd, *J* = 12.9, 5.3 Hz, 1H), 4.49 (s, 1H), 4.35-4.15 (m, 4H), 3.84 (t, *J* = 4.6 Hz, 2H), 3.74 (t, *J* = 4.9 Hz, 2H), 3.68-3.54 (m, 8H), 3.37 (m, 2H), 2.85-2.71 (m, 2H), 2.39-1.89 (m, 2H). **^{19}F-NMR (282MHz, $CDCl_3$)** δ: -62.84 (s, 3F). **MS (ESI)** *m/z*: $[M+Na]^+$ 647.

2-((*S*)-4-(4-chlorophenyl)-2,3,9-trimethyl-6H-thieno[3,2-f][1,2,4]triazolo[4,3-a][1,4]diazepin-6-yl)-*N*-(4-(2-(2-(2-(2-((2-(2,6-dioxopiperidin-3-yl)-1-oxo-6-(trifluoromethyl)isoindolin-4-yl)amino)ethoxy)ethoxy)ethoxy)ethoxy)phenyl)acetamide (NE-020)

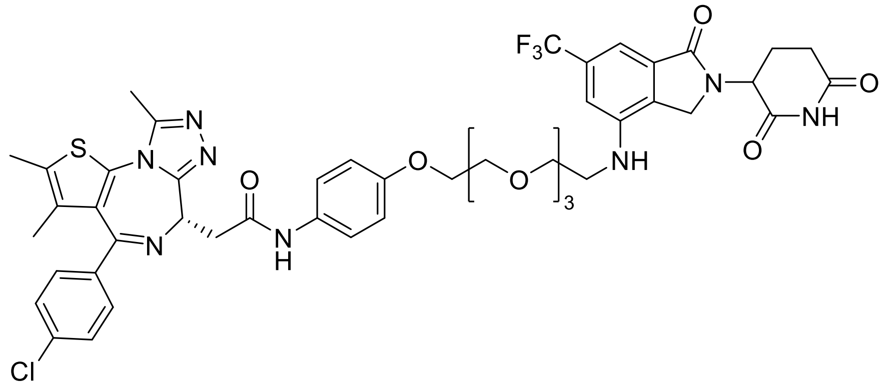

To a solution of 3-(4-((2-(2-(2-(2-(4-nitrophenoxy)ethoxy)ethoxy)ethoxy)ethyl)amino)-1-oxo-6-(trifluoromethyl)isoindolin-2-yl)piperidine-2,6-dione (46.3 mg, 0.074 mmol), iron powder (41.3 mg, 0.74 mmol) in EtOH (1.2 mL) was added NH_4Cl aq (0.3 mL, [NH_4Cl] = 2.5 M) at room temperature. The mixture was refluxed at 80 °C for 1 h and cooled to room temperature. The mixture was filtered through a pad of celite, and the filtrate was extracted with EtOAc. The combined organic layers were washed with brine and dried over Na_2SO_4 and filtered, concentrated under reduced pressure to give the crude (37.5 mg), which was used next reaction without further purification.

The crude (37.5 mg) and (*S*)-2-(4-(4-chlorophenyl)-2,3,9-trimethyl-6H-thieno[3,2-f][1,2,4]triazolo[4,3-a][1,4]diazepin-6-yl)acetic acid (25.3 mg, 0.063 mmol) in DMF (0.6 mL) was added *N*,*N*-Diisopropylethylamine (33 μL, 0.19 mmol) and HATU (47.9 mg, 0.13 mmol). The resulting mixture was stirred at room temperature for 2 h. When the reaction was complete, H_2O was added to the mixture, and the mixture was extracted with EtOAc. The combined organic layers were washed with brine and, dried over Na_2SO_4 and filtered, concentrated under reduced pressure. The crude mixture was purified by HPLC separation using NOMURA CHEMICAL Develosil ODS-HG-5, 20×250 mm (Conditions: CH_3CN/H_2O = 10/90 to 90/10 with 0.1% TFA, flow rate = 5.0 mL/min, λ= 220 nm, t_R = 39.5 min) and freeze-drying to give the title product as a yellow solid (41.0 mg, 57% yield, 2 steps)

^{1}H-NMR (300MHz, CD_3OD) δ: 7.48-7.39 (m, 6H), 7.27 (s, 1H), 7.03 (s, 1H), 6.84 (d, *J* = 8.8 Hz, 2H), 5.14-5.09 (m, 1H), 4.79 (d, *J* = 5.6 Hz, 1H), 4.33 (s, 2H), 4.04 (t, *J* = 4.4 Hz, 2H), 3.79-3.76 (m, 2H), 3.71-3.53 (m, 12H), 3.42 (t, *J* = 5.0 Hz, 2H), 2.86-2.70 (m, 5H), 2.45-2.37 (m, 4H), 2.14 (s, 1H), 1.69 (s, 3H). **^{19}F-NMR (282MHz, $CDCl_3$)** δ: -62.89 (s, 3F). **MS (ESI)** *m/z*: $[M+H]^+$ 977.

Supplementary References

1) Tang, K.; Hsu W.; Chen, C.; Tsai, M.; Yen, C.; and Tseng, C.; Discovery of triazolyl thalidomide derivatives as anti-fibrosis agents. *New J. Chem.*, **2021**, *45*, 3589−3599.
2) Chaulet, C.; Croix, C.; Alagille, D.; Normand, S.; Delwail, A.; Favot, L.; Lecron, J.-C.; Viaud-

Massuard, M.-C. Design, synthesis and biological evaluation of new thalidomide analogues as TNF-α and IL-6 production inhibitors. *Bioorg. Med. Chem. Lett.* **2011**, *21*, 1019−1022.

3) Steinebach, C.; Lindner, S.; Udeshi, N. D.; Mani, D. C.; Kehm, H.; Köpff, S.; Carr, S. A.; Gütschow, M.; Krönke, J. Homo-PROTACs for the Chemical Knockdown of Cereblon. *ACS Chem. Biol.* **2018**, *13*, 2771−2782.

4) G. M. Burslem, P. Ottis, S. Jaime-Figueroa, A. Morgan, P. M. Cromm, M. Toure, C. M. Crews, Phenyl-Glutarimides: Alternative Cereblon Binders for the Design of PROTACs. *ChemMedChem*., **2018**, *13*, 1508–1512.

5) Wurz, R. P.; Dellamaggiore, K.; Dou, H.; Javier, N.; Lo, M. C.; McCarter, J. D.; Mohl, D.; Sastri, C.; Lipford, J. R.; Cee, V. J. A "Click Chemistry Platform" for the Rapid Synthesis of Bispecific Molecules for Inducing Protein Degradation. *J. Med. Chem.* **2018**, *61*, 453−461.

6) Hanafi, M.; Chen, X.; Neamati, N. Discovery of a napabucasin PROTAC as an effective degrader of the E3 ligase ZFP91. *J. Med. Chem.* **2021**, *64*, 1626−1648.
