# Supplementary material for: Lenalidomide derivatives and proteolysis-targeting chimeras for controlling neosubstrate degradation
Source: Nat Commun. 2023 Aug 18;14:4683. doi: 10.1038/s41467-023-40385-9 (PMC10439208; doi:10.1038/s41467-023-40385-9)
Supplement: Supplementary file 2 — Reporting Summary [file 41467_2023_40385_MOESM2_ESM.pdf]

nature portfolio

Corresponding author(s): Tatsuya Sawasaki

Last updated by author(s): Jul 11, 2023

Reporting Summary

Nature Portfolio wishes to improve the reproducibility of the work that we publish. This form provides structure for consistency and transparency in reporting. For further information on Nature Portfolio policies, see our Editorial Policies and the Editorial Policy Checklist.

Statistics

For all statistical analyses, confirm that the following items are present in the figure legend, table legend, main text, or Methods section.

n/a	Confirmed	
☐	☒	The exact sample size (*n*) for each experimental group/condition, given as a discrete number and unit of measurement
☐	☒	A statement on whether measurements were taken from distinct samples or whether the same sample was measured repeatedly
☐	☒	The statistical test(s) used AND whether they are one- or two-sided<br>*Only common tests should be described solely by name; describe more complex techniques in the Methods section.*
☒	☐	A description of all covariates tested
☐	☒	A description of any assumptions or corrections, such as tests of normality and adjustment for multiple comparisons
☐	☒	A full description of the statistical parameters including central tendency (e.g. means) or other basic estimates (e.g. regression coefficient) AND variation (e.g. standard deviation) or associated estimates of uncertainty (e.g. confidence intervals)
☐	☒	For null hypothesis testing, the test statistic (e.g. *F*, *t*, *r*) with confidence intervals, effect sizes, degrees of freedom and *P* value noted<br>*Give P values as exact values whenever suitable.*
☒	☐	For Bayesian analysis, information on the choice of priors and Markov chain Monte Carlo settings
☒	☐	For hierarchical and complex designs, identification of the appropriate level for tests and full reporting of outcomes
☒	☐	Estimates of effect sizes (e.g. Cohen's *d*, Pearson's *r*), indicating how they were calculated

Our web collection on statistics for biologists contains articles on many of the points above.

Software and code

Policy information about availability of computer code

Data collection	All data collection in this study were performed using softwares attached to each detector.<br>Image Quant LAS 4000 software (GE Healthcare, version 1.1) for chemical luminescent immunoblot.<br>Odyssey Fc Image Studio software (LI-COR Biosciences, version 5.2) for fluorescent immunoblot.<br>Wallac Envision Manager software (ParkinElmer, version 1.12) for AlphaScreen.<br>LightCycler 96 software (Roche, version 1.1) for quantitative-PCR.<br>SpectraMax iD3 software (Molecular Devise, version 7.1) for CellTiter-Glo.<br>MicroCal ITC software for ITC experiments.<br>Proteome Discoverer software (Thermo Fisher Scientific, version 2.4.1.15) for LC-MS/MS.
Data analysis	Image analysis was performed using ImageJ (Fiji) software (version 2.1.0).<br>Empiria Studio software (version 1.3) was used for fluorescent immunoblot analysis.<br>Origin software (7.0) was used for analysing ITC data.<br>Data analysis and significant changes were performed using Excel (version 16.66) or GraphPad Prism 9 (Version 9.5.1).<br>Docking simulation and structure depiction were performed using AutoDock Vina (The Scripts Research Institute, version 1.1.2), AutoDockTools (The Scripts Research Institute, version 1.5.6), PyMOL (Schrödinger, LLC, version 2.4.0), and UCSF Chimera (RBVI at the University of California, San Fransisco, version 1.14).

For manuscripts utilizing custom algorithms or software that are central to the research but not yet described in published literature, software must be made available to editors and reviewers. We strongly encourage code deposition in a community repository (e.g. GitHub). See the Nature Portfolio guidelines for submitting code & software for further information.

Data

Policy information about availability of data

All manuscripts must include a data availability statement. This statement should provide the following information, where applicable:

- Accession codes, unique identifiers, or web links for publicly available datasets
- A description of any restrictions on data availability
- For clinical datasets or third party data, please ensure that the statement adheres to our policy

The MS proteomics data have been provided in Supplementary Data 1–3 and deposited to the ProteomeXchange Consortium via the jPOST partner respository with the dataset identifiers PXD041812 [https://repository.jpostdb.org/preview/5179484996448a021cabb7] (access key, 5793) (TMTpro-18plex analysis of MM1.S cells treated with thalidomide and its derivatives), PXD037178 [https://repository.jpostdb.org/preview/5570632816375c0e8f3956] (access key, 4221) (TMTpro-18plex analysis of MM1.S cells treated with PROTACs) and PXD037179 [https://repository.jpostdb.org/preview/15513902676375c0e6587cc] (access key, 9287) (TMTpro-18plex analysis of NTERA-2 cells treated with PROTACs). All data supporting the findings of this study are provided in the main text, supplementary information, or source data.

Human research participants

Policy information about studies involving human research participants and Sex and Gender in Research.

Reporting on sex and gender	N/A
Population characteristics	N/A
Recruitment	N/A
Ethics oversight	N/A

Note that full information on the approval of the study protocol must also be provided in the manuscript.

Field-specific reporting

Please select the one below that is the best fit for your research. If you are not sure, read the appropriate sections before making your selection.

☒ Life sciences ☐ Behavioural & social sciences ☐ Ecological, evolutionary & environmental sciences

For a reference copy of the document with all sections, see nature.com/documents/nr-reporting-summary-flat.pdf

Life sciences study design

All studies must disclose on these points even when the disclosure is negative.

Sample size	No statiscal methods were used for sample size. All statistical calculations were defined from at least triplicates. For immunoblot analyses and LC-MS/MS analyses, such as proteasomal degradation of neosubstrate and streptavidin pull-down assays, we have chosen more than two independent experiments as sample sizes based on our previous papers in same research field (Yamanaka et al., Nat. Commun. 2022;13:e183, Yamanaka et al., EMBO J. 2021;40(4):e105375, Furihata et al., Nat. Commun. 2020;11(1):457 and Kido et al., eLife 2020;9:e54983) and traditional experimental approach in biochemical and cellular experiments.
Data exclusions	No data were excluded from the analyses.
Replication	Numbers of replicates are described in the figure legends.
Randomization	Randomization was not relevant because there is no allocation of samples/organisms/participants involved in this study.
Blinding	Blinding was not necessary because there is no group allocation involved in this study.

Reporting for specific materials, systems and methods

We require information from authors about some types of materials, experimental systems and methods used in many studies. Here, indicate whether each material, system or method listed is relevant to your study. If you are not sure if a list item applies to your research, read the appropriate section before selecting a response.

Materials & experimental systems

n/a	Involved in the study
☐	☒ Antibodies
☐	☒ Eukaryotic cell lines
☒	☐ Palaeontology and archaeology
☒	☐ Animals and other organisms
☒	☐ Clinical data
☒	☐ Dual use research of concern

Methods

n/a	Involved in the study
☒	☐ ChIP-seq
☒	☐ Flow cytometry
☒	☐ MRI-based neuroimaging

Antibodies

Antibodies used

Anti-FLAG mouse mAb clone M2 (HRP-conjugated, Sigma-Aldrich, #A8592, 1:5000), anti-AGIA rabbit mAb (HRP-conjugated, produced in our laboratory, 1:5000), anti-HA-tag rat mAb clone 3F10 (HRP-conjugated, Roche, #12013819001, 1:5000), and anti-Myc-tag mAb clone 9B11 (HRP-conjugated, Cell Signaling Technology, #2040, 1:1000) were used to detect epitope-tagged proteins. Anti-α-tubulin rabbit pAb (HRP-conjugated, MBL, #PM054-7, 1:5000) and anti-α-tubulin mouse mAb (LI-COR Biosciences, #926-42213, 1:1000) were used to detect α-tubulin. Anti-GAPDH mouse mAb clone 3H12 (MBL, #M171-7, 1:5000) and anti-GAPDH rabbit mAb clone D16H11 (Cell Signaling Technology, #5174, 1:1000) were used to detect GAPDH. Biotinylated proteins were detected by anti-biotin goat pAb (HRP-conjugated, Cell Signaling Technology, #7075, 1:3000) or streptavidin (HRP-conjugated, Abcam, #ab7403, 1:5000). Anti-CRBN rabbit mAb clone D8H3S (Cell Signaling Technology, #71810, 1:1000), anti-IKZF1/Ikaros rabbit mAb clone D6N9Y (Cell Signaling Technology, #14859, 1:1000), IKZF2/Helios rabbit mAb clone D8W4X (Cell Signaling Technology, #42427, 1:1000), anti-IKZF3/Aiolos rabbit mAb clone D1C1E (Cell Signaling Technology, #15103, 1:1000), anti-cMYC rabbit mAb clone E5Q6W (Cell Signaling Technology, #18583, 1:1000), anti-IRF4 rabbit mAb clone E8H3S (Cell Signaling Technology, #62834, 1:1000), anti-BRD4 rabbit mAb clone E2A7X (Cell Signaling Technology, #13440, 1:1000), anti-p63-α rabbit mAb clone D2K8X (Cell Signaling Technology, #13109, 1:1000), anti-SALL4 mouse mAb clone EE-30 (Santa Cruz Biotechnology, #sc-101147, 1:500), anti-BRD3 mouse mAb clone 2088C3a (Santa Cruz Biotechnology, #sc-81202, 1:500), anti-PLZF goat pAb (R&D System, #AF2944, 1:1000), anti-BRD4 rabbit pAb (Bethyl Laboratories, #A301-985A, 1:1000), anti-BRD2 rabbit pAb (Bethyl Laboratories, #A302-583A, 1:1000), anti-ZFP91 rabbit pAb (Bethyl Laboratories, # A303-245A, 1:1000), anti-BRD4 rabbit pAb (Bethyl Laboratories, # A301-985A, 1:1000), anti-CK1α rabbit mAb clone ID EPR1961(2) (Abcam, #ab108296, 1:1000), anti-IKZF4/Eos rabbit pAb (ZeneTex, #GTX128043, 1:1000), anti-ZMYM2 rabbit pAb (ZeneTex, #GTX105550, 1:1000), anti-RAB28 rabbit pAb (ABclonal, #A17368, 1:500) and anti-RNF166 rabbit pAb (ABclonal, #A8276, 1:500) were used as primary antibodies. Anti-rabbit IgG (HRP-conjugated, Cell Signaling Technology, # 7074, 1:5000), anti-mouse IgG (HRP-conjugated, Cell Signaling Technology, # 7076, 1:5000), anti-goat IgG (HRP-conjugated, Invitrogen/Thermo Fisher Scientific, #81-1620, 1:10000), IRDye 800CW goat anti-rabbit IgG (LI-COR Biosciences, # 925-32211, 1:10000), IRDye 680RD goat anti-mouse IgG (LI-COR Biosciences, # 925-68070, 1:10000), IRDye 800CW goat anti-mouse IgG (LI-COR Biosciences, #925-32210, 1:10000) and IRDye 680RD goat anti-rabbit IgG (LI-COR Biosciences, #925-68071, 1:10000) were used as secondary antibodies.

Validation

All primary antibodies in this study were purchased from commercial companies. All of these antibodies have been validated for the human species as described on the supplier's websites.
The anti-CRBN rabbit mAb (Cell Signaling Technology, #71810, 1:1000), anti-IKZF1/Ikaros rabbit mAb (Cell Signaling Technology, #14859, 1:1000), IKZF2/Helios rabbit mAb (Cell Signaling Technology, #42427, 1:1000), anti-IKZF3/Aiolos rabbit mAb (Cell Signaling Technology, #15103, 1:1000), anti-cMYC rabbit mAb (Cell Signaling Technology, #18583, 1:1000), anti-IRF4 rabbit mAb (Cell Signaling Technology, #62834, 1:1000), anti-BRD4 rabbit mAb (Cell Signaling Technology, #13440, 1:1000), anti-GSPT1 rabbit pAb (Cell Signaling Technology, #14980, 1:1000), anti-p63-α rabbit mAb (Cell Signaling Technology, #13109, 1:1000) and anti-GAPDH rabbit mAb (Cell Signaling Technology, #5174, 1:1000) have been validated for detection of human species by immunoblot analysis as described on Cell Signaling Technology websites for specific antibodies.
The anti-SALL4 mouse mAb (Santa Cruz Biotechnology, #sc-101147, 1:500), anti-BRD3 mouse mAb (Santa Cruz Biotechnology, #sc-81202, 1:500), anti-PLZF goat pAb (R&D System, #AF2944, 1:1000), anti-BRD4 rabbit pAb (Bethyl Laboratories, # A301-985A, 1:1000), anti-BRD2 rabbit pAb (Bethyl Laboratories, # A302-583A, 1:1000), anti-CK1α rabbit mAb (Abcam, ab108296), anti-IKZF4/Eos rabbit pAb (ZeneTex, #GTX128043, 1:1000), anti-ZMYM2 rabbit pAb (ZeneTex, #GTX105550, 1:1000), anti-RAB28 rabbit pAb (ABclonal, #A17368, 1:500) and anti-RNF166 rabbit pAb (ABclonal, #A8276, 1:500) have been validated for detection of human species by immunoblot analysis as described on each supplier's website for specific antibodies.
The anti-FLAG mouse mAb (HRP-conjugated, Sigma-Aldrich, # A8592, 1:5000), anti-HA-tag rat mAb (HRP-conjugated, Roche, #12013819001, 1:5000) and anti-Myc-tag mAb (HRP-conjugated, Cell Signaling Technology, #2040, 1:1000) have been validated for detection of epitope-tagged proteins by immunoblot analysis as described on each supplier's website for specific antibodies.
The anti-α-tubulin rabbit pAb (HRP-conjugated, MBL, # PM054-7, 1:5000) and anti-α-tubulin mouse mAb (LI-COR Biosciences, # 926-42213, 1:1000) have been validated for detection of human α-tubulin by immunoblot analysis as described on each supplier's website. The anti-GAPDH mouse mAb (HRP-conjugated, MBL, #M171-7, 1:5000) has been validated for detection of human GAPDH by immunoblot analysis as described on supplier's website.
The anti-biotin goat pAb (HRP-conjugated, Cell Signaling Technology, # 7075, 1:3000) and streptavidin (HRP-conjugated, Abcam, #ab7403, 1:5000) have been validated for detection of biotinylated proteins by immunoblot analysis on supplier's website.
The anti-rabbit IgG (HRP-conjugated, Cell Signaling Technology, # 7074, 1:5000), anti-mouse IgG (HRP-conjugated, Cell Signaling Technology, # 7076, 1:5000), anti-goat IgG (HRP-conjugated, Invitrogen/Thermo Fisher Scientific, #81-1620, 1:10000), IRDye 800CW goat anti-rabbit IgG (LI-COR Biosciences, # 925-32211, 1:10000), IRDye 680RD goat anti-mouse IgG (LI-COR Biosciences, # 925-68070, 1:10000), IRDye 800CW goat anti-mouse IgG (LI-COR Biosciences, #925-32210, 1:10000) and IRDye 680RD goat anti-rabbit IgG (LI-COR Biosciences, #925-68071, 1:10000) have been validated as secondary antibody for immunoblot analysis as described on each supplier's website.
The AGIA antibody has been validated in published paper (Yano, et al., PLoS ONE 11: e0156716) cited in Methods.

Eukaryotic cell lines

Policy information about cell lines and Sex and Gender in Research

Cell line source(s)	MM1.S, U266, HEK293T, Jurkat and SU-DHL-4 cell lines were purchased from American Type Culture Collection (ATCC). HEK293T-CRBN-KO cell line was generated from HEK293T cell line purchased from Riken BioResource Research Center (Riken BRC) (Yamanaka et al., Commun. Biol. 2020;3:515). HCT116, KG-1a and THP-1 cell lines were purchased from Riken BRC. IMR32, MCF7, HuH7, RPMI8226, SKM-1, KG-1 and TK cell lines were purchased from Japanese Collection of Research Bioresources Cell Bank (JCRB Cell Bank). HaCaT cell line was obtained from CLS Cell Lines Service. BJAB cell lines were purchased from Deutsche Sammlung von Mikroorganismen und Zellkulturen (DSMZ). H929, Karpas-1106P and NTERA-2 cell lines were purchased from European Collection of Authenticated Cell Cultures (ECACC). MDS-L cell line originally established by Prof. K. Tohyama (Cytokines Cell Mol Ther 2000; 6: 61–70), and was kindly provided from Prof. K. Tohyama.
Authentication	All cell lines were used for each experiment between passage number 5 and 15 to avoid changes in the cell line's properties. In addition, all cell lines were authenticated by morphological appearances by careful observation.
Mycoplasma contamination	All cell lines used in this study were tested negative for mycoplasma.
Commonly misidentified lines (See ICLAC register)	No commonly misidentified cell lines were used in this study.
