# Supplementary material for: Lenalidomide derivatives and proteolysis-targeting chimeras for controlling neosubstrate degradation
Source: Nat Commun. 2023 Aug 18;14:4683. doi: 10.1038/s41467-023-40385-9 (PMC10439208; doi:10.1038/s41467-023-40385-9)
Supplement: Supplementary file 4 — Description of additional supplementary files [file 41467_2023_40385_MOESM4_ESM.docx]

**Description of Additional Supplementary Files Document**

**File Name: Supplementary Data 1**
Description: TMT-based quantitative proteomics in MM1.S cells treated with DMSO, thalidomide, pomalidomide, lenalidomide or 6-position-modified lenalidomides.

**File Name: Supplementary Data 2**Description: TMT-based quantitative proteomics in MM1.S cells treated with DMSO, ARV-825 or PROTACs based on 6-position-modified lenalidomides.

**File Name: Supplementary Data 3**Description: TMT-based quantitative proteomics in NTERA-2 cells treated with DMSO, ARV-825 or PROTACs based on 6-position-modified lenalidomides.
